# Development of spiro-3-indolin-2-one containing compounds of antiproliferative and anti-SARS-CoV-2 properties

**DOI:** 10.1038/s41598-022-17883-9

**Published:** 2022-08-16

**Authors:** Nehmedo G. Fawazy, Siva S. Panda, Ahmed Mostafa, Benson M. Kariuki, Mohamed S. Bekheit, Yassmin Moatasim, Omnia Kutkat, Walid Fayad, May A. El-Manawaty, Ahmed A. F. Soliman, Riham A. El-Shiekh, Aladdin M. Srour, Reham F. Barghash, Adel S. Girgis

**Affiliations:** 1 Department of Pesticide Chemistry, National Research Centre, Dokki, Giza, 12622 Egypt; 2 Department of Chemistry and Physics, Augusta University, Augusta, GA 30912 USA; 3 Center of Scientific Excellence for Influenza Viruses, National Research Centre, Giza, 12622 Egypt; 4 School of Chemistry, Cardiff University, Main Building, Park Place, Cardiff, CF10 3AT UK; 5 Drug Bioassay-Cell Culture Laboratory, Pharmacognosy Department, National Research Centre, Dokki, Giza, 12622 Egypt; 6 Department of Pharmacognosy, Faculty of Pharmacy, Cairo University, Cairo, 11562 Egypt; 7 Department of Therapeutic Chemistry, National Research Centre, Dokki, Giza, 12622 Egypt

**Keywords:** Chemical biology, Cancer

## Abstract

A series of 1″-(alkylsulfonyl)-dispiro[indoline-3,2′-pyrrolidine-3′,3″-piperidine]-2,4″-diones **6a‒o** has been synthesized through regioselective multi-component azomethine dipolar cycloaddition reaction of 1-(alkylsulfonyl)-3,5-bis(ylidene)-piperidin-4-ones **3a**‒**h**. X-ray diffraction studies (**6b‒d**,**h**) confirmed the structures. The majority of the synthesized analogs reveal promising antiproliferation properties against a variety of human cancer cell lines (MCF7, HCT116, A431 and PaCa2) with good selectivity index towards normal cell (RPE1). Some of the synthesized agents exhibit potent inhibitory properties against the tested cell lines with higher efficacies than the standard references (sunitinib and 5-fluorouracil). Compound **6m** is the most potent. Multi-targeted inhibitory properties against EGFR and VEGFR-2 have been observed for the synthesized agents. Flow cytometry supports the antiproliferation properties and shows the tested agents as apoptosis and necrosis forming. Vero cell viral infection model demonstrates the anti-SARS-CoV-2 properties of the synthesized agents. Compound **6f** is the most promising (about 3.3 and 4.8 times the potency of the standard references, chloroquine and hydroxychloroquine). QSAR models explain and support the observed biological properties.

## Introduction

**Figure 1:**
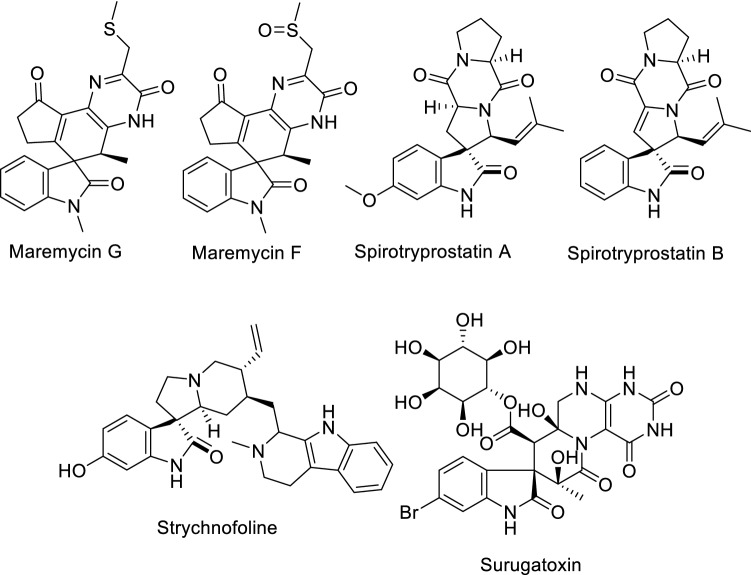
Natural spiro-indole containing compounds.

Compounds containing the spiro-indole framework occupy a unique place in the heterocyclic space that spans pharmaceutical and natural alkaloids^1,2^. Synthesis of spiro-heterocycles through the reactive carbonyl group is a subject of major interest for organic researchers^3^. Diverse synthetic methodologies have been reported for spiro-heterocycles, including intermolecular alkylation^4,5^, Morita–Baylis–Hillman^6,7^, 1,3-dipolar cycloaddition^8,9^, Mannich/Pictet–Spengler^3,10^, sigmatropic rearrangement^11,12^ and electrocyclization^13^ reactions. Many natural spiro-indoles with considerable biological properties have been identified, of which maremycin G (isolated from *Streptomyces *sp. B9173)^14,15^, maremycin F (isolated from *Streptomyces *sp. GT051237)^15,16^ spirotryprostatins A and B (isolated from *Aspergillus fumigatus*’ fermentation broth)^17^, strychnofoline (isolated from *Strychnos usambarensis*)^18,19^ and surugatoxin (isolated from ivory shell)^20^ reveal antimitotic activity (Fig. 1). Additionally, many synthetic spiro-indole analogs display considerable antimicrobial^21–24^, antitumor^8^ and cholinesterase inhibitory properties^25–28^.

**Figure 2:**
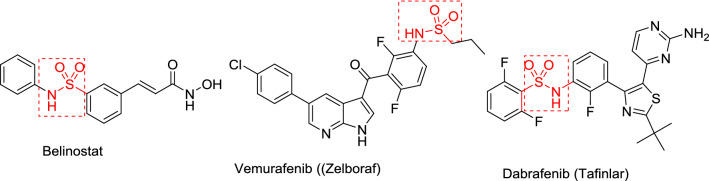
Approved antitumor sulfonamide-containing drugs.

The current study is directed towards synthesis and investigation of the biological properties of novel 3-spiro-indolin-2-ones prepared through azomethine dipolar cycloaddition to the exocyclic olefinic linkage of 3,5-bis(arylidene)-*N*-sulfonyl-4-piperidones. Interest in conjugation of the sulfonyl group to the heterocyclic nitrogen of 4-piperidone forming a sulfonamide fragment is due to the distinct physicochemical properties of the oxygen-rich sulfonyl residue that may increase the hydrophilicity of the bio-active agent^29,30^. Sulfonamides were flagged as attractive bio-active targets long ago (Gerhard Domagk, 1935) due to their antibacterial properties^31^. The sulfonamide motif can tether the bioactive agent in the targeted receptor/protein by forming hydrogen bonding with the neighboring amino acid functions. It is usually recognized as the bioisostere of the carboxylic group with limited drawbacks relative to the latter (metabolic toxicity, instability and capability for diffusion through bio-membranes)^31,32^. Many sulfonamides exert high efficacy as anticancer agents and have been approved as therapeutics. Belinostat [approved by Food and Drug Administration (FDA) in 2014] is used for treatment of peripheral T-cell lymphoma as a histone deacetylase inhibitor^33,34^. Vemurafenib (Zelboraf, approved by FDA in 2011 and 2017) treats BRAF V600 mutated late-stage skin cancer and Erdheim-Chester disease^35,36^. Dabrafenib (Tafinlar, approved in 2013 and 2018) is used for treatment of advanced melanoma and Mekinist also treats BRAF-positive cancer^37,38^ (Fig. 2). Many sulfonamide candidates have been reported as potential antitumor agents due to their aromatase, topoisomerase or carbonic anhydrase inhibitory properties^31,39–41^.

Cancer is one of the most severe diseases threatening human life. Although many methodologies and techniques in addition to numerous drugs have been discovered and approved for cancer treatment, millions of people continue to suffer from the illness every year. The off-target effect is one of the major drawbacks of cancer therapeutics^42^. Consequently, recent focus in cancer chemotherapy is on the development of highly selective anticancer agents devoid of off-target effects, thereby producing enhanced potency/efficacy towards the cancer cell with reduced side effects^31^.

**Figure 3:**
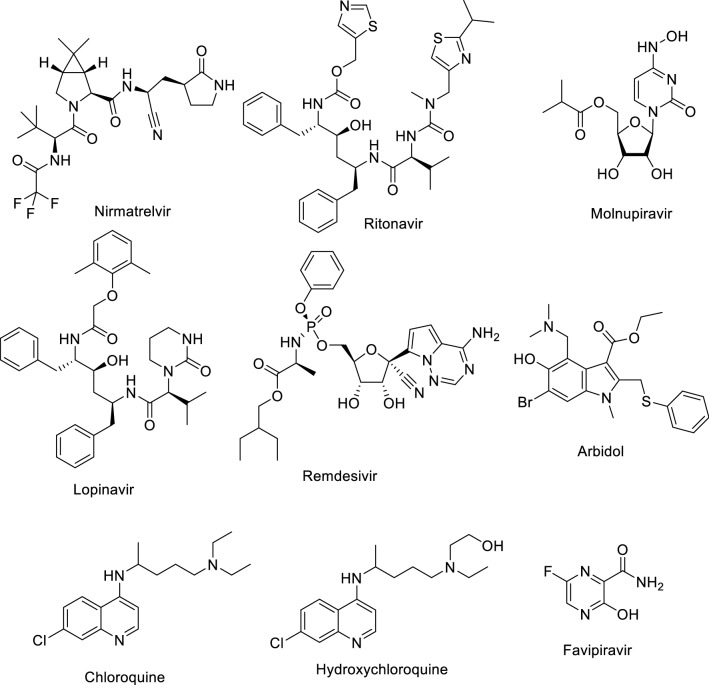
Drugs for treatment of COVID-19.

Owing to the anti-SARS-CoV-2 (severe acute respiratory syndrome coronavirus-2) properties of indole^43–48^ and sulfonamide-containing compounds^49^ the targeted agents within the current study are also considered for anti-SARS-CoV-2 investigation. In the beginning of 2020, a health-socio-economic disaster emerged globally due to the highly infectious disease. Severe acute respiratory syndrome due to pathogenic viral infection of SARS-CoV-2 leads to COVID-19 (coronavirus disease 2019). It is postulated that viral zoonotic jump (probably bat) to human was firstly recognized in Wuhan, China. The infection then spread worldwide causing a universal pandemic, according to WHO (World Health Organization) in March 2020^50,51^. Lack of an effective therapeutic was a major factor in the ensuing threat to human life. Several symptoms are associated with infection of which a cough, running noise, loss of smell and sometimes fever were highly publicized. In severe cases, blood clotting disorders and stroke are observed^52^. Drug repurposing was a wise and rapid strategy for urgent identification of potential therapeutics capable of controlling the disaster and saving lives. Usually, identification of novel drugs (de novo approach) with successive testing at pre-clinical and clinical phases takes several years. On the other hand, drug re-purposing allows faster adoption and utilization of well-studied existing and accessible therapeutics for treatment of infected patients^53^. Recently, Paxlovid (combination of Nirmatrelvir and Ritonavir) and Molnupiravir (Lagevrio) were approved (Dec. 2021) by the FDA emergency use authorization^54–57^ (Fig. 3). Recent publications have mentioned the effective treatment of COVID-19 patients with anticancer drugs^58,59^. The successful clinical trials of colon cancer patients with antiviral drugs alone or in combination with anticancer drugs^60^, also inspired the biological studies considered in the current work. Reported are the results of investigation of antiproliferation (against cancer cell lines) and antiviral (anti-SARS-CoV-2) properties of the targeted spiro-heterocycles with particular focus on safety-related effects on normal cells. Arbidol which is an indolyl scaffold (Fig. 3) and been used as anti-influenza drug, was recently re-purposed against SARS-CoV-2^61–65^. This is encouraging regarding the prospect of using the compounds from the current investigation against SARS-CoV-2 in addition to anti-tumor properties based on the mentioned bio-properties of the chemical scaffold considered.

## Results and discussion

### Chemical synthesis

The 1-(alkylsulfonyl)-3,5-bis(ylidene)-piperidin-4-ones **3a**‒**h** were obtained through dehydrohalogenation of the alkane sulfonyl chloride **2a**,**b** with the corresponding 3,5-bis(ylidene)-4-piperidinones **1a**‒**e** in dry tetrahydrofuran (THF) in the presence of triethylamine (TEA)^66^.

The azomethine ylide was obtained in situ through condensation of sarcosine **5** (secondary amino acid) and the appropriate isatin **4a**,**b**.

**Figure 4:**
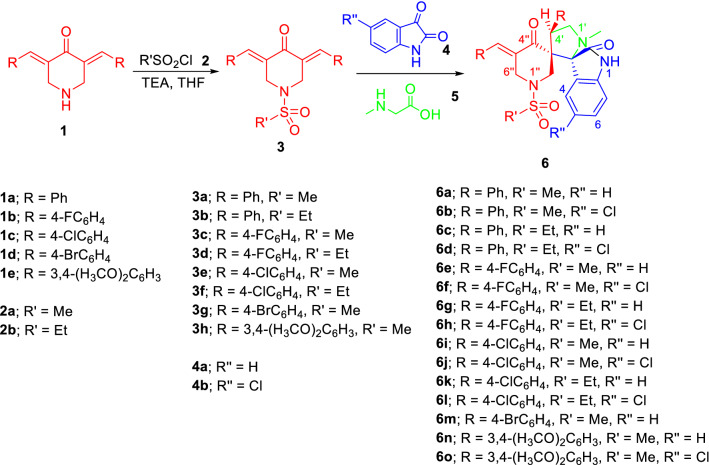
Synthesis of the targeted compounds **6a‒o**.

Multi-component dipolar cycloaddition reaction of **3a**‒**h** and azomethine ylide in refluxing ethanol afforded the targeted (*E*)-1″-(alkanesulfonyl)-4′-aryl-5″-arylidene-1′-methyl-dispiro[indoline-3,2′-pyrrolidine-3′,3″-piperidine]-2,4″-dione **6a**‒**o** (Fig. 4). Chemical structures of the synthesized agents were characterized by various spectroscopic techniques (IR, ^1^H-NMR and ^13^C-NMR) and elemental analysis data in addition to X-ray single crystallographic studies of representative examples (**6b**‒**6d** and **6h**). The IR spectrum of compound **6a** reveals the indolyl NH at *ν* = 3186 cm^−1^. The piperidinyl and indolyl carbonyls are observed at *ν* = 1705 and 1678 cm^−1^, respectively. The upfield protons of the diastereotopic piperidinyl H_2_C-2″ and H_2_C-6″ appear as doublet signals at *δ*_H_ = 2.25 and 3.54, respectively. The downfield protons of the piperidinyl H_2_C-2″ and H_2_C-6″ are overlapped as a multiplet signal at *δ*_H_ = 3.93‒3.98. The pyrrolidinyl H_2_C-5′ protons are also diastereotopic at *δ*_H_ = 3.37, 3.84 while the pyrrolidinyl methine proton HC-4′ is seen as a triplet signal at *δ*_H_ = 4.71. The ^13^C-NMR spectrum of **6a** shows the piperidinyl methylene carbons H_2_C-6″ and H_2_C-2″ at *δ*_C_ = 46.5 and 47.9, respectively. The pyrrolidinyl HC-4′, H_2_C-5′ carbons are observed at *δ*_C_ = 45.7 and 57.3, respectively. The spiro carbons C-3′ (C-3″), C-3 (C-2′) are located at *δ*_C_ = 61.2, 75.3, respectively. ^1^H, ^1^H-Cosy and HSQC spectra of a representative example (**6f**) support the assignments mentioned (Supplementary Figs. S1‒S47).

### Single crystal X-ray diffraction studies

**Figure 5:**
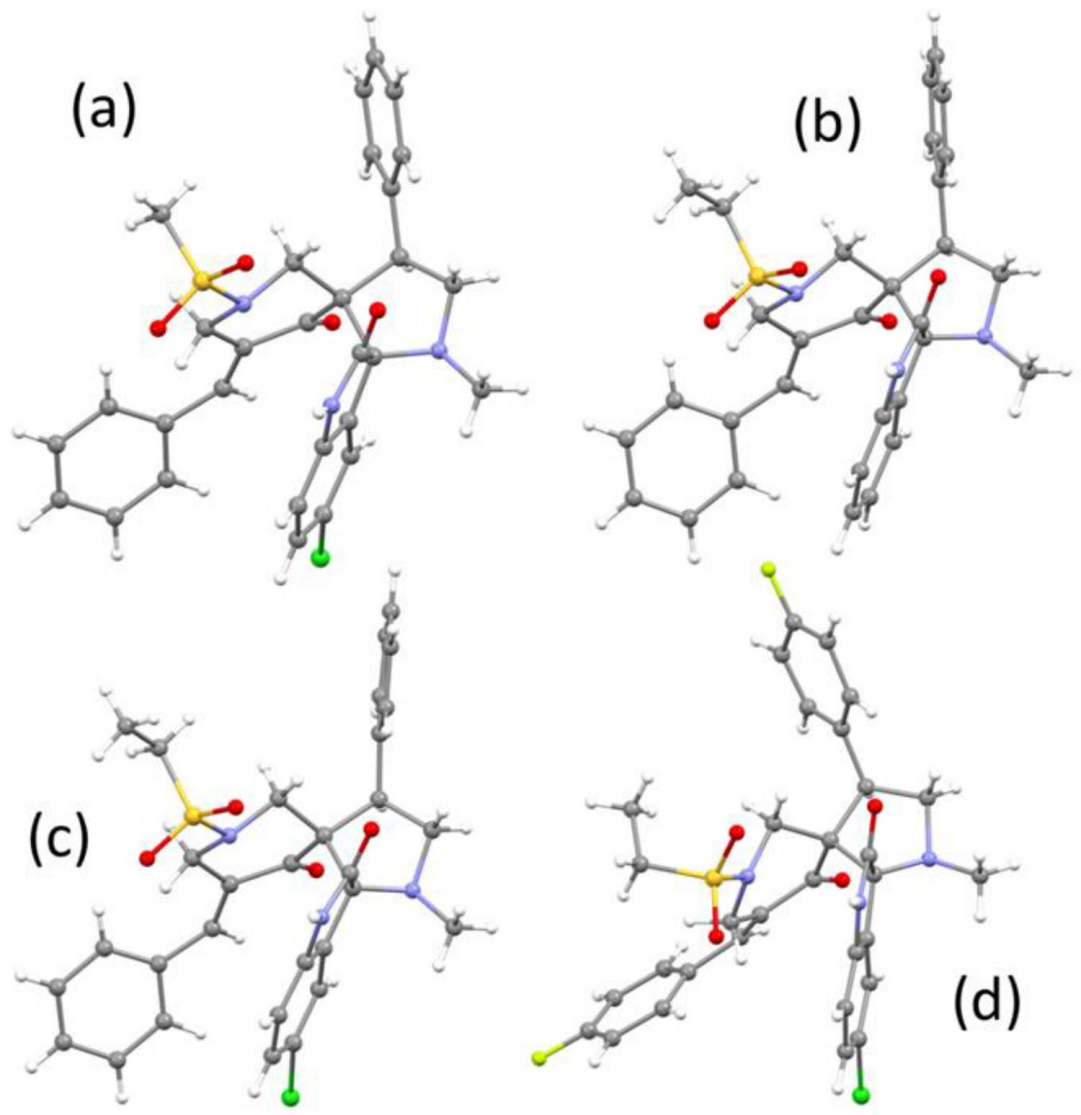
The molecular structures of (**a**) **6b**, (**b**) **6c** (**c**) **6d** and (**d**) **6h**.

Molecular structures of **6b**, **6c**, **6d** and **6h** are shown in Fig. 5 with structure determination presented in Supplementary Table S1. A possible factor in the ability of the molecules to interact with biological systems is molecular flexibility, in which the rotational freedom of the alkane-sulfonyl and phenyl groups would play a role. The geometry of the pyrrolidine-piperidine systems in all the crystals of **6b**, **6c** and **6d** is very similar, as indicated by the torsion angles (Supplementary Table S2). The molecule in the crystal structure of **6h** also has generally similar geometry but significant deviation, of over 20° from the nearest value for the other molecules, for angle 4–5–6–1. This indicates that some flexibility is possible for the pyrrolidine-piperidine ring system. It is notable that the crystal of **6h** contains a solvate molecule (ethanol).

### Biological studies

#### Antiproliferation properties

Antiproliferation properties of the targeted agents were investigated by the standard MTT technique against diverse human cancer cell lines [MCF7 (breast), HCT116 (colon), A431 (skin squamous) and PaCa2 (pancreatic)]^67^ (Table 1, Supplementary Figs. S48‒S51). 5-Fluorouracil (a clinically accessible drug for colon, breast and skin cancers)^68,69^ and sunitinib (an effective drug for gastrointestinal, renal and pancreatic cancers)^70,71^ were considered as standard references.

**Table 1:** Antiproliferation properties of the synthesized spiro-3-indolin-2-ones **6a‒o** and standard references (5-fluorouracil and sunitinib).

Entry	Compd.	IC_50_ (µM) ± SEM (SI)^a^				
		MCF7	HCT116	A431	PaCa-2	RPE1
1	**6a**	19.787 ± 0.99 (> 2.5)	15.957 ± 1.10 (> 3.1)	32.340 ± 1.14 (> 1.5)	48.404 ± 2.23 (> 1.0)	> 50.000 ± 1.09
2	**6b**	7.660 ± 0.68 (3.2)	6.915 ± 0.52 (3.6)	9.149 ± 0.70 (2.7)	20.638 ± 1.17 (1.2)	24.681 ± 1.26
3	**6c**	> 50.000 ± 1.94 (–)	6.125 ± 0.44 (> 8.2)	33.191 ± 0.91 (> 1.5)	> 50.000 ± 2.00 (–)	> 50.000 ± 2.00
4	**6d**	6.915 ± 0.55 (> 7.2)	5.181 ± 0.61 (> 9.7)	4.958 ± 0.25 (> 10.1)	13.085 ± 1.10 (> 3.8)	> 50.000 ± 1.85
5	**6e**	15.532 ± 0.76 (3.2)	9.894 ± 0.85 (5.0)	16.064 ± 0.99 (3.1)	39.894 ± 1.89 (1.2)	49.043 ± 1.11
6	**6f**	5.000 ± 0.39 (3.6)	5.431 ± 0.46 (3.3)	4.764 ± 0.37 (3.7)	11.702 ± 0.94 (1.5)	17.766 ± 0.87
7	**6g**	10.319 ± 0.86 (3.2)	4.944 ± 0.25 (6.8)	6.167 ± 0.44 (5.4)	28.404 ± 0.85 (1.2)	33.404 ± 1.22
8	**6h**	4.694 ± 0.44 (> 10.7)	4.597 ± 0.18 (> 10.9)	6.042 ± 0.26 (> 8.3)	14.043 ± 0.73 (> 3.6)	> 50.000 ± 2.38
9	**6i**	5.014 ± 0.29 (2.9)	5.472 ± 0.32 (2.7)	4.403 ± 0.49 (3.4)	9.043 ± 0.62 (1.6)	14.787 ± 1.57
10	**6j**	4.514 ± 0.39 (2.8)	4.722 ± 0.25 (2.6)	4.083 ± 0.21 (3.1)	8.830 ± 0.51 (1.4)	12.500 ± 0.86
11	**6k**	4.375 ± 0.26 (3.4)	4.167 ± 0.38 (3.5)	2.966 ± 0.29 (5.0)	8.830 ± 0.70 (1.7)	14.792 ± 0.99
12	**6l**	3.986 ± 0.31(> 12.5)	4.111 ± 0.41 (> 12.2)	3.694 ± 0.33 (> 13.5)	11.915 ± 0.83 (> 4.2)	> 50.000 ± 2.32
13	**6m**	3.597 ± 0.19 (4.1)	3.236 ± 0.27 (4.6)	2.434 ± 0.18 (6.1)	12.500 ± 0.67 (1.2)	14.894 ± 1.61
14	**6n**	40.213 ± 1.10 (> 1.2)	15.426 ± 0.52 (> 3.2)	34.894 ± 1.36 (> 1.4)	32.766 ± 1.21 (> 1.5)	> 50.000 ± 2.21
15	**6o**	48.936 ± 1.84 (> 1.0)	28.511 ± 0.75 (> 1.8)	45.417 ± 1.84 (> 1.1)	> 50.000 ± 2.31 (–)	> 50.000 ± 2.61
16	**5-Fluorouracil**	3.15 ± 0.44	20.43 ± 1.99	23.44 ± 2.09	–	–
17	**Sunitinib**	3.97 ± 0.32	9.67 ± 0.22	–	16.91 ± 0.95	–

^a^SI (selectivity index) = $\frac{{IC}_{50} \; of \;RPE1}{{IC}_{50} \; of \;cancer \;cell \;line}$

#### MCF7 cell line

Many of the synthesized spiro-3-indolin-2-ones reveal promising antiproliferation potency against MCF7 cancer cell line. Compound **6m** (R = 4-BrC_6_H_4_, R′ = Me, R″ = H) is the most effective agent with antiproliferation properties close to 5-fluorouracil and higher than sunitinib (IC_50_ = 3.597, 3.15, 3.97 µM for **6m**, 5-fluorouracil and sunitinib, respectively). Compound **6l** (R = 4-ClC_6_H_4_, R′ = Et, R″ = Cl) also shows comparable antiproliferation efficacy (IC_50_ = 3.986 µM). Compounds **6f**, **h‒k** display considerable potency as anti-MCF7 as well (IC_50_ = 4.375‒5.014 µM).

Some SARs (structure–activity relationships) can be assigned due to the observed antiproliferation properties. The chloro-substituted indolyl-containing heterocycles are more effective anti-MCF7 agents than the unsubstituted analogs (compound **6o** is an exception). The efficacy of halo-substituted phenyl containing compounds as anti-MCF7 is in the following order bromophenyl > chlorophenyl > fluorophenyl suggesting the inductive effect (‒I effect) of the halogen atom as a collaborative factor for antiproliferation properties. The higher the ‒I effect of the halogen atom, the lower antiproliferation properties against MCF7 as shown in compounds **6m**/**6i**/**6e** (IC_50_ = 3.597, 5.014, 15.532 µM, respectively), **6j**/**6f** (IC_50_ = 4.514, 5.000 µM, respectively), **6k**/**6g** (IC_50_ = 4.375, 10.319 µM, respectively) and **6l**/**6h** (IC_50_ = 3.986, 4.694 µM, respectively).

#### HCT116 cell line

Generally, all the synthesized agents (compound **6o** is an exception) reveal enhanced anti-HCT116 properties exceeding that of 5-fluorouracil (a potent drug against colon cancer). Compound **6m** (R = 4-BrC_6_H_4_, R′ = Me, R″ = H) heads the synthesized analogs with higher efficacy against HCT116 than those of the standard references used (IC_50_ = 3.236, 20.43, 9.67 µM for **6m**, 5-fluorouracil and sunitinib, respectively). Compounds **6g**,**h**,**j‒l** also show promising efficacies against HCT116 (IC_50_ = 4.111‒4.944 µM).

SARs inferred from the anti-HCT116 results are similar to those mentioned for the anti-MCF7 observations. The chloroindolinyl-containing heterocycles (compound **6o** is an exception) have higher anti-HCT116 properties than the unsubstituted derivatives. Additionally, the –I effect due to the halogen atom attached to the phenyl ring is an important parameter for anti-HCT116 properties. It is notable that the fluorophenyl-containing compounds have lower anti-HCT116 properties than chlorophenyl-containing analogues. However, the bromophenyl-containing analogue (**6m**) is the most effective/leading agent relative to the other halogenated phenyl-containing compounds. This SAR trend is supported by the antiproliferation observations for compounds **6m**/**6i**/**6e** (IC_50_ = 3.236, 5.472, 9.894 µM, respectively), **6j**/**6f** (IC_50_ = 4.722, 5.431 µM, respectively), **6k**/**6g** (IC_50_ = 4.167, 4.944 µM, respectively) and **6l**/**6h** (IC_50_ = 4.111, 4.597 µM, respectively).

#### A431 cell line

Compound **6m** is the most promising agent synthesized with efficacy about 9.6 times more than the standard reference (IC_50_ = 2.434, 23.44 µM for **6m** and 5-fluorouracil, respectively). Comparable potency was also displayed by compound **6k** (IC_50_ = 2.966 µM). Compounds **6d**,**f**,**i**,**j**,**l** additionally show promising efficacies (IC_50_ = 3.694‒4.958 µM).

The anti-A431 results support the SAR observation regarding the role of chlorine substitution to the indolinyl heterocycle in enhancing the antiproliferation properties relative to the unsubstituted analogs (compounds **6l** and **6o** are exceptions). The –I effect of the halogen atom attached to the phenyl group is also a contributory factor in the development of anti-A431 properties. Compounds with a fluorophenyl ring have lower anti-A431 proliferation values than the corresponding agents with a chlorophenyl ring. The bromophenyl-containing compound (**6m**) is superior to the other halogenophenyl-containing analogs. This is supported by the anti-A431 observations of compounds **6m**/**6i**/**6e** (IC_50_ = 2.434, 4.403, 16.064 µM, respectively), **6j**/**6f** (IC_50_ = 4.083, 4.764 µM, respectively), **6k**/**6g** (IC_50_ = 2.966, 6.167 µM, respectively) and **6l**/**6h** (IC_50_ = 3.694, 6.042 µM, respectively). The SARs are comparable to those previously discussed for anti-MCF7 and anti-HCT116 properties.

#### PaCa-2 cell line

Some of the synthesized spiro-3-indolin-2-ones show anti-PaCa-2 properties with efficacy higher than that of the reference standard used (sunitinib, a drug applicable for pancreatic cancer treatment). Compounds **6j** and **6k** are the most promising agents against PaCa-2 (IC_50_ = 8.830 µM for both **6j** and **6k**; compared to IC_50_ = 16.91 µM for sunitinib). Compounds **6f**,**i**,**l**,**m** also show promising anti-PaCa-2 properties (IC_50_ = 9.043‒12.500 µM).

SARs deduced from the anti-PaCa-2 properties indicate mainly the same parameters/controlling factors revealed by the previously mentioned cell lines. The chloro-substituted indolyl-containing heterocycles show higher anti-PaCa-2 efficacies than the unsubstituted analogs (compounds **6l** and **6o** are exceptions). The chlorophenyl-containing compounds have more enhanced anti-PaCa-2 properties than the fluorophenyl-containing analogs as shown in pairs **6i**/**6e** (IC_50_ = 9.043, 39.894 µM, respectively), **6j**/**6f** (IC_50_ = 8.830, 11.702 µM, respectively), **6k**/**6g** (IC_50_ = 8.830, 28.404 µM, respectively) and **6l**/**6h** (IC_50_ = 11.915, 14.043 µM, respectively).

#### RPE1 cell line

The safety of the synthesized agents against non-cancer/normal RPE1 (retinal pigment epithelium) cell line was assessed (Table 1, Supplementary Fig. S52). The SI (selectivity index) for the tested compounds against RPE1 cell line confirms the safety towards non-cancer cells. Compound **6m** (the most promising analog synthesized against MCF7, HCT116 and A431 cell lines) shows high SI values (SI = 4.1‒6.1). Compound **6k** (potent agent against PaCa2) reveals considerable SI value (SI = 1.7). Compound **6d** and **6h** which are also of high anti-PaCa2 show remarkable SI values (SI =  > 3.8, > 3.6, respectively).

### Cell cycle studies

Flow cytometry (FC) is a reliable technique for evaluation of cancer cell progression^72–74^. Illumination of the cells by lasers can identify the stained propidium iodide (PI) DNA cell content in stoichiometric population value. This is a commonly accessible methodology for studying cancer cell antiproliferation and cell cycle phase suppression^75^. Compounds **6l** and **6m** which are potent analogs against MCF7 (IC_50_ = 3.986, 3.597 µM) were considered for cell cycle studies by the standard PI-FC technique^76^ utilizing the IC_50_ values observed through MTT assay (Table 1, Supplementary Fig. S48).

**Figure 6:**
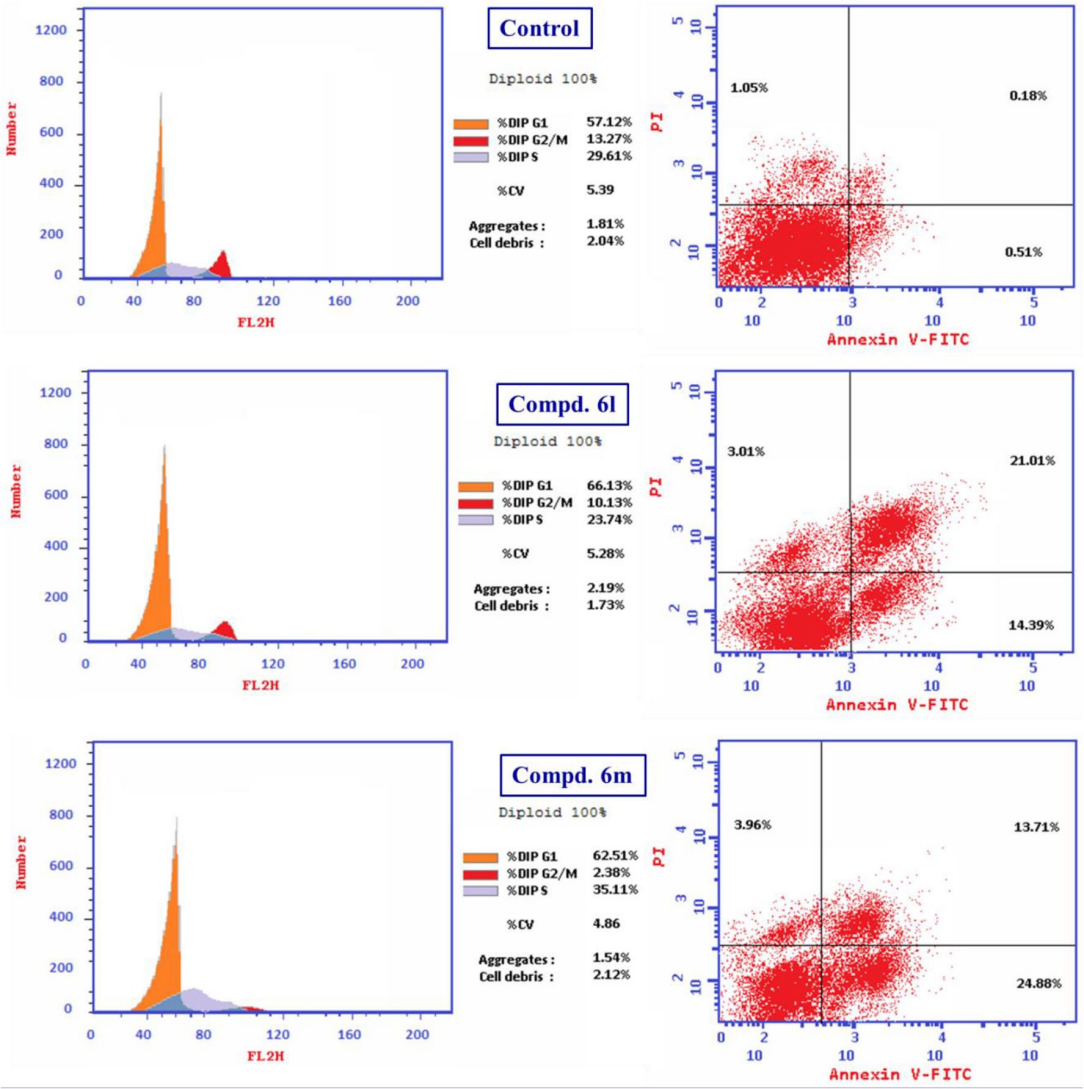
Cell cycle analysis of compounds **6l**, **6m** and control experiment for MCF7 (breast cancer cell line).

**Figure 7:**
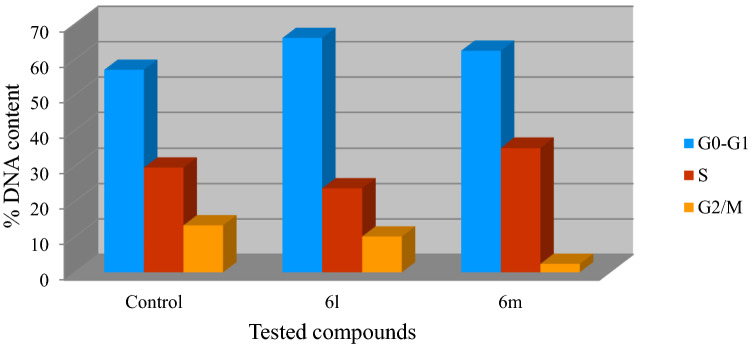
% DNA content of compounds **6l**, **6m** and control experiment for MCF7 (breast cancer cell line) at G0-G1, S and G2/M phases through PI-FC cell cycle studies.

It is noteworthy that the percentage (%) of DNA content for compound **6l** was higher in G1 phase than the control experiment (% DNA = 66.13, 57.12 for compound **6l** and control experiment at G1 phase, respectively). This is conclusive evidence for the ability of compound **6l** to suppress the tested cell line (MCF7) by arresting the cell cycle progress at G1 phase. Meanwhile, accumulation of % DNA content was observed by compound **6m** at both G1 and S phases relative to the control experiment (% DNA = 62.51, 35.11; 57.12, 29.61 for compound **6m** and control experiment at G1 and S phases, respectively). This is an indication for the ability of compound **6m** to affect the MCF7 cell cycle progression due to its antiproliferation properties by arresting it at G1/S phases. Decrease of G2/M phase by both the tested compounds relative to the control experiment also supports the ability of the agents for suppression cell cycle progress due to their antiproliferation properties (% DNA = 10.13, 2.38, 13.27 for compounds **6l**, **6m** and control experiment at G2/M phase, respectively) (Table 2, Figs. 6 and 7).

**Table 2:** % DNA cell distribution of compounds **6l**, **6m** and control experiment for MCF7 (breast cancer cell line) by PI-FC.

Entry	Compd.	% DNA content		
		G0–G1	S	G2/M
1	**Control**	57.12	29.61	13.27
2	**6l**	66.13	23.74	10.13
4	**6m**	62.51	35.11	2.38

**Figure 8:**
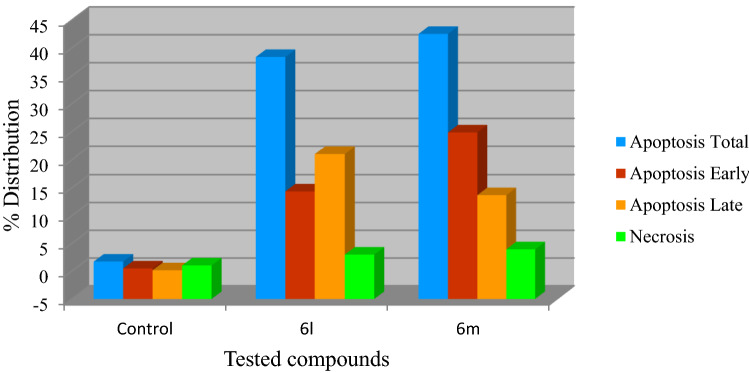
% Apoptosis and necrosis of compounds **6l**, **6m** and control experiment for MCF7 (breast cancer cell line).

It is also notable that both the tested compounds increase the apoptosis of the tested cancer cells^77^. Moreover, the total amount of apoptosis for the tested cell line is higher for compound **6m** than that for **6l** (total number of apoptosis = 38.41, 42.55 for compounds **6l** and **6m**, respectively). The late stage apoptosis of cells was observed in higher amount for compound **6l** than for **6m** (late stage apoptosis = 21.01, 13.71 for compounds **6l** and **6m**, respectively). On the other hand, necrosis observed for compound **6m** is relatively higher than that of compound **6l** (necrosis = 3.01, 3.96 for compounds **6l** and **6m**, respectively). In conclusion, it can be stated that both the tested compounds **6l** and **6m** are apoptosis and necrosis forming to the tested cell line due to their antiproliferation properties with compound **6m** seeming more effective than **6l**. These observations agree with the antiproliferation properties observed through MTT assay (Table 3, Fig. 8).

**Table 3:** % Apoptosis and necrosis of compounds **6l**, **6m** and control experiment for MCF7 (breast cancer cell line).

Entry	Compd.	Apoptosis (%)			Necrosis
		Total	Early	Late	
1	**Control**	1.74	0.51	0.18	1.05
2	**6l**	38.41	14.39	21.01	3.01
4	**6m**	42.55	24.88	13.71	3.96

### EGFR/VEGFR-2 inhibitory properties

EGFR (epidermal growth factor receptor) is a transmembrane protein tyrosine kinase involved in proliferation and differentiation in human cells (either normal or malignant). Increased EGFR activity due to overexpression is involved in many cancer types including non-small cell lung, breast, head and neck cancers. Therefore, agents targeting EGFR can serve as anticancer therapeutics with minimal off-target side effects^78–80^.

VEGFR (vascular endothelial growth factor receptor) is a cell surface tyrosine kinase receptor. Many members of the VEGFR family have been identified, indicating VEGFR-2 as the most important one. VEGFR-2 has attracted a lot of attention due to its important role against tumor-associated angiogenesis. Angiogenesis is the formation of new blood capillaries from pre-existing blood vessels. This is an essential process for many cellular functions such as proliferation, migration, and survival necessary for embryonic and adult development. Abnormal angiogenesis is associated with many diseases (such as inflammation, rheumatoid arthritis and cancer). This is the reason inhibition of VEGFR is a compelling approach to starve tumor cells and arrest solid tumor proliferation and metastasis^81–84^. Many small molecules have been discovered to possess VEGFR-2 inhibitory properties, including sunitinib^81^ (which is an indolyl scaffold with structural resemblance to the targeted synthesized agents).

The synthesized agents were considered for inhibition of EGFR and VEGFR-2 based on their solid tumor proliferation properties and chemical structural resemblance to sunitinib. The western blot technique was applied utilizing the IC_50_ observed for each respective agent synthesized during MTT assay^85,86^. Consideration of multi-targeted inhibitory properties investigation is based in the fact that cancer initiation and proliferation sometimes utilizes many receptors or signaling pathways. Clinical effectiveness reveals that single-target drugs usually suffer from cancer cell resistance due to heterogeneity of tumor cells. This is why multi-targeted agents are preferable over single-target or multi-component drug cocktails^87,88^.

The results for most of the synthesized agents (Table 4, Supplementary Fig. S53) reveal promising inhibitory properties against both enzymes utilized (EGFR and VEGFR-2). Compounds **6c** (R = Ph, R′ = Et, R″ = H) and **6n** (R = 3,4-(H_3_CO)_2_C_6_H_3_, R′ = Me, R″ = H) are the most effective of all the agents tested against EGFR, with high inhibitory properties (inhibition of EGFR = 69.6%). Compound **6f** (R = 4-FC_6_H_4_, R′ = Me, R″ = Cl) and **6i** (R = 4-ClC_6_H_4_, R′ = Me, R″ = H) also reveal comparable efficacies (% inhibition of EGFR = 69.3, 68.7 for **6f** and **6i**, respectively). SARs based on the inhibitory properties of the synthesized agents on EGFR show that the chloroindolyl-containing compounds are less potent than the unsubstituted analogs (compounds **6f** and **6h** are exceptions).

**Table 4:** Inhibitory properties of the synthesized spiro-3-indolin-2-ones **6a‒o** and standard reference (sunitinib) against EGFR and VEGFR-2.

Entry	Compd.	EGFR		VEGFR-2	
		RQ^a^	% Inhibition	RQ^a^	% Inhibition
1	Control	3.4461	–	3.2634	–
2	**6a**	1.1593	66.4	1.2773	60.9
3	**6b**	1.5804	54.1	1.16695	64.2
4	**6c**	1.0493	69.6	1.18043	63.8
5	**6d**	1.2774	62.9	1.18053	63.8
6	**6e**	1.147	66.7	1.15783	64.5
7	**6f**	1.05844	69.3	1.15279	64.7
8	**6g**	1.28032	62.8	1.18604	63.7
9	**6h**	1.12953	67.2	1.10352	66.2
10	**6i**	1.07732	68.7	1.1684	64.2
11	**6j**	1.2634	63.3	1.3745	57.9
12	**6k**	1.16345	66.2	1.10463	66.2
13	**6l**	1.18045	65.7	1.29942	60.2
14	**6m**	1.1845	65.6	1.26354	61.3
15	**6n**	1.04732	69.6	1.16694	64.2
16	**6o**	1.17467	65.9	1.2473	61.8
17	**Sunitinib**	0.6424	81.4	0.8265	74.7

^a^RQ is the relative quantification.

Compounds **6h** (R = 4-FC_6_H_4_, R′ = Et, R″ = Cl) and **6k** (R = 4-ClC_6_H_4_, R′ = Et, R″ = H) are the most effective agents against VEGFR-2 showing promising inhibitory properties (inhibition of VEGFR-2 = 66.2%). Compound **6e** (R = 4-FC_6_H_4_, R′ = Me, R″ = H) also has comparable efficacy (inhibition of VEGFR-2 = 64.5%). SARs based on the observed inhibition of VEGFR-2 supports the observation that the fluorophenyl-containing spiro-3-indolin-2-ones have higher efficacies than unsubstituted phenyl-containing analogs [compound **6c** is an exception with nearly the same efficacy to that of **6g** (% inhibition of VEGFR-2 = 63.8, 63.7 for **6c** and **6g**, respectively)] as shown by pairs **6a**/**6e** (% inhibition of VEGFR-2 = 60.9, 64.5, respectively), **6b**/**6f** (% inhibition of VEGFR-2 = 64.2, 64.7, respectively) and **6d**/**6h** (% inhibition of VEGFR-2 = 63.8, 66.2, respectively).

Slight variations in the enzymatic inhibitory results relative to the antiproliferations properties of the tested compounds can be rationalize by the difference in experimental techniques.

### Anti-SARS-CoV-2 properties

**Figure 9:**
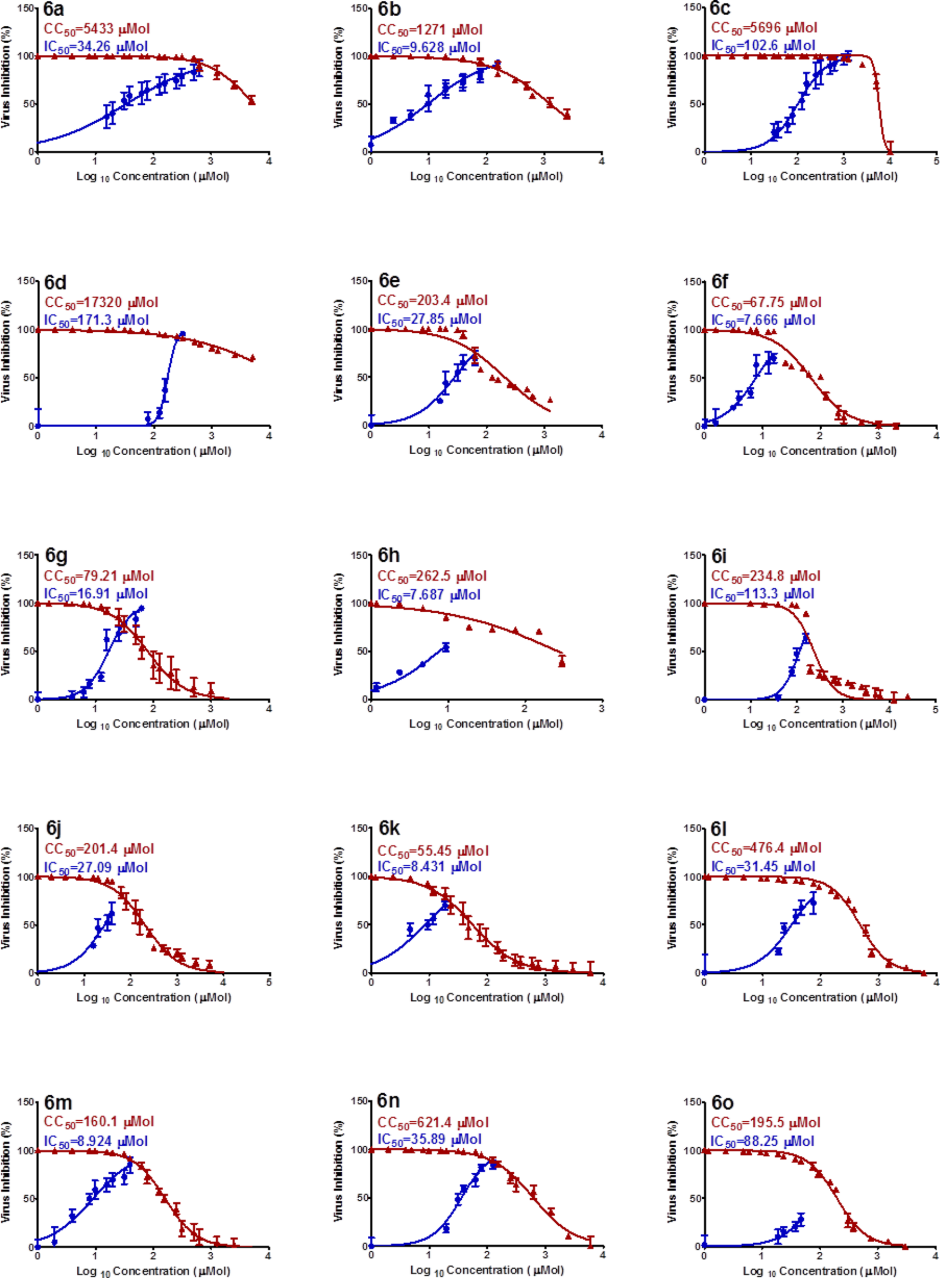
Dose–response curves for the synthesized agents against SARS-CoV-2.

The Vero-E6 cell viral infection model/technique was undertaken to determine the anti-SARS-CoV-2 inhibitory properties of the synthesized spiro-3-indolin-2-ones **6a‒o**^44^. Favipiravir^89^, Hydroxychloroquine and Chloroquine^51^ were considered as standard references (Table 5, Fig. 9). It is apparent from the results that many of the synthesized agents show efficacy against SARS-CoV-2 with potency higher than the standard references. Compound **6f** (R = 4-FC_6_H_4_, R′ = Me, R″ = Cl) is the most promising of all the synthesized spiro-3-indolin-2-ones (IC_50_ = 7.666 µM) with about 3.3 and 4.8 times more potency than the standard references, chloroquine and hydroxychloroquine (IC_50_ = 24.98, 36.92 µM, respectively). Compound **6h** (R = 4-FC_6_H_4_, R′ = Et, R″ = Cl; IC_50_ = 7.687 µM) reveals an efficacy close to that of **6f.** Compounds **6b**, **6k** and **6m** are also promising anti-SARS-CoV-2 active agents (IC_50_ = 8.431‒9.628 µM).

**Table 5:** Antiviral (SARS-CoV-2) properties of the synthesized spiro-3-indolin-2-ones **6a‒o** and standard references.

Entry	Compd	IC_50_ (*µ*M)	CC_50_ (*µ*M)	SI^a^
1	**6a**	34.26	5433	158.6
2	**6b**	9.628	1271	132.0
3	**6c**	102.6	5696	55.5
4	**6d**	171.3	17,320	101.1
5	**6e**	27.85	203.4	7.3
6	**6f**	7.666	67.75	8.8
7	**6g**	16.91	79.21	4.7
8	**6h**	7.687	262.5	34.1
9	**6i**	113.3	234.8	2.1
10	**6j**	27.09	201.4	7.4
11	**6k**	8.431	55.45	6.6
12	**6l**	31.45	476.4	15.1
13	**6m**	8.924	160.1	17.9
14	**6n**	35.89	621.4	17.3
15	**6o**	88.25	195.5	2.2
16	**Favipiravir**^**b**^	1382	5262	3.8
17	**Hydroxychloroquine**^**c**^	36.92	356.4	9.7
18	**Chloroquine**^**c**^	24.98	377.7	15.1

^a^SI = CC_50_/IC_50_.

^b^Ref.^89^.

^c^Ref.^51^.

Evident SARs from the results indicate that the chloroindolyl-containing compounds have higher efficacy against SARS-CoV-2 than the unsubstituted analogs (compounds **6d**, **6l** and **6o** are exceptions). The ethylpiperidone-containing heterocycles (compounds **6g** and **6k** are exceptions) have lower potency against SARS-CoV-2 than the methyl-containing analogs as shown by pairs **6a**/**6c**, **6b**/**6d**, **6f**/**6h** and **6j**/**6l** (IC_50_ = 34.26/102.6, 9.628/171.3, 7.666/7.687, 27.09/31.45 µM, respectively). Additionally, the fluorophenyl-containing compounds display higher anti-SARS-CoV-2 properties than the unsubstituted phenyl-containing analogs as shown by pairs **6a**/**6e**, **6b**/**6f**, **6c**/**6g** and **6d**/**6h** (IC_50_ = 34.26/27.85, 9.628/7.666, 102.6/16.91, 171.3/7.687 µM, respectively). Acceptable safety indexes (SI) were observed for the synthesized agents. The chloroindolyl-containing heterocycles have higher SI than the unsubstituted indolyl-containing analogs (compounds **6b** and **6o** are exceptions).

### Cholinesterase inhibitory properties

Alzheimer’s disease is a progressive neurodegenerative illness and causes the majority of dementia cases in the elderly. Progression of the disease affects the areas of the brain responsible for memory, thoughts and language skills of the patient. In the advanced stage of the disease, the patient is rendered unable to meet their basic needs of life. Most of the medications available act to slow the progress of the symptoms. Restoring the level of cholinesterases [acetylcholinesterase (AChE), butyrylcholinesterase (BChE)] is one of the most important approaches in Alzheimer’s disease treatment^90–92^. Acetylcholine is a brain neurotransmitter with a major role in the maintenance of memory and consciousness^93^. AChE is the enzyme that hydrolyses acetylcholine. BChE can also regulate its level. Therefore, inhibition of both AChE and BChE is one of the main approaches for treating Alzheimer’s^94,95^. The synthesized agents in the current study were assessed for AChE and BChE inhibitory properties based on the fact that many indolyl-containing compounds inhibit cholinesterases^96–99^. Numerous reports have also referred to the cholinesterase inhibitory properties of spiro-indole-containing compounds^25–28,100^.

The results (Table 6) reveal that some of the synthesized spiro-3-indolin-2-ones have promising inhibitory properties against both AChE and BChE. However, none of the synthesized agents shows potency comparable to the standard reference used (donepezil). Compound **6g** (R = 4-FC_6_H_4_, R′ = Et, R″ = H) is the most potent of the synthesized agents with the highest inhibitory properties against both AChE and BChE (IC_50_ = 2.46, 3.22 μM, respectively). Compound **6f** (R = 4-FC_6_H_4_, R′ = Me, R″ = Cl) also exhibits promising inhibitory efficacies against both AChE and BChE (IC_50_ = 3.89, 3.75 μM, respectively). Additionally, compounds **6n** and **6h** have considerable inhibitory potencies against both AChE and BChE (IC_50_ = 5.40, 6.33; 6.30, 8.07 μM for **6n** and **6h** respectively).

**Table 6:** AChE and BChE inhibition properties of the synthesized spiro-3-indolin-2-ones **6a‒o** and standard reference (donepezil).

Entry	Compd	AChE (IC_50_, μM) ± SD	BChE (IC_50_, μM) ± SD	SI^a^
1	**6a**	22.19 ± 3.35	19.75 ± 2.18	1.1
2	**6b**	27.65 ± 5.45	29.23 ± 3.62	0.9
3	**6c**	77.95 ± 3.26	65.41 ± 2.42	1.2
4	**6d**	41.01 ± 7.03	34.38 ± 2.00	1.2
5	**6e**	15.12 ± 5.76	22.17 ± 4.54	0.7
6	**6f**	3.89 ± 1.66	3.75 ± 0.71	1.0
7	**6g**	2.46 ± 0.99	3.22 ± 0.92	0.8
8	**6h**	6.30 ± 0.97	8.07 ± 1.47	0.8
9	**6i**	18.21 ± 1.48	13.61 ± 2.12	1.3
10	**6j**	42.84 ± 4.61	22.27 ± 3.33	1.9
11	**6k**	32.31 ± 4.78	19.62 ± 2.10	1.6
12	**6l**	23.59 ± 7.73	11.78 ± 0.95	2.0
13	**6m**	29.71 ± 4.05	27.93 ± 2.96	1.1
14	**6n**	5.40 ± 0.94	6.33 ± 0.85	0.9
15	**6o**	27.05 ± 6.70	24.93 ± 4.60	1.1
16	**Donepezil**	0.59 ± 0.083	0.77 ± 0.01	0.8

^a^SI = IC_50_ (AChE)/IC_50_ (BChE).

It is evident that the fluorophenyl-containing compounds are more inhibitory against AChE than the unsubstituted phenyl-containing analogs as shown by pairs **6e**/**6a**, **6f**/**6b**, **6g**/**6c** and **6h**/**6d** (IC_50_ = 15.12/22.19, 3.89/27.65, 2.46/77.95, 6.30/41.01 μM, respectively). The same observation is also made against BChE with compound **6e** is an exception (IC_50_ = 3.75/29.23, 3.22/65.41, 8.07/34.38 μM for **6f**/**6b**, **6g**/**6c** and **6h**/**6d**, against BChE respectively).

The SI (selectivity index due to IC_50_ against AChE relative to the IC_50_ against BChE) of compound **6e** is higher than that of the standard reference used (SI = 0.7, 0.8 for compound **6e** and donepezil, respectively). This is due to its selective inhibitory properties against BChE relative to the AChE (IC_50_ = 15.12, 22.17 μM against AChE and BChE, respectively). It is also noted that the SI values of compounds **6g** and **6h** are similar to that of the standard reference (SI = 0.8). Compounds **6b** and **6n** have SI comparable to that of the standard reference (SI = 0.9 for compounds **6b** and **6n**).

### Molecular modeling studies

Molecular modeling techniques are useful tools in medicinal chemical studies. Various techniques can be utilized in predicating new hits/leads, identifying the parameters necessary for bio-properties and understanding the exhibited bio-observations^25,101^. QSAR (quantitative structure–activity relationship) is one of the available approaches capable of generating mathematical models for connecting the bio-properties with physico-chemical (descriptor) parameters. Three main steps of QSAR modeling are; chemical structure optimization, descriptor calculation and validated modeling identification^102^. The current studies were undertaken by the CODESSA-Pro software^25^.

### MCF7 QSAR model

The robust two-descriptor QSAR model (*R*^2^ = 0.977, *R*^2^cvOO = 0.960, *R*^2^cvMO = 0.956) associates the antitumor properties of the synthesized spiro-3-indolin-2-ones with a wide range of bio-properties (IC_50-observed_ = 3.597‒48.936 µM, IC_50-predicted_ = 0.922‒46.734 µM) (Supplementary Tables S3–S5, Fig. S54). The maximum nucleophilic reactivity index for atom O (semi-empirical descriptor) has higher criterion (*t* = 21.368) and coefficient (5164.31) values than the other model’s descriptors. Due to this, the compound with high mathematical descriptor value estimates low antiproliferation properties as shown for compounds **6j** and **6o** (descriptor value = 0.00385, 0.01224 corresponding to estimated IC_50_ = 0.922, 46.734 µM, respectively). This descriptor also explains the low antiproliferation properties of methoxy group-containing compounds against MCF7 cell line relative to the other synthesized analogs. Fukui atomic nucleophilic reactivity index can be calculated by Eq. (1).^103^

1 $${N}_{A}=\sum_{i\in A}{C}_{iHOMO}^{2}/(1-{\varepsilon }_{HOMO})$$

where the ${\varepsilon }_{HOMO}$, ${C}_{iHOMO}$ are the highest occupied molecular orbital energy and its coefficient, respectively.

The square root of partial surface area for atom C is a charge-related descriptor also with a high coefficient value = 936.267. Again, the compound with low mathematical coefficient value reveals potent antiproliferation properties against the tested cell lines. This is obvious in compounds **6a** and **6l** (descriptor value = 0.07704, 0.06474 corresponding to estimated IC_50_ = 19.024, 1.387 µM, respectively). Partial positively/negatively charged surface area can be calculated by Eq. (2).^103^

2 $$PPSA1=\sum_{A}{S}_{A}$$

where ${S}_{A}$ stands either for the positively or negatively charged solvent accessible atomic surface area.

### HCT116 QSAR model

The two-descriptor QSAR model with good coefficient value (*R*^2^ = 0.834, *R*^2^cvOO = 0.709, *R*^2^cvMO = 0.742) expresses the antiproliferation properties of the synthesized agents against the HCT116 cell line. The model covers a wide range of biological properties (IC_50-observed_ = 3.236‒28.511 µM, IC_50-predicted_ = 2.153‒26.176 µM) (Supplementary Tables S6–S8, Fig. S55). Maximum n–n repulsion for bond C-N (*t* = 6.194) is a semi-empirical descriptor which contributes with a positive sign in the QSAR model. This explains the low efficacy of the agent with high mathematical descriptor value as shown by compound **6o** relative to **6j** (descriptor value = 167.7668, 168.4408 corresponding to estimated IC_50_ = 2.153, 26.176 µM for compounds **6j** and **6o**, respectively). The electron–electron repulsion between two different atoms can be calculated by Eq. (3).^103^

3 $${E}_{ee}\left(AB\right)=\sum_{\mu ,\nu \in A}\sum_{\lambda ,\sigma \in B}{P}_{\mu \nu }{P}_{\lambda \sigma }\langle \mu \nu |\lambda \sigma \rangle$$

where *A* and *B* stand for two different atoms. ${P}_{\mu \nu }\mathrm{and} {P}_{\lambda \sigma }$ stand for the density matrix over the atomic basis $\left\{\mu \nu \lambda \sigma \right\}$. $\langle \mu \nu |\lambda \sigma \rangle$ stands for the electron repulsion integrals for the atomic basis $\left\{\mu \nu \lambda \sigma \right\}$.

HA dependent HDCA-2 is a charge-related descriptor with a high coefficient value (30.3734). This also explains the low efficacy of the compounds possessing high mathematical descriptor values as revealed in compound **6n** relative to **6k** (descriptor value = 0.47269, 0.88784 corresponding to estimated IC_50_ = 2.688, 15.485 µM for compounds **6k** and **6n**, respectively). The area weighted surface charge of hydrogen bonding donor atoms HDCA2 can be calculated by Eq. (4).^103^

4 $$HDCA2=\sum_{D}\frac{{q}_{D}\sqrt{{S}_{D}}}{\sqrt{{S}_{tot}}} D\in {H}_{H-donor}$$

where ${S}_{D}$ stands for the solvent accessible surface area for the hydrogen bonding donor of the hydrogen atoms selected by the threshold charge. The ${q}_{D}$ is partial charge on the hydrogen bonding donor of the hydrogen atoms selected by the threshold charge. ${S}_{tot}$ is total solvent accessible molecular surface area.

### A431 QSAR model

A validated two-descriptor 2D-QSAR model (*R*^2^ = 0.898, *R*^2^cvOO = 0.800, *R*^2^cvMO = 0.816) describes the antiproliferation properties of the synthesized spiro-3-indolin-2-ones (Supplementary Tables S9–S11, Fig. S56). A wide range of bio-properties (IC_50-observed_ = 2.434‒45.417 µM, IC_50-predicted_ = 3.027‒62.035 µM) is covered by the model. FPSA2 fractional PPSA is a charge-related descriptor with high criterion (*t* = 9.256) and coefficient (2.06853) values. This explains the high efficacy antiproliferation properties of compound **6l** over **6n** (descriptor value = 0.56735, 1.14432 corresponding to estimated IC_50_ = 3.027, 62.035 µM, respectively). The fractional charge partial positive surface area can be calculated by Eq. (5).^103^

5 $$FPSA2=PPSA2/TMSA$$

where the $PPSA2$ stands for the total positively partial charged molecular surface area. $TMSA$ stands for the total surface area of the molecule.

Average information content is a topological descriptor with a negative coefficient value (− 2.14585). For this reason, the synthesized agent with a high mathematical value represents a potent antiproliferation agent as shown by compounds **6a** and **6h** (descriptor value = 4.49045, 5.02123 corresponding to estimated IC_50_ = 28.534, 3.930 µM, respectively). The mean information content index can be calculated by Eq. (6).^103^

6 $${k}_{IC}=-\sum_{i=1}^{k}\frac{{n}_{i}}{n}{log}_{2}\frac{{n}_{i}}{n}$$

where ${n}_{i}$ stands for the atom number in the ${i}$th class. The *n* is the total number of molecular atoms. *k* is the atomic layer numbers in the coordination sphere surrounding a specific atom.

### PaCa-2 QSAR model

The two-descriptor QSAR model describes the antiproliferation properties of the synthesized spiro-3-indolin-2-ones against PaCa-2 cell line in an accurate mode (*R*^2^ = 0.9573, *R*^2^cvOO = 0.938, *R*^2^cvMO = 0.944). The model covers a wide range of bio-properties including potent and mild efficacies (IC_50-observed_ = 8.83‒48.404 µM, IC_50-predicted_ = 7.488‒47.054 µM) (Supplementary Tables S12–S14, Fig. S57). Rotational entropy is a thermodynamic descriptor with a negative coefficient (− 9.48172). This explains the high antiproliferation efficacy of an agent with high a mathematical descriptor value as exhibited in compounds **6l** over **6a** (descriptor value = 37.155, 38.725 corresponding to estimated IC_50_ = 47.054, 7.824 µM for compounds **6a** and **6l**, respectively). The rotational entropy of a molecule can be calculated by Eq. (7).^103^

7 $${S}_{rot}=Nkln\left[\frac{{\pi }^{1/2}}{\sigma }\prod_{j-1}^{3}{\left(\frac{8{\pi }^{2}{I}_{j}kT}{{h}^{2}}\right)}^{1/2}\right]$$

where ${I}_{j}$ stands for the principal moment of molecular inertia. The $\sigma$ stands for molecular symmetry number with, $h and k$ standing for Planck’s and Boltzmann’s constants. $T$ is the absolute temperature (K).

Maximum population of an electronic atomic orbital is a semi-empirical descriptor with a negative coefficient value (− 510.133). Therefore, a compound with a low mathematical value indicates low antiproliferation properties as shown in compound **6n** over **6j** (descriptor value = 1.98534, 1.93405 corresponding to estimated IC_50_ = 7.488, 33.729 µM for compounds **6j** and **6n**, respectively).

### SARS-CoV-2 QSAR model

The three descriptor QSAR model optimizes the anti-SARS-CoV-2 properties of the tested compounds (*R*^2^ = 0.917, *R*^2^cvOO = 0.863, *R*^2^cvMO = 0.883). The model covers a wide range of bio-properties (IC_50-observed_ = 7.666‒171.3 µM, IC_50-predicted_ = 7.669‒108.928 µM), including potent, mild and weak active agents (Supplementary Tables S15–S17, Fig. S58). LUMO + 1 energy is a semi-empirical descriptor with the highest coefficient value (2.66577) of the model’s descriptors. The compound with a high mathematical descriptor value has low potency as seen in compounds **6c** and **6m** (descriptor value = − 0.412, − 0.605 corresponding to estimated IC_50_ = 69.062, 8.612 µM, respectively). The LUMO (lowest unoccupied molecular orbital) energy can be calculated by Eq. (8).^103^

8 $${\varepsilon }_{LUMO}=\langle {\phi }_{LUMO}|\widehat{F}|{\phi }_{LUMO}\rangle$$

where ${\phi }_{LUMO}$ stands for the lowest unoccupied molecular orbital. $\widehat{F}$ stands for the Fock operator.

Maximum electrophilic reactivity index for atom O is also a semi-empirical descriptor with a negative sign coefficient value (− 120.804). This explains the weak anti-SARS-CoV-2 properties of compound **6a** relative to **6b** (descriptor value = 0.0117, 0.009 corresponding to estimated IC_50_ = 27.990, 9.632 µM, respectively). Equation (1) can calculate the descriptor value.

Minimum atomic state energy for atom N is a topological descriptor with a negative coefficient value (-7.98889). This explains the weak anti-SARS-CoV-2 activity of compound **6o** relative to **6m** (descriptor value = 184.7028, 184.5203 corresponding to estimated IC_50_ = 8.612, 108.928 µM, respectively).

The most appropriate QSAR validation technique is internal validation due to the short data set utilized. Statistical validation including, the standard division and Fisher criteria support the accuracy of the QSAR models. The comparative values of the model’s coefficient (*R*^2^) to their leave-one-out (*R*^2^cvOO) and leave-many-out (*R*^2^cvMO) coefficient values also validate the optimized QSAR models. The comparative predicted properties due to the QSAR models relative to the experimentally observed values, especially for the high potent analogs, also support the molecular models which can be considered in a future study for assigning higher potent hits/leads.

## Conclusion

In conclusion, the targeted (*E*)-1″-(alkanesulfonyl)-4′-aryl-5″-arylidene-1′-methyl-dispiro[indoline-3,2′-pyrrolidine-3′,3″-piperidine]-2,4″-diones **6a‒o** were regioselectively synthesized through multi-component dipolar cycloaddition reaction of 1-(alkylsulfonyl)-3,5-bis(ylidene)-piperidin-4-ones **3a‒h** and azomethine ylide. Single crystal X-ray studies (**6b‒d,h**) confirmed the structure. Some of the synthesized 3-spiro-indolin-2-ones reveal potent antiproliferation properties against diverse human cancer cells (MCF7, HCT116, A431 and PaCa2) but are safe towards normal (RPE1) cell line. Compound **6m** is the most potent agent synthesized against the tested cancer cells with comparable efficacies to those of 5-fluorouracil and sunitinib (standard references). Cell cycle studies of representative examples (**6l** and **6m**) confirm the antiproliferation properties revealed by the MTT technique and exhibit that they are apoptosis and necrosis forming. The antiproliferative agents synthesized can be considered multi-targeted inhibitors due to their properties against EGFR and VEGFR-2. Some of the synthesized 3-spiro-indolin-2-ones show promising anti-SARS-CoV-2 properties. Compound **6f** is the most potent (about 3.3 and 4.8 times the efficacy of the standard references chloroquine and hydroxychloroquine, respectively). Additionally, some of the synthesized spiro-3-indolin-2-ones reveal promising inhibitory properties against both AChE and BChE. QSAR models explained the diverse biological properties that can be considered for predicting promising hits/leads in future studies.

## Experimental

### Chemistry

Melting points were determined on a capillary point apparatus (Stuart SMP3) equipped with a digital thermometer. IR spectra (KBr) were recorded on a Shimadzu FT-IR 8400S spectrophotometer. Reactions were monitored using thin layer chromatography (TLC) on 0.2 mm silica gel F254 plates (Merck) utilizing various solvents for elution. The chemical structures of the synthesized compounds were characterized by nuclear magnetic resonance spectra (^1^H-NMR, ^13^C-NMR) and determined on a Bruker NMR spectrometer (500 MHz, 125 MHz for ^1^H and ^13^C, respectively). ^13^C-NMR spectra are fully decoupled. Chemical shifts were reported in parts per million (ppm) using the deuterated solvent peak or tetramethylsilane as an internal standard.

### Synthesis of spiro-3-indolin-2-ones **6a‒o** (general procedure)

A mixture of equimolar amounts of the appropriate 1-alkylsulfonyl-3,5-bis(ylidene)-piperidin-4-ones **3a‒h**^66^ (2.5 mmol) and the corresponding isatin **4a,b** with sarcosine **5** in ethanol (20 ml) was boiled under reflux for the appropriate time. The separated solid upon refluxing was collected and crystallized from a suitable solvent affording the corresponding **6a‒c**,**e‒g**,**i**,**m‒o**. For the remaining synthesized agents, the clear reaction mixture was stored at room temperature (20‒25 °C) overnight. The separated solid was collected and crystallized from a suitable solvent affording **6d**,**h**,**j-l**.

#### (*E*)-5″-Benzylidene-1′-methyl-1″-(methylsulfonyl)-4′-phenyldispiro[indoline-3,2′-pyrrolidine-3′,3″-piperidine]-2,4″-dione (**6a**)

Obtained from the reaction of **3a**, **4a** and **5**, reaction time 4 h as colorless microcrystals from n-butanol, with mp 232‒234 °C and yield 83% (1.1 g). IR: *ν*_max_/cm^−1^ 3186, 1705, 1678, 1615, 1593. ^1^H-NMR (DMSO-*d*_*6*_) *δ* (ppm): 1.96 (s, 3H, NCH_3_), 2.25 (d, *J* = 12.7 Hz, 1H, upfield H of piperidinyl H_2_C-2″), 2.65 (s, 3H, SCH_3_), 3.37 (br m, 1H, upfield H of pyrrolidinyl H_2_C-5′), 3.54 (d, *J* = 14.9 Hz, 1H, upfield H of piperidinyl H_2_C-6″), 3.84 (t, *J* = 9.2 Hz, 1H, downfield H of pyrrolidinyl H_2_C-5′), 3.93–3.98 (m, 2H, downfield H of piperidinyl H_2_C-2″ + downfield H of piperidinyl H_2_C-6″), 4.71 (t, *J* = 8.7 Hz, 1H, pyrrolidinyl H-4′), 6.69 (d, *J* = 7.4 Hz, 1H, arom. H), 6.86 (br s, 2H, arom. H), 7.09 (br s, 1H, arom. H), 7.23–7.51 (m, 11H, 10 arom. H + olefinic CH), 10.58 (s, 1H, NH). ^13^C-NMR (DMSO-*d*_*6*_) *δ* (ppm): 33.6, 33.7 (NCH_3_, SCH_3_), 45.7 (pyrrolidinyl HC-4′), 46.5 (piperidinyl H_2_C-6″), 47.9 (piperidinyl H_2_C-2″), 57.3 (pyrrolidinyl H_2_C-5′), 61.2 [spiro-C-3′ (C-3″)], 75.3 [spiro-C-3 (C-2′)], 109.1, 120.7, 124.9, 126.8, 127.1, 128.3, 128.6, 129.1, 129.48, 129.54, 129.8, 130.1, 133.8, 137.7, 138.9, 143.6 (arom. C + olefinic C), 175.1, 195.7 (C=O). Anal. Calcd. for C_30_H_29_N_3_O_4_S (527.64): C, 68.29; H, 5.54; N, 7.96. Found: C, 68.46; H, 5.81; N, 8.30.

#### (*E*)-5″-Benzylidene-5-chloro-1′-methyl-1″-(methylsulfonyl)-4′-phenyldispiro[indoline-3,2′-pyrrolidine-3′,3″-piperidine]-2,4″-dione (**6b**)

Obtained from the reaction of **3a**, **4b** and **5**, reaction time 7 h as colorless microcrystals from methanol, with mp 230‒232 °C and yield 90% (1.27 g). IR: *ν*_max_/cm^−1^ 3167, 1710, 1682, 1620, 1593. ^1^H-NMR (DMSO-*d*_*6*_) *δ* (ppm): 1.97 (s, 3H, NCH_3_), 2.29 (d, *J* = 12.8 Hz, 1H, upfield H of piperidinyl H_2_C-2″), 2.66 (s, 3H, SCH_3_), 3.35 (t, *J* = 9.0 Hz, 1H, upfield H of pyrrolidinyl H_2_C-5′), 3.71 (d, *J* = 14.9 Hz, 1H, upfield H of piperidinyl H_2_C-6″), 3.81 (t, *J* = 9.5 Hz, 1H, downfield H of pyrrolidinyl H_2_C-5′), 3.98 (t, *J* = 13.4 Hz, 2H, downfield H of piperidinyl H_2_C-2″ + downfield H of piperidinyl H_2_C-6″), 4.68 (t, *J* = 9.0 Hz, 1H, pyrrolidinyl H-4′), 6.69 (d, *J* = 8.3 Hz, 1H, arom. H), 6.78 (d, *J* = 2.0 Hz, 1H, arom. H), 7.15 (dd, *J* = 2.1, 8.3 Hz, 1H, arom. H), 7.28–7.52 (m, 11H, 10 arom. H + olefinic CH), 10.74 (s, 1H, NH). ^13^C-NMR (DMSO-*d*_*6*_) *δ* (ppm): 33.4, 33.8 (NCH_3_, SCH_3_), 45.9 (pyrrolidinyl HC-4′), 47.0 (piperidinyl H_2_C-6″), 48.3 (piperidinyl H_2_C-2″), 57.5 (pyrrolidinyl H_2_C-5′), 61.5 [spiro-C-3′ (C-3″)], 75.4 [spiro-C-3 (C-2′)], 110.6, 124.9, 126.7, 127.15, 127.19, 128.4, 128.7, 128.9, 129.7, 129.8, 130.1, 133.7, 137.5, 139.0, 142.5 (arom. C + olefinic C), 174.7, 195.6 (C=O). Anal. Calcd. for C_30_H_28_ClN_3_O_4_S (562.08): C, 64.11; H, 5.02; N, 7.48. Found: C, 64.32; H, 5.21; N, 7.63.

#### (*E*)-5″-Benzylidene-1″-(ethylsulfonyl)-1′-methyl-4′-phenyldispiro[indoline-3,2′-pyrrolidine-3′,3″-piperidine]-2,4″-dione (**6c**)

Obtained from the reaction of **3b**, **4a** and **5**, reaction time 4 h as yellow microcrystals from n-butanol, with mp 232‒234 °C and yield 88% (1.19 g). IR: *ν*_max_/cm^−1^ 3163, 1713, 1678, 1616, 1597. ^1^H-NMR (DMSO-*d*_*6*_) *δ* (ppm): 0.97 (t, *J* = 7.4 Hz, 3H, CH_3_CH_2_S), 1.97 (s, 3H, NCH_3_), 2.34 (d, *J* = 12.9 Hz, 1H, upfield H of piperidinyl H_2_C-2″), 2.74 (sextet, *J* = 7.3, 14.6 Hz, 1H, upfield H of CH_3_CH_2_S), 2.89 (sextet, *J* = 7.4, 14.7 Hz, 1H, downfield H of CH_3_CH_2_S), 3.33 (br s, 1H, upfield H of pyrrolidinyl H_2_C-5′), 3.54 (d, *J* = 15.0 Hz, 1H, upfield H of piperidinyl H_2_C-6″), 3.84 (t, *J* = 9.4 Hz, 1H, downfield H of pyrrolidinyl H_2_C-5′), 4.00–4.03 (m, 2H, downfield H of piperidinyl H_2_C-2″ + downfield H of piperidinyl H_2_C-6″), 4.71 (t, *J* = 9.1 Hz, 1H, pyrrolidinyl H-4′), 6.70 (d, *J* = 7.7 Hz, 1H, arom. H), 6.87 (br d, 2H, arom. H), 7.09–7.12 (m, 1H, arom. H), 7.22 (d, *J* = 6.8 Hz, 2H, arom. H), 7.28 (t, *J* = 7.3 Hz, 1H, arom. H), 7.35–7.41 (m, 5H, arom. H), 7.46–7.49 (m, 3H, 2 arom. H + olefinic CH), 10.58 (s, 1H, NH). ^13^C-NMR (DMSO-*d*_*6*_) *δ* (ppm): 7.6 (CH_3_CH_2_S), 34.3 (NCH_3_), 42.0 (CH_2_S), 46.4 (pyrrolidinyl HC-4′), 47.0 (piperidinyl H_2_C-6″), 48.8 (piperidinyl H_2_C-2″), 57.7 (pyrrolidinyl H_2_C-5′), 61.9 [spiro-C-3′ (C-3″)], 75.8 [spiro-C-3 (C-2′)], 109.7, 121.3, 125.5, 127.4, 127.7, 128.8, 129.2, 129.7, 130.0, 130.3, 130.4, 130.6, 134.4, 138.3, 139.3, 144.1 (arom. C + olefinic C), 175.7, 196.4 (C=O). Anal. Calcd. for C_31_H_31_N_3_O_4_S (541.67): C, 68.74; H, 5.77; N, 7.76. Found: C, 69.05; H, 5.84; N, 7.99.

#### (*E*)-5″-Benzylidene-5-chloro-1″-(ethylsulfonyl)-1′-methyl-4′-phenyldispiro[indoline-3,2′-pyrrolidine-3′,3″-piperidine]-2,4″-dione (**6d**)

Obtained from the reaction of **3b**, **4b** and **5**, reaction time 8 h as colorless microcrystals from n-butanol, with mp 207‒209 °C and yield 85% (1.22 g). IR: *ν*_max_/cm^−1^ 3159, 1713, 1682, 1615, 1597. ^1^H-NMR (DMSO-*d*_*6*_) *δ* (ppm): 0.98 (t, *J* = 7.4 Hz, 3H, CH_3_CH_2_S), 1.98 (s, 3H, NCH_3_), 2.39 (d, *J* = 13.0 Hz, 1H, upfield H of piperidinyl H_2_C-2″), 2.77 (sextet, *J* = 7.3, 14.2 Hz, 1H, upfield H of CH_3_CH_2_S), 2.93 (sextet, *J* = 7.4, 14.8 Hz, 1H, downfield H of CH_3_CH_2_S), 3.35 (t, *J* = 8.4 Hz, 1H, upfield H of pyrrolidinyl H_2_C-5′), 3.74 (d, *J* = 14.9 Hz, 1H, upfield H of piperidinyl H_2_C-6″), 3.82 (t, *J* = 9.4 Hz, 1H, downfield H of pyrrolidinyl H_2_C-5′), 4.03 (d, *J* = 13.0, 1H, downfield H of piperidinyl H_2_C-2″), 4.07 (dd, *J* = 2.5, 15.0 Hz, 1H, downfield H of piperidinyl H_2_C-6″), 4.69 (t, *J* = 9.1 Hz, 1H, pyrrolidinyl H-4′), 6.71 (d, *J* = 8.3 Hz, 1H, arom. H), 6.81 (d, *J* = 2.2 Hz, 1H, arom. H), 7.17 (dd, *J* = 2.2, 8.3 Hz, 1H, arom. H), 7.27–7.30 (m, 3H, arom. H), 7.35–7.52 (m, 8H, 7 arom. H + olefinic CH), 10.75 (s, 1H, NH). ^13^C-NMR (DMSO-*d*_*6*_) *δ* (ppm): 7.0 (CH_3_CH_2_S), 33.8 (NCH_3_), 41.2 (CH_2_S), 46.0 (pyrrolidinyl HC-4′), 47.0 (piperidinyl H_2_C-6″), 48.7 (piperidinyl H_2_C-2″), 57.5 (pyrrolidinyl H_2_C-5′), 61.7 [spiro-C-3′ (C-3″)], 75.3 [spiro-C-3 (C-2′)], 110.6, 124.9, 126.7, 127.15, 127.18, 128.3, 128.7, 128.9, 129.7, 129.8, 129.9, 130.1, 133.7, 137.6, 139.0, 142.5 (arom. C + olefinic C), 174.7, 195.8 (C=O). Anal. Calcd. for C_31_H_30_ClN_3_O_4_S (576.11): C, 64.63; H, 5.25; N, 7.29. Found: C, 64.49; H, 5.37; N, 7.45.

#### (*E*)-5″-(4-Fluorobenzylidene)-4′-(4-fluorophenyl)-1′-methyl-1″-(methylsulfonyl)dispiro[indoline-3,2′-pyrrolidine-3′,3″-piperidine]-2,4″-dione (**6e**)

Obtained from the reaction of **3c**, **4a** and **5**, reaction time 5 h as colorless microcrystals from n-butanol, with mp 217‒219 °C and yield 82% (1.15 g). IR: *ν*_max_/cm^−1^ 3186, 1701, 1620, 1601. ^1^H-NMR (DMSO-*d*_*6*_) *δ* (ppm): 1.95 (s, 3H, NCH_3_), 2.31 (d, *J* = 12.7 Hz, 1H, upfield H of piperidinyl H_2_C-2″), 2.69 (s, 3H, SCH_3_), 3.35 (m, 1H, upfield H of pyrrolidinyl H_2_C-5′), 3.51 (d, *J* = 15.0 Hz, 1H, upfield H of piperidinyl H_2_C-6″), 3.77 (t, *J* = 9.3 Hz, 1H, downfield H of pyrrolidinyl H_2_C-5′), 3.93 (t, *J* = 13.4 Hz, 2H, downfield H of piperidinyl H_2_C-2″ + downfield H of piperidinyl H_2_C-6″), 4.69 (t, *J* = 9.0 Hz, 1H, pyrrolidinyl H-4′), 6.70 (d, *J* = 7.7 Hz, 1H, arom. H), 6.84–6.86 (m, 2H, arom. H), 7.09 (quintet, *J* = 2.4, 8.1 Hz, 1H, arom. H), 7.19 (t, *J* = 8.6 Hz, 2H, arom. H), 7.25 (t, *J* = 8.7 Hz, 2H, arom. H), 7.32–7.34 (m, 2H, arom. H), 7.51–7.53 (m, 3H, 2 arom. H + olefinic CH), 10.61 (s, 1H, NH). ^13^C-NMR (DMSO-*d*_*6*_) *δ* (ppm): 33.6 (NCH_3_, SCH_3_), 45.0 (pyrrolidinyl HC-4′), 46.5 (piperidinyl H_2_C-6″), 47.8 (piperidinyl H_2_C-2″), 57.7 (pyrrolidinyl H_2_C-5′), 61.0 [spiro-C-3′ (C-3″)], 75.5 [spiro-C-3 (C-2′)], 109.1, 115.0, 115.7, 115.8, 120.7, 124.8, 126.8, 129.2, 129.4, 130.41, 130.43, 131.67, 131.74, 132.5, 132.6, 133.89, 133.91, 137.7, 143.6, 160.3, 161.4, 162.2, 163.4 (arom. C + olefinic C), 175.1, 195.6 (C=O). Anal. Calcd. for C_30_H_27_F_2_N_3_O_4_S (563.62): C, 63.93; H, 4.83; N, 7.46. Found: C, 64.16; H, 5.01; N, 7.66.

#### (*E*)-5-Chloro-5″-(4-fluorobenzylidene)-4′-(4-fluorophenyl)-1′-methyl-1″-(methylsulfonyl)dispiro[indoline-3,2′-pyrrolidine-3′,3″-piperidine]-2,4″-dione (**6f**)

Obtained from the reaction of **3c**, **4b** and **5**, reaction time 4 h as colorless microcrystals from n-butanol, with mp 212‒214 °C and yield 74% (1.10 g). IR: *ν*_max_/cm^−1^ 3067, 1728, 1686, 1597, 1582. ^1^H-NMR (DMSO-*d*_*6*_) *δ* (ppm): 1.97 (s, 3H, NCH_3_), 2.34 (d, *J* = 12.8 Hz, 1H, upfield H of piperidinyl H_2_C-2″), 2.71 (s, 3H, SCH_3_), 3.37 (t, *J* = 8.4 Hz, 1H, upfield H of pyrrolidinyl H_2_C-5′), 3.68 (d, *J* = 14.9 Hz, 1H, upfield H of piperidinyl H_2_C-6″), 3.74 (t, *J* = 9.4 Hz, 1H, downfield H of pyrrolidinyl H_2_C-5′), 3.92 (d, *J* = 12.7 Hz, 1H, downfield H of piperidinyl H_2_C-2″), 4.00 (d, *J* = 15.0 Hz, 1H, downfield H of piperidinyl H_2_C-6″), 4.66 (t, *J* = 9.0 Hz, 1H, pyrrolidinyl H-4′), 6.70 (d, *J* = 8.3 Hz, 1H, arom. H), 6.77 (d, *J* = 1.9 Hz, 1H, arom. H), 7.16 (dd, *J* = 2.0, 8.4 Hz, 1H, arom. H), 7.20 (t, *J* = 8.7 Hz, 2H, arom. H), 7.29 (t, *J* = 8.8 Hz, 2H, arom. H), 7.38–7.40 (m, 2H, arom. H), 7.51–7.53 (m, 3H, 2 arom. H + olefinic CH), 10.78 (s, 1H, NH). ^13^C-NMR (DMSO-*d*_*6*_) *δ* (ppm): 33.4, 33.8 (NCH_3_, SCH_3_), 45.1 (pyrrolidinyl HC-4′), 47.0 (piperidinyl H_2_C-6″), 48.1 (piperidinyl H_2_C-2″), 57.9 (pyrrolidinyl H_2_C-5′), 61.3 [spiro-C-3′ (C-3″)], 75.5 [spiro-C-3 (C-2′)], 110.6, 115.0, 115.2, 115.7, 115.9, 124.8, 126.7, 127.0, 129.0, 129.5, 130.2, 131.73, 131.79, 132.62, 132.69, 133.7, 137.8, 142.5, 160.3, 161.5, 162.2, 163.5 (arom. C + olefinic C), 174.8, 195.5 (C=O). Anal. Calcd. for C_30_H_26_ClF_2_N_3_O_4_S (598.06): C, 60.25; H, 4.38; N, 7.03. Found: C, 60.44; H, 4.54; N, 7.17.

#### (*E*)-1″-(Ethylsulfonyl)-5″-(4-fluorobenzylidene)-4′-(4-fluorophenyl)-1′-methyldispiro[indoline-3,2′-pyrrolidine-3′,3″-piperidine]-2,4″-dione (**6g**)

Obtained from the reaction of **3d**, **4a** and **5**, reaction time 4 h as yellow microcrystals from n-butanol, with mp 220‒222 °C and yield 76% (1.09 g). IR: *ν*_max_/cm^−1^ 3182, 1713, 1682, 1620, 1601. ^1^H-NMR (DMSO-*d*_*6*_) *δ* (ppm): 1.00 (t, *J* = 7.4 Hz, 3H, CH_3_CH_2_S), 1.96 (s, 3H, NCH_3_), 2.39 (d, *J* = 12.9 Hz, 1H, upfield H of piperidinyl H_2_C-2″), 2.79 (sextet, *J* = 7.3, 14.6 Hz, 1H, upfield H of CH_3_CH_2_S), 2.92 (sextet, *J* = 7.4, 14.8 Hz, 1H, downfield H of CH_3_CH_2_S), 3.35 (t, *J* = 8.4 Hz, 1H, upfield H of pyrrolidinyl H_2_C-5′), 3.52 (d, *J* = 15.0 Hz, 1H, upfield H of piperidinyl H_2_C-6″), 3.77 (t, *J* = 9.4 Hz, 1H, downfield H of pyrrolidinyl H_2_C-5′), 3.96 (d, *J* = 12.8 Hz, 1H, downfield H of piperidinyl H_2_C-2″), 4.01 (dd, *J* = 2.2, 15.0 Hz, 1H, downfield H of piperidinyl H_2_C-6″), 4.69 (t, *J* = 9.0 Hz, 1H, pyrrolidinyl H-4′), 6.71 (d, *J* = 7.7 Hz, 1H, arom. H), 6.85–6.87 (m, 2H, arom. H), 7.09–7.12 (m, 1H, arom. H), 7.19 (t, *J* = 8.8 Hz, 2H, arom. H), 7.25 (t, *J* = 8.8 Hz, 2H, arom. H), 7.30–7.33 (m, 2H, arom. H), 7.50–7.53 (m, 3H, 2 arom. H + olefinic CH), 10.61 (s, 1H, NH). ^13^C-NMR (DMSO-*d*_*6*_) *δ* (ppm): 7.0 (CH_3_CH_2_S), 33.7 (NCH_3_), 41.3 (CH_2_S), 45.1 (pyrrolidinyl HC-4′), 46.4 (piperidinyl H_2_C-6″), 48.1 (piperidinyl H_2_C-2″), 57.6 (pyrrolidinyl H_2_C-5′), 61.2 [spiro-C-3′ (C-3″)], 75.4 [spiro-C-3 (C-2′)], 109.2, 115.0, 115.1, 115.7, 115.8, 120.7, 124.8, 126.8, 129.2, 129.60, 129.62, 130.4, 131.69, 131.76, 132.5, 132.6, 133.94, 133.96, 137.6, 143.6, 160.3, 161.4, 162.2, 163.4 (arom. C + olefinic C), 175.1, 195.7 (C=O). Anal. Calcd. for C_31_H_29_F_2_N_3_O_4_S (577.65): C, 64.46; H, 5.06; N, 7.27. Found: C, 64.68; H, 4.86; N, 6.96.

#### (*E*)-5-Chloro-1″-(ethylsulfonyl)-5″-(4-fluorobenzylidene)-4′-(4-fluorophenyl)-1′-methyldispiro[indoline-3,2′-pyrrolidine-3′,3″-piperidine]-2,4″-dione (**6h**)

Obtained from the reaction of **3d**, **4b** and **5**, reaction time 6h as colorless microcrystals from ethanol, with mp 136‒138 °C and yield 78% (1.20 g). IR: *ν*_max_/cm^−1^ 3341, 1694, 1620, 1601, 1582. ^1^H-NMR (DMSO-*d*_*6*_) *δ* (ppm): 1.07 (t, *J* = 7.0 Hz, 3H, CH_3_CH_2_S), 1.98 (s, 3H, NCH_3_), 2.45 (d, *J* = 12.9 Hz, 1H, upfield H of piperidinyl H_2_C-2″), 2.82 (sextet, *J* = 7.3, 14.6 Hz, 1H, upfield H of CH_3_CH_2_S), 2.96 (sextet, *J* = 7.4, 14.8 Hz, 1H, downfield H of CH_3_CH_2_S), 3.38 (t, *J* = 8.5 Hz, 1H, upfield H of pyrrolidinyl H_2_C-5′), 3.71 (d, *J* = 15.0 Hz, 1H, upfield H of piperidinyl H_2_C-6″), 3.76 (t, *J* = 9.4 Hz, 1H, downfield H of pyrrolidinyl H_2_C-5′), 3.99 (d, *J* = 12.9 Hz, 1H, downfield H of piperidinyl H_2_C-2″), 4.08 (dd, *J* = 2.2, 14.9 Hz, 1H, downfield H of piperidinyl H_2_C-6″), 4.68 (t, *J* = 9.0 Hz, 1H, pyrrolidinyl H-4′), 6.72 (d, *J* = 8.3 Hz, 1H, arom. H), 6.80 (d, *J* = 2.1 Hz, 1H, arom. H), 7.17 (dd, *J* = 2.3, 8.2 Hz, 1H, arom. H), 7.19 (t, *J* = 8.5 Hz, 2H, arom. H). 7.29 (t, *J* = 8.8 Hz, 2H, arom. H), 7.37–7.40 (m, 2H, arom. H), 7.52–7.55 (m, 3H, arom. H + olefinic CH), 10.78 (s, 1H, NH). ^13^C-NMR (DMSO-*d*_*6*_) *δ* (ppm): 7.0 (CH_3_CH_2_S), 33.9 (NCH_3_), 41.2 (CH_2_S), 45.3 (pyrrolidinyl HC-4′), 47.0 (piperidinyl H_2_C-6″), 48.6 (piperidinyl H_2_C-2″), 57.9 (pyrrolidinyl H_2_C-5′), 61.6 [spiro-C-3′ (C-3″)], 75.5 [spiro-C-3 (C-2′)], 110.7, 115.0, 115.2, 115.8, 116.0, 125.0, 126.8, 127.1, 129.0, 129.77, 129.79, 130.28, 130.30, 131.81, 131.87, 132.60, 132.67, 133.8, 137.8, 142.6, 160.4, 161.6, 162.3, 163.6 (arom. C + olefinic C), 174.8, 195.7 (C=O). Anal. Calcd. for C_31_H_28_ClF_2_N_3_O_4_S (612.09): C, 60.83; H, 4.61; N, 6.87. Found: C, 61.01; H, 4.75; N, 6.96.

#### (*E*)-5″-(4-Chlorobenzylidene)-4′-(4-chlorophenyl)-1′-methyl-1″-(methylsulfonyl)dispiro[indoline-3,2′-pyrrolidine-3′,3″-piperidine]-2,4″-dione (**6i**)

Obtained from the reaction of **3e**, **4a** and **5**, reaction time 4 h as colorless microcrystals from ethanol, with mp 221‒223 °C and yield 87% (1.30 g). IR: *ν*_max_/cm^−1^ 3387, 1697, 1618, 1605. ^1^H-NMR (DMSO-*d*_*6*_) *δ* (ppm): 1.95 (s, 3H, NCH_3_), 2.34 (d, *J* = 12.7 Hz, 1H, upfield H of piperidinyl H_2_C-2″), 2.70 (s, 3H, SCH_3_), 3.34 (t, *J* = 8.1 Hz, 1H, upfield H of pyrrolidinyl H_2_C-5′), 3.51 (d, *J* = 15.1 Hz, 1H, upfield H of piperidinyl H_2_C-6″), 3.77 (t, *J* = 9.3 Hz, 1H, downfield H of pyrrolidinyl H_2_C-5′), 3.91–3.95 (m, 2H, downfield H of piperidinyl H_2_C-2″ + downfield H of piperidinyl H_2_C-6″), 4.68 (t, *J* = 9.0 Hz, 1H, pyrrolidinyl H-4′), 6.70 (d, *J* = 7.7 Hz, 1H, arom. H), 6.84–6.85 (m, 2H, arom. H), 7.08–7.11 (m, 1H, arom. H), 7.28 (d, *J* = 8.4 Hz, 2H, arom. H), 7.42 (d, *J* = 8.3 Hz, 2H, arom. H), 7.46–7.51 (m, 5H, 4 arom. H + olefinic CH), 10.62 (s, 1H, NH). ^13^C-NMR (DMSO-*d*_*6*_) *δ* (ppm): 33.7 (NCH_3_, SCH_3_), 45.1 (pyrrolidinyl HC-4′), 46.5 (piperidinyl H_2_C-6″), 47.7 (piperidinyl H_2_C-2″), 57.5 (pyrrolidinyl H_2_C-5′), 61.1 [spiro-C-3′ (C-3″)], 75.4 [spiro-C-3 (C-2′)], 109.2, 120.7, 124.7, 126.9, 128.3, 128.7, 129.2, 130.1, 131.7, 131.8, 132.7, 134.3, 136.8, 137.5, 143.6 (arom. C + olefinic C), 175.1, 195.5 (C=O). Anal. Calcd. for C_30_H_27_Cl_2_N_3_O_4_S (596.52): C, 60.41; H, 4.56; N, 7.04. Found: C, 60.16; H, 4.30; N, 6.90.

#### (*E*)-5-Chloro-5″-(4-chlorobenzylidene)-4′-(4-chlorophenyl)-1′-methyl-1″-(methylsulfonyl)dispiro[indoline-3,2′-pyrrolidine-3′,3″-piperidine]-2,4″-dione (**6j**)

Obtained from the reaction of **3e**, **4b** and **5**, reaction time 8 h as colorless microcrystals from n-butanol, with mp 219‒221 °C and yield 74% (1.17 g). IR: *ν*_max_/cm^−1^ 3136, 3098, 1730, 1694, 1612. ^1^H-NMR (DMSO-*d*_*6*_) *δ* (ppm): 1.97 (s, 3H, NCH_3_), 2.40 (d, *J* = 12.8 Hz, 1H, upfield H of piperidinyl H_2_C-2″), 2.73 (s, 3H, SCH_3_), 3.38 (t, *J* = 8.8 Hz, 1H, upfield H of pyrrolidinyl H_2_C-5′), 3.69 (d, *J* = 15.0 Hz, 1H, upfield H of piperidinyl H_2_C-6″), 3.75 (t, *J* = 9.4 Hz, 1H, downfield H of pyrrolidinyl H_2_C-5′), 3.94 (d, *J* = 12.7 Hz, 1H, downfield H of piperidinyl H_2_C-2″), 4.01 (d, *J* = 15.1 Hz, 1H, downfield H of piperidinyl H_2_C-6″), 4.66 (t, *J* = 9.0 Hz, 1H, pyrrolidinyl H-4′), 6.71 (d, *J* = 8.3 Hz, 1H, arom. H), 6.77 (d, *J* = 1.7 Hz, 1H, arom. H), 7.16 (dd, *J* = 2.0, 8.3 Hz, 1H, arom. H), 7.34 (d, *J* = 8.5 Hz, 2H, arom. H), 7.42 (d, *J* = 8.3 Hz, 2H, arom. H), 7.50–7.52 (m, 5H, 4 arom. H + olefinic CH), 10.79 (s, 1H, NH). ^13^C-NMR (DMSO-*d*_*6*_) *δ* (ppm): 33.6, 33.8 (NCH_3_, SCH_3_), 45.2 (pyrrolidinyl HC-4′), 47.0 (piperidinyl H_2_C-6″), 48.0 (piperidinyl H_2_C-2″), 57.8 (pyrrolidinyl H_2_C-5′), 61.4 [spiro-C-3′ (C-3″)], 75.5 [spiro-C-3 (C-2′)], 110.7, 124.9, 126.7, 127.0, 128.3, 128.8, 129.0, 130.3, 131.8, 131.9, 132.5, 134.5, 136.6, 137.6, 142.6 (arom. C + olefinic C), 174.7, 195.3 (C=O). Anal. Calcd. for C_30_H_26_Cl_3_N_3_O_4_S (630.97): C, 57.11; H, 4.15; N, 6.66. Found: C, 57.21; H, 3.96; N, 6.88.

#### (*E*)-5″-(4-Chlorobenzylidene)-4′-(4-chlorophenyl)-1″-(ethylsulfonyl)-1′-methyldispiro[indoline-3,2′-pyrrolidine-3′,3″-piperidine]-2,4″-dione (**6k**)

Obtained from the reaction of **3f**, **4a** and **5**, reaction time 5 h as colorless microcrystals from ethanol, with mp 136‒138 °C and yield 86% (1.31 g). IR: *ν*_max_/cm^−1^ 3379, 1697, 1619, 1601. ^1^H-NMR (DMSO-*d*_*6*_) *δ* (ppm): 1.01 (t, *J* = 7.4 Hz, 3H, CH_3_CH_2_S), 1.95 (s, 3H, NCH_3_), 2.42 (d, *J* = 12.9 Hz, 1H, upfield H of piperidinyl H_2_C-2″), 2.78 (sextet, *J* = 7.3, 14.5 Hz, 1H, upfield H of CH_3_CH_2_S), 2.93 (sextet, *J* = 7.4, 14.7 Hz, 1H, downfield H of CH_3_CH_2_S), 3.34 (t, *J* = 8.3 Hz, 1H, upfield H of pyrrolidinyl H_2_C-5′), 3.51 (d, *J* = 15.1 Hz, 1H, upfield H of piperidinyl H_2_C-6″), 3.76 (t, *J* = 9.3 Hz, 1H, downfield H of pyrrolidinyl H_2_C-5′), 3.96 (d, *J* = 12.8 Hz, 1H, downfield H of piperidinyl H_2_C-2″), 4.00 (dd, *J* = 1.7, 15.1 Hz, 1H, downfield H of piperidinyl H_2_C-6″), 4.68 (t, *J* = 9.0 Hz, 1H, pyrrolidinyl H-4′), 6.70 (d, *J* = 7.7 Hz, 1H, arom. H), 6.85 (s, 1H, arom. H), 6.86 (s, 1H, arom. H), 7.09–7.12 (m, 1H, arom. H), 7.25 (d, *J* = 8.5 Hz, 2H, arom. H), 7.42 (d, *J* = 8.4 Hz, 2H, arom. H), 7.46–7.51 (m, 5H, 4 arom. H + olefinic CH), 10.61 (s, 1H, NH). ^13^C-NMR (DMSO-*d*_*6*_) *δ* (ppm): 7.0 (CH_3_CH_2_S), 33.7 (NCH_3_), 41.4 (CH_2_S), 45.2 (pyrrolidinyl HC-4′), 46.4 (piperidinyl H_2_C-6″), 48.0 (piperidinyl H_2_C-2″), 57.4 (pyrrolidinyl H_2_C-5′), 61.3 [spiro-C-3′ (C-3″)], 75.3 [spiro-C-3 (C-2′)], 109.3, 120.7, 124.7, 126.9, 128.4, 128.7, 129.2, 130.4, 131.7, 132.7, 134.2, 136.8, 137.4, 143.6 (arom. C + olefinic C), 175.1, 195.6 (C=O). Anal. Calcd. for C_31_H_29_Cl_2_N_3_O_4_S (610.55): C, 60.98; H, 4.79; N, 6.88. Found: C, 60.81; H, 4.63; N, 6.75.

#### (*E*)-5-Chloro-5″-(4-chlorobenzylidene)-4′-(4-chlorophenyl)-1″-(ethylsulfonyl)-1′-methyldispiro[indoline-3,2′-pyrrolidine-3′,3″-piperidine]-2,4″-dione (**6l**)

Obtained from the reaction of **3f**, **4b** and **5**, reaction time 6h as colorless microcrystals from ethanol, with mp 133‒136 °C and yield 75% (1.20 g). IR: *ν*_max_/cm^−1^ 3356, 1694, 1620, 1597. ^1^H-NMR (DMSO-*d*_*6*_) *δ* (ppm): 1.01 (t, *J* = 7.4 Hz, 3H, CH_3_CH_2_S), 1.95 (s, 3H, NCH_3_), 2.42 (d, *J* = 12.9 Hz, 1H, upfield H of piperidinyl H_2_C-2″), 2.79 (sextet, *J* = 7.2, 14.2 Hz, 1H, upfield H of CH_3_CH_2_S), 2.93 (sextet, *J* = 7.4, 14.7 Hz, 1H, downfield H of CH_3_CH_2_S), 3.34 (t, *J* = 8.3 Hz, 1H, upfield H of pyrrolidinyl H_2_C-5′), 3.51 (d, *J* = 15.1 Hz, 1H, upfield H of piperidinyl H_2_C-6″), 3.76 (t, *J* = 9.3 Hz, 1H, downfield H of pyrrolidinyl H_2_C-5′), 3.96 (d, *J* = 12.8 Hz, 1H, downfield H of piperidinyl H_2_C-2″), 4.00 (dd, *J* = 1.7, 15.1 Hz, 1H, downfield H of piperidinyl H_2_C-6″), 4.68 (t, *J* = 9.0 Hz, 1H, pyrrolidinyl H-4′), 6.70 (d, *J* = 7.7 Hz, 1H, arom. H), 6.85 (s, 1H, arom. H), 6.86 (s, 1H, arom. H), 7.09–7.12 (m, 1H, arom. H), 7.25 (d, *J* = 8.5 Hz, 2H, arom. H), 7.42 (d, *J* = 8.4 Hz, 2H, arom. H), 7.46–7.51 (m, 4H, 3 arom. H + olefinic CH), 10.61 (s, 1H, NH). ^13^C-NMR (DMSO-*d*_*6*_) *δ* (ppm): 6.9 (CH_3_CH_2_S), 33.8 (NCH_3_), 41.2 (CH_2_S), 45.4 (pyrrolidinyl HC-4′), 46.9 (piperidinyl H_2_C-6″), 48.4 (piperidinyl H_2_C-2″), 57.7 (pyrrolidinyl H_2_C-5′), 61.6 [spiro-C-3′ (C-3″)], 75.5 [spiro-C-3 (C-2′)], 110.8, 124.9, 126.7, 127.0, 128.3, 128.8, 129.1, 130.5, 131.81, 131.84, 131.86, 132.5, 134.5, 136.6, 137.5, 142.6 (arom. C + olefinic C), 174.8, 195.5 (C=O). Anal. Calcd. for C_31_H_28_Cl_3_N_3_O_4_S (644.99): C, 57.73; H, 4.38; N, 6.51. Found: C, 57.52; H, 4.28; N, 6.57.

#### (*E*)-5″-(4-Bromobenzylidene)-4′-(4-bromophenyl)-1′-methyl-1″-(methylsulfonyl)dispiro[indoline-3,2′-pyrrolidine-3′,3″-piperidine]-2,4″-dione (**6m**)

Obtained from the reaction of **3g**, **4a** and **5**, reaction time 12 h as pale yellow microcrystals from n-butanol, with mp 224‒226 °C and yield 70% (1.20 g). IR: *ν*_max_/cm^−1^ 3306, 1717, 1674, 1610. ^1^H-NMR (DMSO-*d*_*6*_) *δ* (ppm): 1.95 (s, 3H, NCH_3_), 2.35 (d, *J* = 12.7 Hz, 1H, upfield H of piperidinyl H_2_C-2″), 2.70 (s, 3H, SCH_3_), 3.35 (t, *J* = 6.5 Hz, 1H, upfield H of pyrrolidinyl H_2_C-5′), 3.50 (d, *J* = 15.1 Hz, 1H, upfield H of piperidinyl H_2_C-6″), 3.76 (t, *J* = 9.3 Hz, 1H, downfield H of pyrrolidinyl H_2_C-5′), 3.91–3.94 (m, 2H, downfield H of piperidinyl H_2_C-2″ + downfield H of piperidinyl H_2_C-6″), 4.67 (t, *J* = 8.9 Hz, 1H, pyrrolidinyl H-4′), 6.70 (d, *J* = 7.7 Hz, 1H, arom. H), 6.84–6.85 (br d, 2H, arom. H), 7.08–7.11 (m, 1H, arom. H), 7.20 (d, *J* = 8.3 Hz, 2H, arom. H), 7.44–7.73 (m, 7H, 6 arom. H + olefinic CH), 10.62 (s, 1H, NH). ^13^C-NMR (DMSO-*d*_*6*_) *δ* (ppm): 33.7, 36.0 (NCH_3_, SCH_3_), 45.1 (pyrrolidinyl HC-4′), 46.5 (piperidinyl H_2_C-6″), 47.7 (piperidinyl H_2_C-2″), 57.4 (pyrrolidinyl H_2_C-5′), 61.1 [spiro-C-3′ (C-3″)], 75.4 [spiro-C-3 (C-2′)], 109.2, 120.3, 120.6, 123.1, 123.3, 124.7, 126.8, 129.2, 130.2, 131.2, 131.6, 131.8, 132.0, 132.1, 132.4, 133.0, 133.1, 135.6, 137.2, 137.5, 143.6 (arom. C + olefinic C), 175.1, 195.5 (C=O). Anal. Calcd. for C_30_H_27_Br_2_N_3_O_4_S (685.43): C, 52.57; H, 3.97; N, 6.13. Found: C, 52.75; H, 4.11; N, 6.30.

#### (*E*)-5″-(3,4-Dimethoxybenzylidene)-4′-(3,4-dimethoxyphenyl)-1′-methyl-1″-(methylsulfonyl)dispiro[indoline-3,2′-pyrrolidine-3′,3″-piperidine]-2,4″-dione (**6n**)

Obtained from the reaction of **3h**, **4a** and **5**, reaction time 10 h as yellow microcrystals from n-butanol, with mp 204‒206 °C and yield 93% (1.50 g). IR: *ν*_max_/cm^−1^ 3345, 1717, 1663, 1582. ^1^H-NMR (DMSO-*d*_*6*_) *δ* (ppm): 1.95 (s, 3H, NCH_3_), 2.27 (d, *J* = 12.8 Hz, 1H, upfield H of piperidinyl H_2_C-2″), 2.69 (s, 3H, SCH_3_), 3.33 (t, *J* = 8.4 Hz, 1H, upfield H of pyrrolidinyl H_2_C-5′), 3.50 (d, *J* = 14.9 Hz, 1H, upfield H of piperidinyl H_2_C-6″), 3.75–3.78 (m, 13H, 4 OCH_3_ + downfield H of pyrrolidinyl H_2_C-5′), 3.97 (dd, *J* = 1.8, 15.0 Hz, 1H, downfield H of piperidinyl H_2_C-6″), 4.01 (d, *J* = 12.8 Hz, 1H, downfield H of piperidinyl H_2_C-2″), 4.60 (t, *J* = 9.0 Hz, 1H, pyrrolidinyl H-4′), 6.70 (d, *J* = 7.7 Hz, 1H, arom. H), 6.77 (d, *J* = 7.5 Hz, 1H, arom. H), 6.82–6.86 (m, 2H, arom. H), 6.90–7.00 (m, 4H, arom. H), 7.09–7.51 (m, 3H, 2 arom. H + olefinic CH), 10.57 (s, 1H, NH). ^13^C-NMR (DMSO-*d*_*6*_) *δ* (ppm): 33.8 (NCH_3_, SCH_3_), 45.5 (pyrrolidinyl HC-4′), 46.5 (piperidinyl H_2_C-6″), 47.6 (piperidinyl H_2_C-2″), 55.3, 55.4 (OCH_3_), 57.9 (pyrrolidinyl H_2_C-5′), 61.0 [spiro-C-3′ (C-3″)], 75.5 [spiro-C-3 (C-2′)], 109.0, 111.4, 111.5, 113.6, 114.3, 120.6, 122.0, 123.4, 125.1, 126.6, 126.7, 127.6, 129.0, 130.1, 139.1, 143.5, 147.8, 148.4, 150.1 (arom. C + olefinic C), 175.3, 195.6 (C=O). Anal. Calcd. for C_34_H_37_N_3_O_8_S (647.74): C, 63.05; H, 5.76; N, 6.49. Found: C, 62.74; H, 5.94; N, 6.22.

#### (*E*)-5-Chloro-5″-(3,4-dimethoxybenzylidene)-4′-(3,4-dimethoxyphenyl)-1′-methyl-1″-(methylsulfonyl)dispiro[indoline-3,2′-pyrrolidine-3′,3″-piperidine]-2,4″-dione (**6o**)

Obtained from the reaction of **3h**, **4b** and **5**, reaction time 12 h as yellow microcrystals from n-butanol, with mp 201‒203 °C and yield 74% (1.27 g). IR: *ν*_max_/cm^−1^ 3345, 1717, 1663, 1582. ^1^H-NMR (DMSO-*d*_*6*_) *δ* (ppm): 1.96 (s, 3H, NCH_3_), 2.27 (d, *J* = 12.9 Hz, 1H, upfield H of piperidinyl H_2_C-2″), 2.69 (s, 3H, SCH_3_), 3.33 (t, *J* = 8.4 Hz, 1H, upfield H of pyrrolidinyl H_2_C-5′), 3.50 (d, *J* = 14.9 Hz, 1H, upfield H of piperidinyl H_2_C-6″), 3.75–3.78 (m, 13H, 4 OCH_3_ + downfield H of pyrrolidinyl H_2_C-5′), 3.96–4.03 (m, 2H, downfield H of piperidinyl H_2_C-2″ + downfield H of piperidinyl H_2_C-6″), 4.60 (t, *J* = 9.0 Hz, 1H, pyrrolidinyl H-4′), 6.71 (d, *J* = 7.7 Hz, 1H, arom. H), 6.77 (d, *J* = 8.4 Hz, 1H, arom. H), 6.82–6.87 (m, 2H, arom. H), 6.90–7.00 (m, 3H, arom. H), 7.09–7.51 (m, 3H, 2 arom. H + olefinic CH), 10.58 (s, 1H, NH). ^13^C-NMR (DMSO-*d*_*6*_) *δ* (ppm): 33.8 (NCH_3_, SCH_3_), 45.5 (pyrrolidinyl HC-4′), 46.5 (piperidinyl H_2_C-6″), 47.6 (piperidinyl H_2_C-2″), 55.3 (OCH_3_), 58.0 (pyrrolidinyl H_2_C-5′), 61.0 [spiro-C-3′ (C-3″)], 75.5 [spiro-C-3 (C-2′)], 109.0, 111.4, 111.5, 113.6, 114.3, 120.6, 122.0, 123.4, 125.1, 126.6, 126.7, 127.6, 129.0, 130.2, 139.1, 143.5, 147.8, 148.4, 150.1 (arom. C + olefinic C), 175.3, 195.6 (C=O). Anal. Calcd. for C_34_H_36_ClN_3_O_8_S (682.19): C, 59.86; H, 5.32; N, 6.16. Found: C, 59.97; H, 5.41; N, 6.06.

## Supplementary Information

Supplementary Information.

## Data Availability

All data generated or analyzed during this study are included in this published article and its Supplementary Information Files. The X-ray data have been deposited in the CSD with reference numbers CCDC 2087291, 2087292, 2087297, 2087299 and the Check-CIF files are also attached as supplementary files to this article.
