# Supplementary material for: Development of spiro-3-indolin-2-one containing compounds of antiproliferative and anti-SARS-CoV-2 properties
Source: Sci Rep. 2022 Aug 16;12:13880. doi: 10.1038/s41598-022-17883-9 (PMC9380671; doi:10.1038/s41598-022-17883-9)
Supplement: Supplementary file 1 — Supplementary Information. [file 41598_2022_17883_MOESM1_ESM.docx]

**Development of spiro-3-indolin-2-ones containing compounds of antiproliferative and anti-SARS-CoV-2 properties**

Nehmedo G. Fawazy^1^, Siva S. Panda^2^, Ahmed Mostafa^3^, Benson M. Kariuki^4^, Mohamed S. Bekheit^1^, Yassmin Moatasim^3^, Omnia Kutkat^3^, Walid Fayad^5^, May A. El-Manawaty^5^, Ahmed A. F. Soliman^5^, Riham A. El-Shiekh^6^, Aladdin M. Srour^7^, Reham F. Barghash^1^, Adel S. Girgis^1,^*

^1^Department of Pesticide Chemistry, National Research Centre, Dokki, Giza, 12622, Egypt

^2^Department of Chemistry and Physics, Augusta University, Augusta, GA,30912, USA

^3^Center of Scientiﬁc Excellence for Inﬂuenza Viruses, National Research Centre, Giza 12622, Egypt

^4^School of Chemistry, Cardiff University, Main Building, Park Place, Cardiff, CF10 3AT, UK

^5^Drug Bioassay-Cell Culture Laboratory, Pharmacognosy Department, National Research Centre, Dokki, Giza, 12622. Egypt

^6^Department of Pharmacognosy, Faculty of Pharmacy, Cairo University, Cairo 11562, Egypt

^7^Department of Therapeutic Chemistry, National Research Centre, Dokki, Giza 12622, Egypt

*Corresponding author, E-mail: [girgisas10@yahoo.com](mailto:girgisas10@yahoo.com)

**Supplementary materials**

**Table titles**

**Table S1.** Crystal and refinement data of compounds **6b**, **6c**, **6d** and **6h**.

**Table S2**. Torsion angles in the pyrrolidine-piperidine system. Structures **6b** and **9h** contain two independent molecules (Fig. shows the atom numbering used of the pyrrolidine and piperidine rings).

**Table S3.** Descriptors of the BMLR-QSAR model for the synthesized agents against MCF7 (breast) cancer cell line.

**Table S4.** Observed and estimated antiproliferation properties for the synthesized agents against MCF7 (breast) cancer cell line according to the BMLR-QSAR model.

**Table S5.** Molecular descriptor values of the BMLR-QSAR model for the synthesized agents against MCF7 (breast) cancer cell line.

**Table S6.** Descriptors of the BMLR-QSAR model for the synthesized agents against HCT116 (colon) cancer cell line.

**Table S7.** Observed and estimated antiproliferation properties for the synthesized agents against HCT116 (colon) cancer cell line according to the BMLR-QSAR model.

**Table S8.** Molecular descriptor values of the BMLR-QSAR model for the synthesized agents against HCT116 (colon) cancer cell line.

**Table S9.** Descriptors of the BMLR-QSAR model for the synthesized agents against A431 (skin squamous) cancer cell line.

**Table S10.** Observed and estimated antiproliferation properties for the synthesized agents against A431 (skin squamous) cancer cell line according to the BMLR-QSAR model.

**Table S11.** Molecular descriptor values of the BMLR-QSAR model for the synthesized agents against A431 (skin squamous) cancer cell line.

**Table S12.** Descriptors of the BMLR-QSAR model for the synthesized agents against PaCa2 (pancreatic) cancer cell line.

**Table S13.** Observed and estimated antiproliferation properties for the synthesized agents against PaCa2 (pancreatic) cancer cell line according to the BMLR-QSAR model.

**Table S14.** Molecular descriptor values of the BMLR-QSAR model for the synthesized agents against PaCa2 (pancreatic) cancer cell line.

**Table S15.** Descriptors of the BMLR-QSAR model for the synthesized agents against SARS-CoV-2.

**Table S16.** Observed and estimated properties for the synthesized agents against SARS-CoV-2 according to the BMLR-QSAR model.

**Table S17.** Molecular descriptor values of the BMLR-QSAR model for the synthesized agents against SARS-CoV-2.

**Figure legends**

**Fig. S1.** IR spectrum of compound **6a** (KBr pellet).

**Fig. S2.** ^1^H-NMR spectrum of compound **6a** in DMSO-*d_6_*.

**Fig. S3.** ^13^C-NMR spectrum of compound **6a** in DMSO-*d_6_*.

**Fig. S4.** IR spectrum of compound **6b** (KBr pellet).

**Fig. S5.** ^1^H-NMR spectrum of compound **6b** in DMSO-*d_6_*.

**Fig. S6.** ^13^C-NMR spectrum of compound **6b** in DMSO-*d_6_*.

**Fig. S7.** IR spectrum of compound **6c** (KBr pellet).

**Fig. S8.** ^1^H-NMR spectrum of compound **6c** in DMSO-*d_6_*.

**Fig. S9.** ^13^C-NMR spectrum of compound **6c** in DMSO-*d_6_*.

**Fig. S10.** IR spectrum of compound **6d** (KBr pellet).

**Fig. S11.** ^1^H-NMR spectrum of compound **6d** in DMSO-*d_6_*.

**Fig. S12.** ^13^C-NMR spectrum of compound **6d** in DMSO-*d_6_*.

**Fig. S13.** IR spectrum of compound **6e** (KBr pellet).

**Fig. S14.** ^1^H-NMR spectrum of compound **6e** in DMSO-*d_6_*.

**Fig. S15.** ^13^C-NMR spectrum of compound **6e** in DMSO-*d_6_*.

**Fig. S16.** IR spectrum of compound **6f** (KBr pellet).

**Fig. S17.** ^1^H-NMR spectrum of compound **6f** in DMSO-*d_6_*.

**Fig. S18.** ^13^C-NMR spectrum of compound **6f** in DMSO-*d_6_*.

**Fig. S19A.** ^1^H, ^1^H- COSY spectrum of compound **6f** in DMSO-*d_6_*.

**Fig. S19B.** ^1^H, ^1^H- COSY spectrum of compound **6f** in DMSO-*d_6_* (expansion of δ = 1.4-5.6).

**Fig. S20A.** HSQC spectrum of compound **6f** in DMSO-*d_6_*.

**Fig. S20B.** HSQC spectrum of compound **6f** in DMSO-*d_6_*.(expansion of δ_H_ = 0-6.0; δ_C_ = 5-75).

**Fig. S21.** IR spectrum of compound **6g** (KBr pellet).

**Fig. S22.** ^1^H-NMR spectrum of compound **6g** in DMSO-*d_6_*.

**Fig. S23.** ^13^C-NMR spectrum of compound **6g** in DMSO-*d_6_*.

**Fig. S24.** IR spectrum of compound **6h** (KBr pellet).

**Fig. S25.** ^1^H-NMR spectrum of compound **6h** in DMSO-*d_6_* (the signals at δ = 1.02 and 3.46, 4.37 are due to the solvent of crystallization, ethanol, Org. Process Res. Dev. 2016, 20, 661−667).

**Fig. S26.** ^13^C-NMR spectrum of compound **6h** in DMSO-*d_6_*.

**Fig. S27.** IR spectrum of compound **6i** (KBr pellet).

**Fig. S28.** ^1^H-NMR spectrum of compound **6i** in DMSO-*d_6_* (the signals at δ = 1.06 and 3.45, 4.35 are due to the solvent of crystallization, ethanol, Org. Process Res. Dev. 2016, 20, 661−667).

**Fig. S29.** ^13^C-NMR spectrum of compound **6i** in DMSO-*d_6_*.

**Fig. S30.** IR spectrum of compound **6j** (KBr pellet).

**Fig. S31.** ^1^H-NMR spectrum of compound **6j** in DMSO-*d_6_*.

**Fig. S32.** ^13^C-NMR spectrum of compound **6j** in DMSO-*d_6_*.

**Fig. S33.** IR spectrum of compound **6k** (KBr pellet).

**Fig. S34.** ^1^H-NMR spectrum of compound **6k** in DMSO-*d_6_* (the signals at δ = 1.06 and 3.45, 4.35 are due to the solvent of crystallization, ethanol, Org. Process Res. Dev. 2016, 20, 661−667).

**Fig. S35.** ^13^C-NMR spectrum of compound **6k** in DMSO-*d_6_*.

**Fig. S36.** IR spectrum of compound **6l** (KBr pellet).

**Fig. S37.** ^1^H-NMR spectrum of compound **6l** in DMSO-*d_6_* (the signals at δ = 1.06 and 3.45, 4.35 are due to the solvent of crystallization, ethanol, Org. Process Res. Dev. 2016, 20, 661−667).

**Fig. S38.** ^13^C-NMR spectrum of compound **6l** in DMSO-*d_6_*.

**Fig. S39.** IR spectrum of compound **6m** (KBr pellet).

**Fig. S40.** ^1^H-NMR spectrum of compound **6m** in DMSO-*d_6_*.

**Fig. S41.** ^13^C-NMR spectrum of compound **6m** in DMSO-*d_6_*.

**Fig. S42.** IR spectrum of compound **6n** (KBr pellet).

**Fig. S43.** ^1^H-NMR spectrum of compound **6n** in DMSO-*d_6_*.

**Fig. S44.** ^13^C-NMR spectrum of compound **6n** in DMSO-*d_6_*.

**Fig. S45.** IR spectrum of compound **6o** (KBr pellet).

**Fig. S46.** ^1^H-NMR spectrum of compound **6o** in DMSO-*d_6_*.

**Fig. S47.** ^13^C-NMR spectrum of compound **6o** in DMSO-*d_6_*.

**Fig. S48.** Dose-response curve for the tested compounds against MCF7 (breast cancer) cell line.

**Fig. S49.** Dose-response curve for the tested compounds against HCT116 (colon cancer) cell line.

**Fig. S50.** Dose-response curve for the tested compounds against A431 (skin squamous) cancer cell line.

**Fig. S51.** Dose-response curve for the tested compounds against PaCa (pancreatic cancer) cell line.

**Fig. S52.** Dose-response curve for the tested compounds against RPE1 (retinal pigment epithelium) cell line.

**Fig. S53.** Western blot of the tested compounds against EGFR and VEGFR-2 utilizing MTT-IC_50_ values against MCF7 (breast) cancer cell line.

**Fig. S54.** QSAR plot representing the observed versus predicted IC_50_ (*μ*M) for the synthesized agents against MCF7 (breast) cancer cell line.

**Fig. S55.** QSAR plot representing the observed versus predicted IC_50_ (*μ*M) for the synthesized agents against HCT116 (colon) cancer cell line.

**Fig. S56.** QSAR plot representing the observed versus predicted log(IC_50_, *μ*M) for the synthesized agents against A431 (skin squamous) cancer cell line.

**Fig. S57.** QSAR plot representing the observed versus predicted IC_50_ (*μ*M) for the synthesized agents against PaCa2 (pancreatic) cancer cell line.

**Fig. S58.** QSAR plot representing the observed versus predicted log(IC_50_, *μ*M) for the synthesized agents against SARS-CoV-2.

**S.1. Crystal Structure Determination**

Single-crystal XRD data were collected at room temperature on an Agilent SuperNova Dual Atlas diffractometer with a mirror monochromator using Cu radiation. The crystal structures were solved by SHELXS^1^ and refined using SHELXL^2^ Non-hydrogen atoms were refined with anisotropic displacement parameters. Hydrogen atoms were inserted in idealized positions, and a riding model was used with *Uiso* set at 1.2 or 1.5 times the value of *Ueq* for the atom to which they are bonded. For structure **6b**, one of the two independent molecules has a disordered methane-sulfonyl group with two components (40/60%). In structure **6h**, the ethane-sulfonyl and fluoro-phenyl groups of both independent molecules are disordered with occupancies for the major components ranging from 0.54 to 0.64. The structures have been deposited in the CSD with reference numbers CCDC 2087291, 2087292, 2087297, 2087299.

**S.2. Biological studies**

All the biological procedures utilized obey the standards and approved by the Research Ethics Committee, National Research Centre, Egypt (associated with project ID: 12060101).

**S.2.1. Antiproliferation properties**

The synthesized compounds were screened for their antiproliferation properties against MCF7 (breast), HCT116 (colon), A431 (skin squamous) and PaCa2 (pancreatic) cancer cell lines by the standard mitochondrial dependent reduction of yellow MTT [3-(4,5-dimethylthiazol-2-yl)-2,5-diphenyl-tetrazolium bromide] to purple formazan technique.^3^ 5-Fluorouracil (clinically accessible drug for colon, breast and skin cancers)^4,5^ and sunitinib (usable drug for gastrointestinal, renal and pancreatic cancers)^6,7^ were considered as standard references. Cells were suspended in DMEM medium for MCF7, A431, PaCa-2 and McCoy’s 5A for HCT116 in addition to 1% antibiotic–antimycotic mixture (10000 *μ*g ml^-1^ potassium penicillin, 10000 *μ*g ml^-1^ streptomycin sulfate and 25 *μ*g ml^-1^ amphotericin B), 10% fetal bovine serum and 1% L-glutamine at 37 °C, under 5% CO_2_ and 95% humidity. Cells were seeded at concentration of 30000 cells per well in fresh complete growth medium in 96-well tissue culture microtiter plates for 24 h. Media was aspirated, fresh complete medium was added and cells were incubated with different concentrations of the tested compound to give a final concentration of [50, 25, 12.5 and 6.25 *μ*M “in addition to 3.125, 1.56 and 0.78 *μ*M in case of high potent analogues”). 0.5% DMSO was used as negative control. Triplicate wells were prepared for each individual dose. After 72 h of incubation, medium was aspirated, 40 *μ*l MTT salt (2.5 mg ml^-1^) was added to each well and incubated for further 4 h at 37 °C. To stop the reaction and dissolve the formed crystals, 150 *μ*l of 10% sodium dodecyl sulfate (SDS) in deionized water was added to each well and incubated overnight at 37 °C. The absorbance was then measured at 570 nm and a reference wavelength of 595 nm.

Data were collected as mean values for experiments performed in triplicates for each individual dose which had been measured by MTT assay. Control experiments did not exhibit significant change compared to the DMSO vehicle. The cell surviving fraction was calculated according to the following equation.

*Surviving fraction* = $\frac{Opticaldensity\left( O.D. \right)oftreatedcells}{O.D.ofcontrolcells}$

The agents synthesized were also tested against RPE1 (normal human immortalized retinal pigment epithelial cell line) cell (in DMEM-F12 medium) to determine the toxicity/selectivity towards normal cells relative to the cancer cell lines utilized.

The IC_50_ (concentration required to produce 50% inhibition of cell growth compared to the control experiment) was determined using Graph-Pad PRISM version-5 software. Statistical calculations for determination of the mean and standard error mean values were determined by SPSS 16 software. The observed anti-proliferative properties are presented in Table 1 (Supplementary Figs. S48‒S52).

**S.2.2. PI-flow cytometry cell cycle studies**

Cell cycle studies for the most promising agents synthesized (**6l** and **6m**) against MCF7 (breast) cancer call were conducted by the standard technique. The Cells were suspended in DMEM medium in addition to 1% antibiotic–antimycotic mixture (10000 *μ*g ml^-1^ potassium penicillin, 10000 *μ*g ml^-1^ streptomycin sulfate and 25 *μ*g ml^-1^ amphotericin B), 10% fetal bovine serum and 1% L-glutamine at 37 °C, under 5% CO_2_ and 95% humidity. Cells were seeded at concentration of 30000 cells per well in fresh complete growth medium in 96-well tissue culture microtiter plates for 24 h. Media was aspirated, fresh complete medium was added and cells were incubated with the IC_50_ concentration of the tested compounds which determined previously in the MTT antiproliferation testing assay. The ab139418-Propidium Iodide Flow Cytometry kit for cycle analysis (reader: BD FACS Calibur)^8^ was used for cell cycle analysis. The Annexin V-FITC Apoptosis Detection Kit (Catalog#K101-25) was utilized for apoptosis studies.^9^ All the studies were conducted according to the manufacturer’s instruction.

**S.2.3. EGFR/VEGFR-2 inhibitory properties**

Western blot technique was considered for EGFR and VEGFR-2 properties determination utilizing the IC_50_ observed of each respective agent synthesized during MTT assay against MCF7 (breast) cancer cell line. All the studies were conducted according to the manufacturer’s instruction.^10,11^

**S.2.4. Anti-SARS-CoV-2 properties**

S.2.4.1. MTT cytotoxicity assay

To assess the half maximal cytotoxic concentration (CC_50_), stock solutions of the synthesized agents and standard references (favipiravir, hydroxychloroquine, chloroquine) were prepared in 10% DMSO in ddH_2_O and diluted further to the working solutions with DMEM. The cytotoxic activity of the compounds was tested in VERO-E6 cells by using the 3-(4,5-dimethylthiazol-2-yl)-2,5-diphenyltetrazolium bromide (MTT) method with minor modifications. Briefly, the cells were seeded in 96 well-plates 9100 *µ*l/well at a density of 3 x 10^5^ cell/ml and incubated for 24 h at 37 °C in 5% CO_2_. After 24 h, cells were treated with various concentrated of the tested compounds in triplicates. 24 h later, the supernatant was discarded and cell monolayers were washed with sterile 1x phosphate buffer saline (PBS) 3 times and MTT solution (20 *µ*l of 5 mg/ml) was added to each well and incubated at 37 °C for 4 h followed by medium aspiration. In each well, the formed formazan crystals were dissolved with 200 *µ*l of acidified isopropanol (0.04 M HCl in absolute isopropanol = 0.073 ml HCl in 50 ml isopropanol). Eventually, the absorbance was measured at λ_max_ 570 nm using the Anthos Zenyth 200rt plate reader (Anthos Labtec Instruments, Heerhugowaard, Netherlands). The cytotoxicity of various concentrations compared to the untreated cells was determined using nonlinear regression analysis by plotting log inhibitor versus normalized response.^12-14^

S.2.4.2. IC_50_ determination

In 96-well tissue culture plates, 2.4 x 10^4^ VERO-E6 cells were distributed in each well and incubated overnight at a humidified 37 °C incubator under 5% CO_2_ condition. The cell mononlayers were then washed with 1x PBS and subjected to virus absorption (hCoV-19/Egypt/NRC-03/2020, Accession Number on GSAID: EPI_ISL_430820) for 1 h at room temperature. The cell monolayers were further overlaid with 50 *µ*l of DMEM containing varying concentrations of the tested compounds, following incubation at 37 °C in 5% CO_2_ incubator for 72 h. The cells were fixed with 100 *µ*l of 4% paraformaldehyde for 20 min. and stained with 0.1% crystal violet in distilled water for 15 min. at room temperature. The crystal violet dye was then dissolved using 100 *µ*l absolute methanol per well and the optical density of the color is measured at 570 nm using Anthos Zenyth 200 rt plate reader (Anthos Labtec Instruments, Heerhugowaard, Netherlands). The IC_50_ of the compound is that required to reduce the virus-induced cytopathic effect (CPE) by 50 %, relative to the virus control.^12-14^

**S.2.5. Chloinesterase inhibitory properties**

The assays were undertaken by the standard technique.^15^ Briefly, 170 *μ*L of Tris-HCl buffer (200 mM, pH 7.5) was added followed by 20 *μ*L at different concentrations of tested compounds (125‒0.977 *µ*g/mL) and then 20 *μ*L of the enzyme solution (0.1 U/mL). After incubation period of 10 min at 25 °C, 40 *μ*L of DTNB (dithio-bis-(2-nitrobenzoic acid)) and then 20 *μ*L of the substrate (1.11 mM) were added. Butyrylthiocholine iodide and acetylthiocholine were utilized as substrates in BChE and AChE assays, respectively, where DTNB was served as indicator. All compounds were dissolved in MeOH. The intensity of the developed color was measured at 405 nm using a microplate reader (reading A) and control without the inhibitor were measured (reading B). Blank assays were performed by replacing the enzyme (20 *μ*L) with buffer and their absorbances were recorded for correction of the spontaneous lysis of the indicator or inherent color of the inhibitor. Linear regression was performed for calculation of the IC_50_ (50% inhibitory concentration). Microsoft EXCEL 2010 program and graph pad instate 5.0 software were used for the data analysis.

% Inhibition = [1−(corrected A/corrected B)] * 100

Selectivity index (SI) for acetylcholinesterases was calculated as follow:

SI**_(_**_AChE/BChE)_ **=** IC_50_ (AChE)/IC_50_ (BChE)

**S.3. Molecular modeling studies**

The geometry of the synthesized agents were optimized by the molecular mechanics force field (MM^+^), followed by semi-empirical AM1 method implemented in the HyperChem 8.0 package (<http://www.hyper.com>). The structures were fully optimized without constraining any parameters, thus bringing all geometric variables to their equilibrium values. The energy minimization protocol employed the Polak–Ribiere conjugated gradient algorithm.^15^ Convergence to a local minimum was achieved when the energy gradient was ≤0.01 kcal mol^-1^. The RHF (Restricted Hartree–Fock) method was used in the spin pairing for the semi-empirical tool.

2D-QSAR studies were undertaken to utilize the comprehensive descriptors for structural and statistical analysis (CODESSA-Pro) software.^15^ The optimized structures of the bio-active compounds were uploaded to CODESSA-Pro that includes MOPAC capability for the final geometry optimization. CODESSA-Pro calculated 811 (for the bio-properties against MCF7, HCT116 and A431) and 849 (for the bio-properties against PaCa2 and SARS-CoV-2) molecular descriptors (constitutional, topological, geometrical, charge-related, semi-empirical, molecular-type, atomic-type and bond-type descriptors in addition to thermodynamical in case bio-properties against PaCa2 and SARS-CoV-2) for the exported bio-active agents. Different mathematical transformations [including property, 1/property, log(property) and 1/log(property)] of the experimentally observed activity of the synthesized compounds were utilized searching for the best QSAR model. The best multi-linear regression (BMLR) technique was utilized which is a stepwise search for the best *n* parameter regression equations (where *n* stands for the number of descriptors used), based on the highest *R^2^* (squared correlation coefficient), *R^2^*cvOO (squared cross-validation “leave-one-out, LOO” coefficient), *R^2^*cvMO (squared cross-validation “leave-many-out, LMO” coefficient), *F* (Fisher statistical significance criteria) values, and *s* (standard deviation). The QSAR models were generated (obeying the thumb rule, which determines a reasonable ratio between the data points and the number of QSAR descriptor) (Tables S1‒S15, Figs. S54–S58).

**S.4. References**

| [1] | Sheldrick, G. M. A short history of SHELX. *Acta Crystallogr. A* **2008**, *64*, 112–122. https://doi:10.1107/S0108767307043930. |
| --- | --- |
| [2] | Sheldrick, G. M. Crystal structure refinement with SHELXL. *Acta Crystallogr. C* **2015**, *71*, 3–8. https://doi:10.1107/S2053229614024218. |
| [3] | Fawzy, N. G.; Panda, S. S.; Fayad, W.; Shalaby, E. M.; Srour, A. M.; Girgis, A. S. Synthesis, human topoisomerase IIα inhibitory properties and molecular modeling studies of anti-proliferative curcumin mimics. *RSC Adv.* **2019**, *9*, 33761–33774. https://doi:10.1039/c9ra05661k. |
| [4] | <https://www.cancer.gov/about-cancer/treatment/drugs/fluorouracil> |
| [5] | <https://www.cancer.gov/about-cancer/treatment/drugs/fluorouracil-topical> |
| [6] | <https://www.drugs.com/history/sutent.html> |
| [7] | <https://www.cancer.gov/about-cancer/treatment/drugs/sunitinibmalate> |
| [8] | Propidium Iodide Flow Cytometrykit for cycle analysis, ab139418 ([www.abcam.com](http://www.abcam.com)). |
| [9] | Annexin V-FITC Apoptosis Detection Kit (Catalog#K101-25), BioVision, CA 94043 USA ([www.biovision.com](http://www.biovision.com)). |
| [10] | SANTA CRUZ BIOTECHNOLOGY, INC., EGFR (528): sc-120 ([www.scbt.com](http://www.scbt.com)). |
| [11] | SANTA CRUZ BIOTECHNOLOGY, INC., VEGFR2 (A-3): sc-6251 ([www.scbt.com](http://www.scbt.com)). |
| [12] | Girgis, A. S.; Panda, S. S.; Srour, A. M.; Abdelnaser, A.; Nasr, S.; Moatasim, Y.; Kutkat, O.; El Taweel, A.; Kandeil, A.; Mostafa, A.; Ali, M. A.; Fawzy, N. G.; Bekheit, M. S.; Shalaby, E. M.; Gigli, L.; Fayad, W.; Soliman, A. A. F. 3-Alkenyl-2-oxindoles: Synthesis, antiproliferative and antiviral properties against SARS-CoV-2. *Bioorg. Chem.* **2021**, *114*, 105131. https://doi.org/10.1016/j.bioorg.2021.105131. |
| [13] | Srour, A. M.; Panda, S. S.; Mostafa, A.; Fayad, W.; El-Manawaty, M. A.; Soliman, A. A. F.; Moatasim, Y.; El Taweel, A.; Abdelhameed, M. F.; Bekheit, M. S.; Ali, M. A.; Girgis, A. S. Synthesis of aspirin-curcumin mimic conjugates of potential antitumor and anti-SARS-CoV-2 properties. *Bioorg. Chem.* **2021**, *117*, 105466. https://doi.org/10.1016/j.bioorg.2021.105466. |
| [14] | Seliem, I. A.; Girgis, A. S.; Moatasim, Y.; Kandeil, A.; Mostafa, A.; Ali, M. A.; Bekheit, M. S.; Panda, S. S. New pyrazine conjugates: Synthesis, computational studies, and antiviral properties against SARS-CoV-2. *ChemMedChem* **2021**, *16*, 3418–3427. https://doi.org/10.1002/cmdc.202100476. |
| [15] | Youssef, M. A.; Panda, S. S.; El-Shiekh, R. A.; Shalaby, E. M.; Aboshouk, D. R.; Fayad, W.; Fawzy, N. G.; Girgis, A. S. Synthesis and molecular modeling studies of cholinesterase inhibitor dispiro[indoline-3,2'-pyrrolidine-3',3''-pyrrolidines]. *RSC Adv.* **2020**, *10*, 21830–21838. https://doi:10.1039/d0ra03064c. |

**Table S1.** Crystal and refinement data of compounds **6b**, **6c**, **6d** and **6h**.

|  | **6b** | **6c** | **6d** | **6h** |
| --- | --- | --- | --- | --- |
| Molecular Formula | C_30_H_28_ClN_3_O_4_S | C_31_H_31_N_3_O_4_S | C_31_H_30_ClN_3_O_4_S | C_33_H_34_ClF_2_N_3_O_5_S |
| Fw | 562.06 | 541.65 | 576.09 | 658.14 |
| Temperature /K | 296(2) | 296(2) | 296(2) | 293(2) |
| λ / Å | 1.54184 | 1.54184 | 1.54184 | 1.54184 |
| Crystal system | Triclinic | Monoclinic | Monoclinic | Orthorhombic |
| Space group | P Ī | P2_1_/c | P2_1_/c | Pna2_1_ |
| a / Å | 10.7311(4) | 14.8318(2) | 15.0367(3) | 19.6747(3) |
| b / Å | 15.3491(6) | 16.4556(3) | 17.0841(4) | 9.3784(2) |
| c / Å | 17.6927(6) | 11.2040(2) | 11.2275(2) | 35.1660(8) |
| α / ° | 83.642(3) | 90 | 90 | 90 |
| b / ° | 74.346(3) | 91.596(2) | 95.197(2) | 90 |
| g / ° | 87.505(3) | 90 | 90 | 90 |
| Volume / Å3 | 2788.58(18) | 2733.46(8) | 2872.36(10) | 6488.7(2) |
| Z | 4 | 4 | 4 | 8 |
| Density(cal) / Mg/m3 | 1.339 | 1.316 | 1.332 | 1.347 |
| Absorption coefficient | 2.246 | 1.392 | 2.194 | 2.125 |
| F(000) | 1176 | 1144 | 1208 | 2752 |
| Crystal size / mm^3^ | 0.295x0.262x0.064 | 0.282x0.171x0.129 | 0.271x0.194x0.164 | 0.286x0.262x0.108 |
| Reflections collected | 27017 | 32056 | 28765 | 29835 |
| Independent reflections | 11590 | 5733 | 6016 | 11383 |
| R(int) | 0.0226 | 0.0279 | 0.0277 | 0.0229 |
| G-o-f | 1.024 | 1.018 | 1.053 | 1.040 |
| R1[I>2σ(I)] | 0.0456 | 0.0363 | 0.0448 | 0.0543 |
| wR2[I>2σ(I)] | 0.1234 | 0.0954 | 0.1276 | 0.1559 |
| Flack parameter |  |  |  | 0.017(7) |

**Table S2**. Torsion angles in the pyrrolidine-piperidine system. Structures **6b** and **9h** contain two independent molecules (Fig. shows the atom numbering used of the pyrrolidine and piperidine rings).


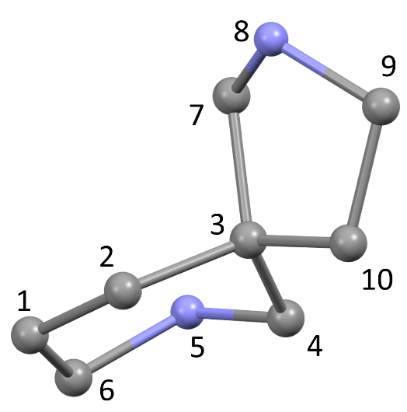


|  | **6b** | | **6c** | **6d** | **6h** | |
| --- | --- | --- | --- | --- | --- | --- |
| **3-4-5-6** | -65.52(17) | -71.13(18) | -70.04(14) | -67.87(19) | -61.0(11) | -59.2(5) |
| **4-5-6-1** | 42.3(2) | 52.4(2) | 52.42(16) | 48.9(2) | 17.7(13) | 12.3(6) |
| **5-6-1-2** | -21.4(2) | -25.3(2) | -29.34(18) | -27.2(2) | 19.8(9) | 26.2(6) |
| **6-1-2-3** | 23.2(2) | 16.0(2) | 24.26(18) | 24.7(2) | -12.4(6) | -16.5(7) |
| **1-2-3-4** | -41.10(18) | -29.92(19) | -36.59(15) | -38.27(19) | -29.3(5) | -28.9(6) |
| **2-3-4-5** | 61.41(16) | 55.98(18) | 57.43(13) | 57.97(17) | 63.4(6) | 64.3(5) |
| **3-7-8-9** | 38.06(17) | 40.57(18) | 41.89(12) | 40.78(16) | 42.3(4) | 41.9(4) |
| **7-8-9-10** | -47.18(17) | -43.48(19) | -43.44(13) | -43.19(18) | -39.5(4) | -42.6(4) |
| **8-9-10-3** | 35.69(17) | 26.95(19) | 25.52(13) | 26.44(18) | 19.0(4) | 24.3(4) |
| **9-10-3-7** | -13.05(16) | -3.12(17) | -0.82(12) | -2.36(16) | 5.9(4) | 0.4(4) |
| **10-3-7-8** | -13.93(15) | -21.50(16) | -23.77(11) | -22.24(15) | -28.2(4) | -24.6(4) |

**Table S3.** Descriptors of the BMLR-QSAR model for the synthesized agents against MCF7 (breast) cancer cell line.

| Entry | ID | Coefficient | *s* | *t* | Descriptor |
| --- | --- | --- | --- | --- | --- |
| 1 | 0 | -81.7387 | 11.781 | -6.938 | Intercept |
| 2 | *D*_1_ | 5164.31 | 241.69 | 21.368 | Max. nucleoph. react. index for atom O |
| 3 | *D*_2_ | 936.267 | 165.994 | 5.640 | Square root of partial surface area for atom C |
| *N* = 14, *n* = 2, *R*^2^ = 0.977, *R*^2^cvOO = 0.960, *R*^2^cvMO = 0.956, *F* = 236.532, *s*^2^ = 5.515  IC_50_ (*μ*M) = -81.7387 + (5164.31 x *D*_1_) + (936.267 x *D*_2_) | | | | | |

**Table S4.** Observed and estimated antiproliferation properties for the synthesized agents against MCF7 (breast) cancer cell line according to the BMLR-QSAR model.

| Entry | Compd. | Observed IC_50_, *μ*M | Estimated IC_50_, *μ*M | Error* |
| --- | --- | --- | --- | --- |
| 1 | **6a** | 19.787 | 19.024 | 0.763 |
| 2 | **6b** | 7.660 | 7.314 | 0.346 |
| 3 | **6d** | 6.915 | 9.439 | -2.524 |
| 4 | **6e** | 15.532 | 13.182 | 2.351 |
| 5 | **6f** | 5.000 | 5.668 | -0.668 |
| 6 | **6g** | 10.319 | 12.740 | -2.421 |
| 7 | **6h** | 4.694 | 4.162 | 0.532 |
| 8 | **6i** | 5.014 | 8.398 | -3.384 |
| 9 | **6j** | 4.514 | 0.922 | 3.592 |
| 10 | **6k** | 4.375 | 6.202 | -1.827 |
| 11 | **6l** | 3.986 | 1.387 | 2.599 |
| 12 | **6m** | 3.597 | 3.681 | -0.084 |
| 13 | **6n** | 40.213 | 41.690 | -1.477 |
| 14 | **6o** | 48.936 | 46.734 | 2.202 |

*Error is the difference between the observed and estimated property (IC_50_, *μ*M).

**Table S5.** Molecular descriptor values of the BMLR-QSAR model for the synthesized agents against MCF7 (breast) cancer cell line.

| Entry | Compd. | Descriptors* | |
| --- | --- | --- | --- |
|  |  | *D*_1_ | *D*_2_ |
| 1 | **6a** | 0.00554 | 0.07704 |
| 2 | **6b** | 0.0042 | 0.07192 |
| 3 | **6d** | 0.0042 | 0.07422 |
| 4 | **6e** | 0.00496 | 0.07402 |
| 5 | **6f** | 0.00404 | 0.07106 |
| 6 | **6g** | 0.00507 | 0.07293 |
| 7 | **6h** | 0.00394 | 0.07002 |
| 8 | **6i** | 0.00562 | 0.06525 |
| 9 | **6j** | 0.00385 | 0.06703 |
| 10 | **6k** | 0.0052 | 0.06523 |
| 11 | **6l** | 0.00436 | 0.06474 |
| 12 | **6m** | 0.00465 | 0.06558 |
| 13 | **6n** | 0.01155 | 0.0681 |
| 14 | **6o** | 0.01224 | 0.06968 |

**D*_1_ = Max. nucleoph. react. index for atom O, *D*_2_ = Square root of partial surface area for atom C.

**Table S6.** Descriptors of the BMLR-QSAR model for the synthesized agents against HCT116 (colon) cancer cell line.

| Entry | ID | Coefficient | *s* | *t* | Descriptor |
| --- | --- | --- | --- | --- | --- |
| 1 | 0 | -4854.93 | 781.82 | -6.210 | Intercept |
| 2 | *D*_1_ | 28.8351 | 4.656 | 6.194 | Max. n-n repulsion for bond C-N |
| 3 | *D*_2_ | 30.3734 | 7.045 | 4.311 | HA dependent HDCA-2 (Zefirov PC) |
| *N* = 15, *n* = 2, *R*^2^ = 0.834, *R*^2^cvOO = 0.709, *R*^2^cvMO = 0.742, *F* = 30.083, *s*^2^ = 9.022  IC_50_ (*μ*M) = -4854.93 + (28.8351 x *D*_1_) + (30.3734 x *D*_2_) | | | | | |

**Table S7.** Observed and estimated antiproliferation properties for the synthesized agents against HCT116 (colon) cancer cell line according to the BMLR-QSAR model.

| Entry | Compd. | Observed IC_50_, *μ*M | Estimated IC_50_, *μ*M | Error* |
| --- | --- | --- | --- | --- |
| 1 | **6a** | 15.957 | 10.119 | 5.838 |
| 2 | **6b** | 6.915 | 9.085 | -2.170 |
| 3 | **6c** | 6.125 | 11.816 | -5.691 |
| 4 | **6d** | 5.181 | 5.315 | -0.134 |
| 5 | **6e** | 9.894 | 9.394 | 0.500 |
| 6 | **6f** | 5.431 | 7.426 | -1.995 |
| 7 | **6g** | 4.944 | 3.038 | 1.906 |
| 8 | **6h** | 4.597 | 4.833 | -0.236 |
| 9 | **6i** | 5.472 | 7.130 | -1.658 |
| 10 | **6j** | 4.722 | 2.153 | 2.569 |
| 11 | **6k** | 4.167 | 2.688 | 1.479 |
| 12 | **6l** | 4.111 | 7.523 | -3.412 |
| 13 | **6m** | 3.236 | 2.510 | 0.726 |
| 14 | **6n** | 15.426 | 15.485 | -0.059 |
| 15 | **6o** | 28.511 | 26.176 | 2.335 |

*Error is the difference between the observed and estimated property (IC_50_, *μ*M).

**Table S8.** Molecular descriptor values of the BMLR-QSAR model for the synthesized agents against HCT116 (colon) cancer cell line.

| Entry | Compd. | Descriptors* | |
| --- | --- | --- | --- |
|  |  | *D*_1_ | *D*_2_ |
| 1 | **6a** | 168.0659 | 0.62063 |
| 2 | **6b** | 167.8616 | 0.78052 |
| 3 | **6c** | 168.1822 | 0.56608 |
| 4 | **6d** | 168.0568 | 0.47108 |
| 5 | **6e** | 168.0749 | 0.58821 |
| 6 | **6f** | 168.0006 | 0.59394 |
| 7 | **6g** | 167.8547 | 0.58799 |
| 8 | **6h** | 167.8383 | 0.66266 |
| 9 | **6i** | 167.8566 | 0.72089 |
| 10 | **6j** | 167.7668 | 0.64231 |
| 11 | **6k** | 167.964 | 0.47269 |
| 12 | **6l** | 167.9743 | 0.6221 |
| 13 | **6m** | 167.8045 | 0.61825 |
| 14 | **6n** | 167.9705 | 0.88784 |
| 15 | **6o** | 168.4408 | 0.79336 |

**D*_1_ = Max. n-n repulsion for bond C-N, *D*_2_ = HA dependent HDCA-2 (Zefirov PC).

**Table S9.** Descriptors of the BMLR-QSAR model for the synthesized agents against A431 (skin squamous) cancer cell line.

| Entry | ID | Coefficient | *s* | *t* | Descriptor |
| --- | --- | --- | --- | --- | --- |
| 1 | 0 | 9.80268 | 1.467 | 6.684 | Intercept |
| 2 | *D*_1_ | 2.06853 | 0.223 | 9.256 | FPSA2 Fractional PPSA (PPSA-2/TMSA) (Zefirov PC) |
| 3 | *D*_2_ | -2.14585 | 0.312 | -6.874 | Average information content (order 2) |
| *N* = 15, *n* = 2, *R*^2^ = 0.898, *R*^2^cvOO = 0.800, *R*^2^cvMO = 0.816, *F* = 52.681, *s*^2^ = 0.023  Log(IC_50_, *μ*M) = 9.80268 + (2.06853 x *D*_1_) ‒ (2.14585 x *D*_2_) | | | | | |

**Table S10.** Observed and estimated antiproliferation properties for the synthesized agents against A431 (skin squamous) cancer cell line according to the BMLR-QSAR model.

| Entry | Compd. | Observed log(IC_50_, *μ*M) | Observed IC_50_, *μ*M | Estimated log(IC_50_, *μ*M) | Estimated IC_50_, *μ*M | Error* |
| --- | --- | --- | --- | --- | --- | --- |
| 1 | **6a** | 1.50974 | 32.34 | 1.45536 | 28.534 | 3.806 |
| 2 | **6b** | 0.961374 | 9.149 | 0.965821 | 9.243 | -0.094 |
| 3 | **6c** | 1.52102 | 33.191 | 1.37714 | 23.831 | 9.360 |
| 4 | **6d** | 0.695307 | 4.958 | 0.784246 | 6.085 | -1.127 |
| 5 | **6e** | 1.20585 | 16.064 | 1.01226 | 10.286 | 5.778 |
| 6 | **6f** | 0.677972 | 4.764 | 0.641793 | 4.383 | 0.381 |
| 7 | **6g** | 0.790074 | 6.167 | 0.90192 | 7.978 | -1.811 |
| 8 | **6h** | 0.781181 | 6.042 | 0.594358 | 3.930 | 2.112 |
| 9 | **6i** | 0.643749 | 4.403 | 0.673033 | 4.710 | -0.307 |
| 10 | **6j** | 0.610979 | 4.083 | 0.647407 | 4.440 | -0.357 |
| 11 | **6k** | 0.472171 | 2.966 | 0.556076 | 3.598 | -0.632 |
| 12 | **6l** | 0.567497 | 3.694 | 0.481003 | 3.027 | 0.667 |
| 13 | **6m** | 0.386321 | 2.434 | 0.620439 | 4.173 | -1.739 |
| 14 | **6n** | 1.54275 | 34.894 | 1.79264 | 62.035 | -27.141 |
| 15 | **6o** | 1.65722 | 45.417 | 1.51972 | 33.092 | 12.325 |

*Error is the difference between the observed and estimated property (IC_50_, *μ*M).

**Table S11.** Molecular descriptor values of the BMLR-QSAR model for the synthesized agents against A431 (skin squamous) cancer cell line.

| Entry | Compd. | Descriptors* | |
| --- | --- | --- | --- |
|  |  | *D*_1_ | *D*_2_ |
| 1 | **6a** | 0.62291 | 4.49045 |
| 2 | **6b** | 0.64083 | 4.73586 |
| 3 | **6c** | 0.69107 | 4.5926 |
| 4 | **6d** | 0.64811 | 4.82749 |
| 5 | **6e** | 0.72276 | 4.79319 |
| 6 | **6f** | 0.69417 | 4.93828 |
| 7 | **6g** | 0.76192 | 4.88237 |
| 8 | **6h** | 0.75729 | 5.02123 |
| 9 | **6i** | 0.55876 | 4.79319 |
| 10 | **6j** | 0.55568 | 4.80216 |
| 11 | **6k** | 0.59473 | 4.88237 |
| 12 | **6l** | 0.56735 | 4.89096 |
| 13 | **6m** | 0.53334 | 4.79319 |
| 14 | **6n** | 1.14432 | 4.8359 |
| 15 | **6o** | 1.121 | 4.9406 |

**D*_1_ = FPSA2 Fractional PPSA (PPSA-2/TMSA) (Zefirov PC), *D*_2_ = Average information content (order 2).

**Table S12.** Descriptors of the BMLR-QSAR model for the synthesized agents against PaCa2 (pancreatic) cancer cell line.

| Entry | ID | Coefficient | *s* | *t* | Descriptor |
| --- | --- | --- | --- | --- | --- |
| 1 | 0 | 1386.05 | 91.533 | 15.143 | Intercept |
| 2 | *D*_1_ | -9.48172 | 1.713 | -5.537 | Rot. entropy (300K) |
| 3 | *D*_2_ | -510.133 | 43.643 | -11.689 | Max. atomic orbital electronic population |
| *N* = 13, *n* = 2, *R*^2^ = 0.9573, *R*^2^cvOO = 0.938, *R*^2^cvMO = 0.944, *F* = 112.03, *s*^2^ = 8.886  IC_50_ (*μ*M) = 1386.05 ‒ (9.48172 x *D*_1_) ‒ (510.133 x *D*_2_) | | | | | |

**Table S13.** Observed and estimated antiproliferation properties for the synthesized agents against PaCa2 (pancreatic) cancer cell line according to the BMLR-QSAR model.

| Entry | Compd. | Observed IC_50_, *μ*M | Estimated IC_50_, *μ*M | Error* |
| --- | --- | --- | --- | --- |
| 1 | **6a** | 48.404 | 47.054 | 1.350 |
| 2 | **6b** | 20.638 | 19.666 | 0.972 |
| 3 | **6d** | 13.085 | 16.974 | -3.889 |
| 4 | **6e** | 39.894 | 41.660 | -1.766 |
| 5 | **6f** | 11.702 | 12.546 | -0.844 |
| 6 | **6g** | 28.404 | 22.850 | 5.554 |
| 7 | **6h** | 14.043 | 13.631 | 0.412 |
| 8 | **6i** | 9.043 | 12.067 | -3.024 |
| 9 | **6j** | 8.83 | 7.488 | 1.342 |
| 10 | **6k** | 8.83 | 11.518 | -2.688 |
| 11 | **6l** | 11.915 | 7.824 | 4.091 |
| 12 | **6m** | 12.5 | 13.047 | -0.547 |
| 13 | **6n** | 32.766 | 33.729 | -0.963 |

*Error is the difference between the observed and estimated property (IC_50_, *μ*M).

**Table S14.** Molecular descriptor values of the BMLR-QSAR model for the synthesized agents against PaCa2 (pancreatic) cancer cell line.

| Entry | Compd. | Descriptors* | |
| --- | --- | --- | --- |
|  |  | *D*_1_ | *D*_2_ |
| 1 | **6a** | 37.155 | 1.93421 |
| 2 | **6b** | 37.475 | 1.98195 |
| 3 | **6d** | 37.76 | 1.98193 |
| 4 | **6e** | 37.732 | 1.93406 |
| 5 | **6f** | 38.227 | 1.98193 |
| 6 | **6g** | 37.908 | 1.96766 |
| 7 | **6h** | 38.112 | 1.98194 |
| 8 | **6i** | 38.277 | 1.98194 |
| 9 | **6j** | 38.577 | 1.98534 |
| 10 | **6k** | 38.336 | 1.98192 |
| 11 | **6l** | 38.725 | 1.98193 |
| 12 | **6m** | 39.007 | 1.96645 |
| 13 | **6n** | 38.569 | 1.93405 |

**D*_1_ = Rot. entropy (300K), *D*_2_ = Max. atomic orbital electronic population.

**Table S15.** Descriptors of the BMLR-QSAR model for the synthesized agents against SARS-CoV-2.

| Entry | ID | Coefficient | *s* | *t* | Descriptor |
| --- | --- | --- | --- | --- | --- |
| 1 | 0 | 1478.94 | 147.319 | 10.039 | Intercept |
| 2 | *D*_1_ | 2.66577 | 0.386 | 6.915 | LUMO+1 energy |
| 3 | *D*_2_ | -120.804 | 30.035 | -4.022 | Max. electroph. react. index for atom O |
| 4 | *D*_3_ | -7.98889 | 0.796 | -10.031 | Min. atomic state energy for atom N |
| *N* = 15, *n* = 3, *R*^2^ = 0.917, *R*^2^cvOO = 0.863, *R*^2^cvMO = 0.883, *F* = 40.481, *s*^2^ = 0.023  Log(IC_50_, *μ*M) = 1478.94 + (2.66577 x *D*_1_) ‒ (120.804 x *D*_2_) ‒ (7.98889 x *D*_3_) | | | | | |

**Table S16.** Observed and estimated properties for the synthesized agents against SARS-CoV-2 according to the BMLR-QSAR model.

| Entry | Compd. | Observed log(IC_50_, *μ*M) | Observed IC_50_, *μ*M | Estimated log(IC_50_, *μ*M) | Estimated IC_50_, *μ*M | Error* |
| --- | --- | --- | --- | --- | --- | --- |
| 1 | **6a** | 1.53479 | 34.26 | 1.44701 | 27.990 | 6.270 |
| 2 | **6b** | 0.983536 | 9.628 | 0.983712 | 9.632 | -0.004 |
| 3 | **6c** | 2.01115 | 102.6 | 1.83924 | 69.062 | 33.538 |
| 4 | **6d** | 2.23376 | 171.3 | 2.14503 | 139.646 | 31.654 |
| 5 | **6e** | 1.44483 | 27.85 | 1.36011 | 22.914 | 4.936 |
| 6 | **6f** | 0.884569 | 7.666 | 1.04671 | 11.136 | -3.470 |
| 7 | **6g** | 1.22814 | 16.91 | 1.06752 | 11.682 | 5.228 |
| 8 | **6h** | 0.885757 | 7.687 | 0.884726 | 7.669 | 0.018 |
| 9 | **6i** | 2.05423 | 113.3 | 1.93887 | 86.870 | 26.430 |
| 10 | **6j** | 1.43281 | 27.09 | 1.36588 | 23.221 | 3.869 |
| 11 | **6k** | 0.925879 | 8.431 | 1.00626 | 10.145 | -1.714 |
| 12 | **6l** | 1.49762 | 31.45 | 1.67282 | 47.078 | -15.628 |
| 13 | **6m** | 0.95056 | 8.924 | 0.935127 | 8.612 | 0.312 |
| 14 | **6n** | 1.55497 | 35.89 | 1.83817 | 68.892 | -33.002 |
| 15 | **6o** | 1.94571 | 88.25 | 2.03714 | 108.928 | -20.678 |

*Error is the difference between the observed and estimated property (IC_50_, *μ*M).

**Table S17.** Molecular descriptor values of the BMLR-QSAR model for the synthesized agents against SARS-CoV-2.

| Entry | Compd. | Descriptors* | | |
| --- | --- | --- | --- | --- |
|  |  | *D*_1_ | *D*_2_ | *D*_3_ |
| 1 | **6a** | -0.44 | 0.0117 | 184.6198 |
| 2 | **6b** | -0.555 | 0.009 | 184.6803 |
| 3 | **6c** | -0.412 | 0.01264 | 184.5658 |
| 4 | **6d** | -0.495 | 0.01192 | 184.5108 |
| 5 | **6e** | -0.902 | 0.00572 | 184.567 |
| 6 | **6f** | -0.599 | 0.00912 | 184.6559 |
| 7 | **6g** | -0.841 | 0.00681 | 184.6074 |
| 8 | **6h** | -0.603 | 0.0106 | 184.6524 |
| 9 | **6i** | -0.552 | 0.00977 | 184.55 |
| 10 | **6j** | -0.825 | 0.01114 | 184.5099 |
| 11 | **6k** | -0.752 | 0.01163 | 184.5719 |
| 12 | **6l** | -0.797 | 0.00873 | 184.5173 |
| 13 | **6m** | -0.605 | 0.00681 | 184.7028 |
| 14 | **6n** | -0.497 | 0.01058 | 184.5687 |
| 15 | **6o** | -0.609 | 0.00967 | 184.5203 |

**D*_1_ = LUMO+1 energy, *D*_2_ = Max. electroph. react. index for atom O, *D*_3_ = Min. atomic state energy for atom N.


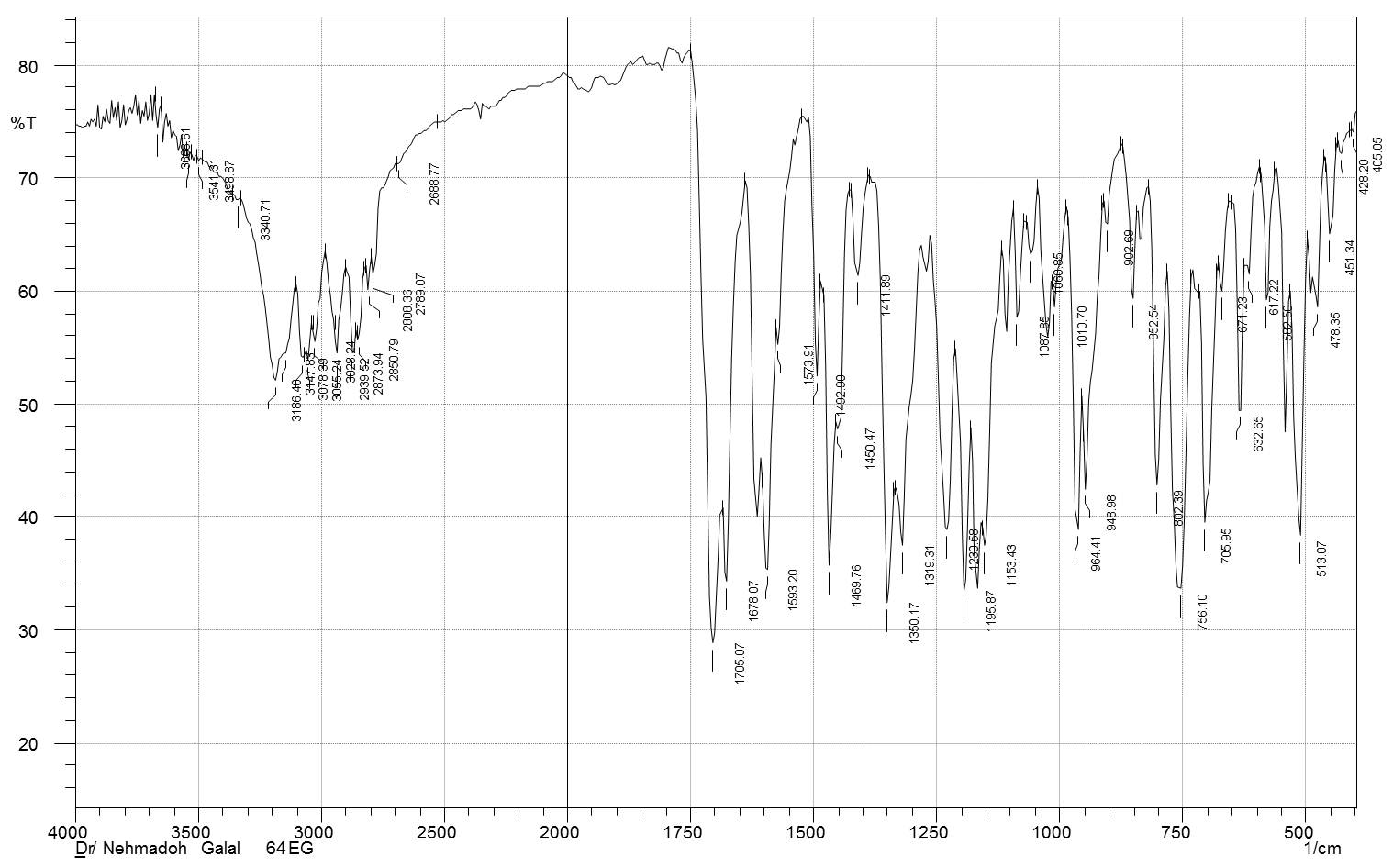


**Fig. S1.** IR spectrum of compound **6a** (KBr pellet).

**
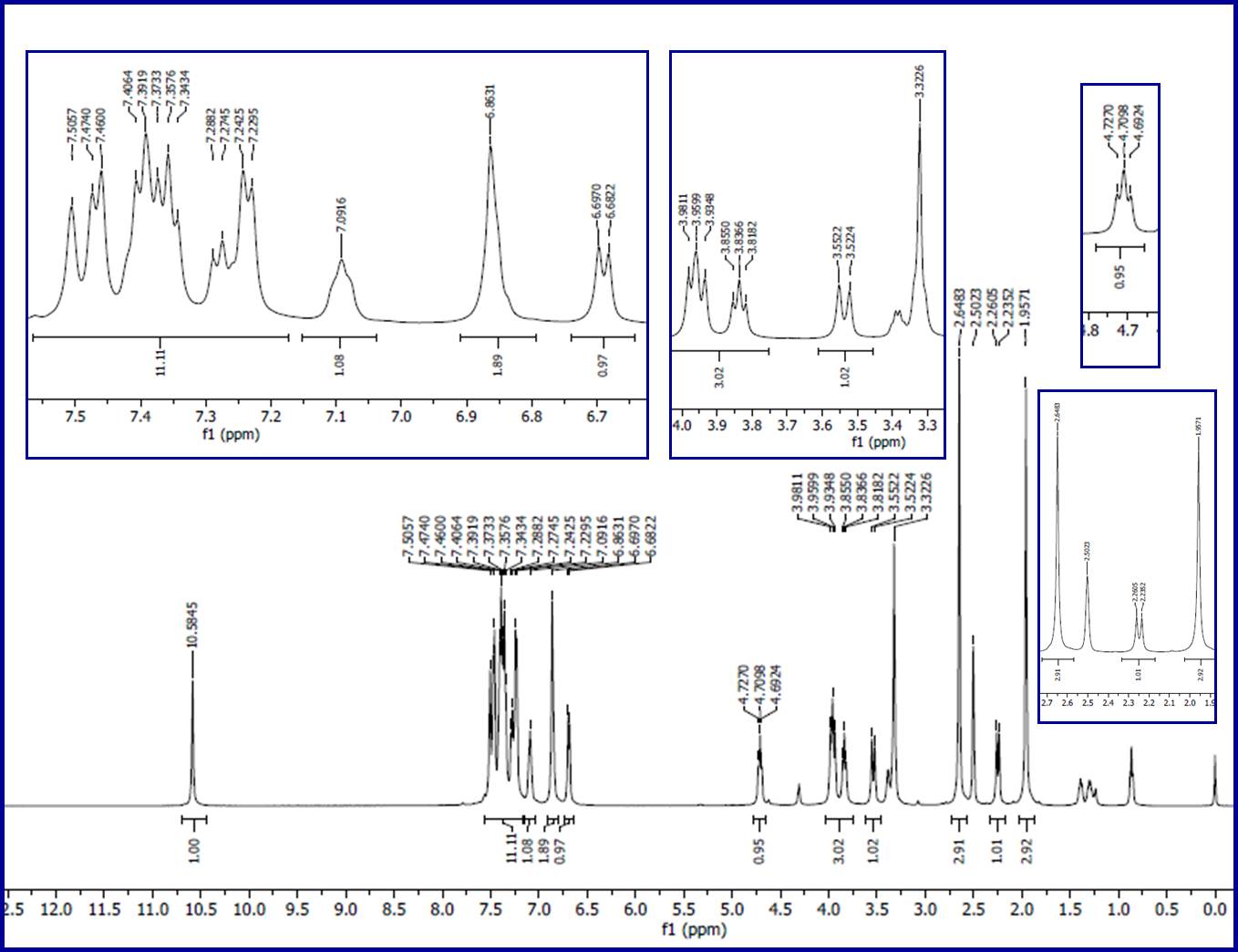
**

**Fig. S2.** ^1^H-NMR spectrum of compound **6a** in DMSO-*d_6_*.

**
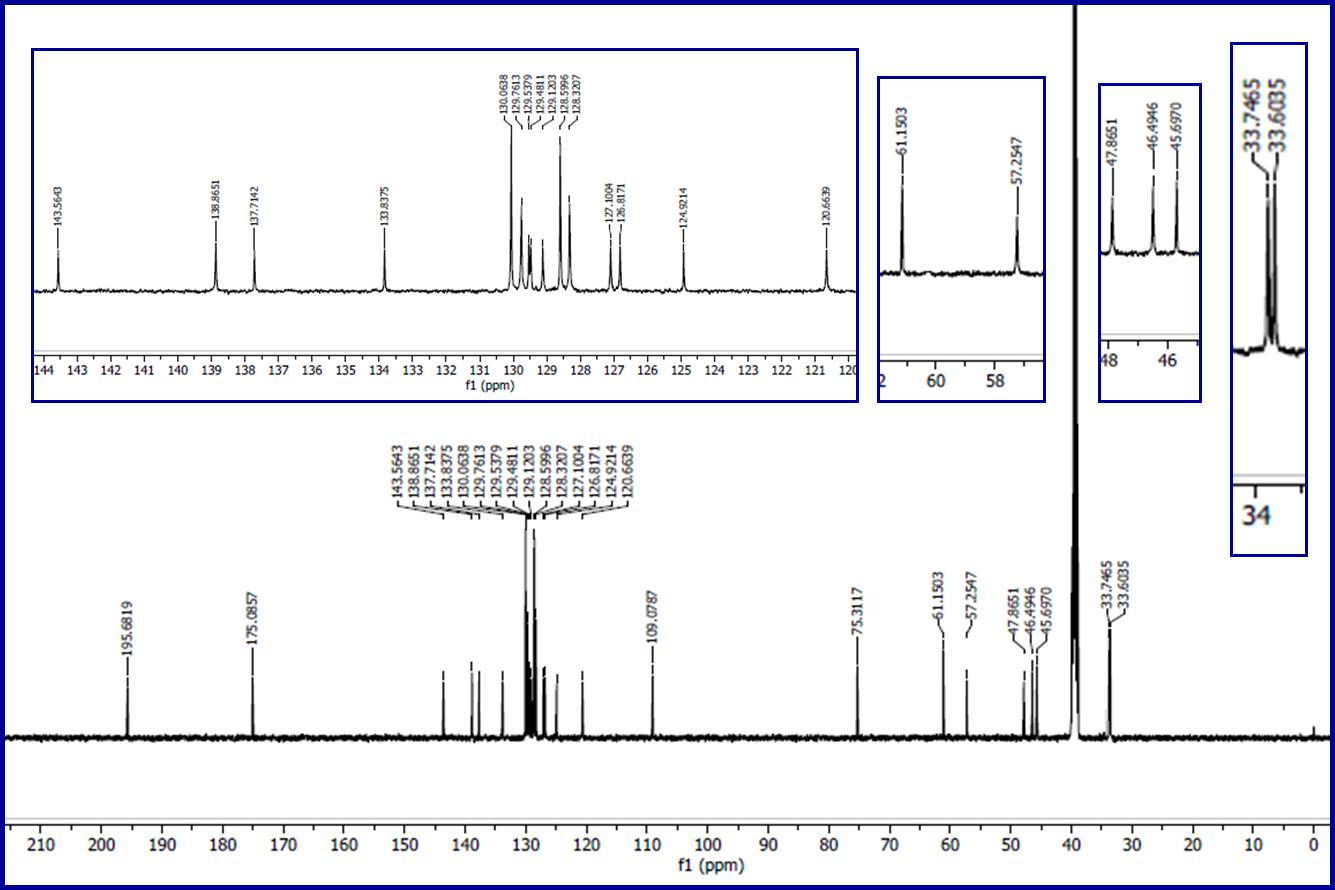
**

**Fig. S3.** ^13^C-NMR spectrum of compound **6a** in DMSO-*d_6_*.


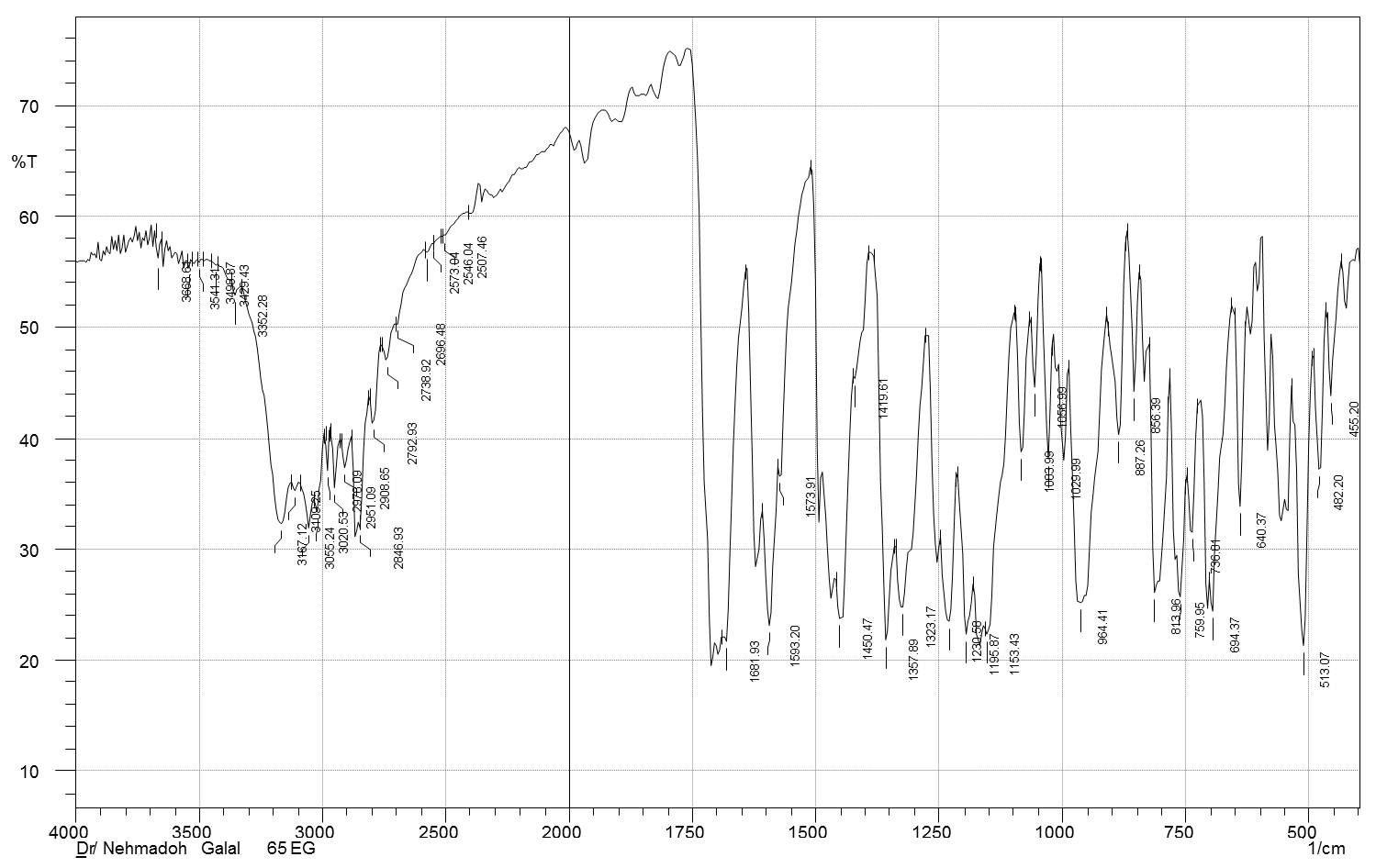


**Fig. S4.** IR spectrum of compound **6b** (KBr pellet).


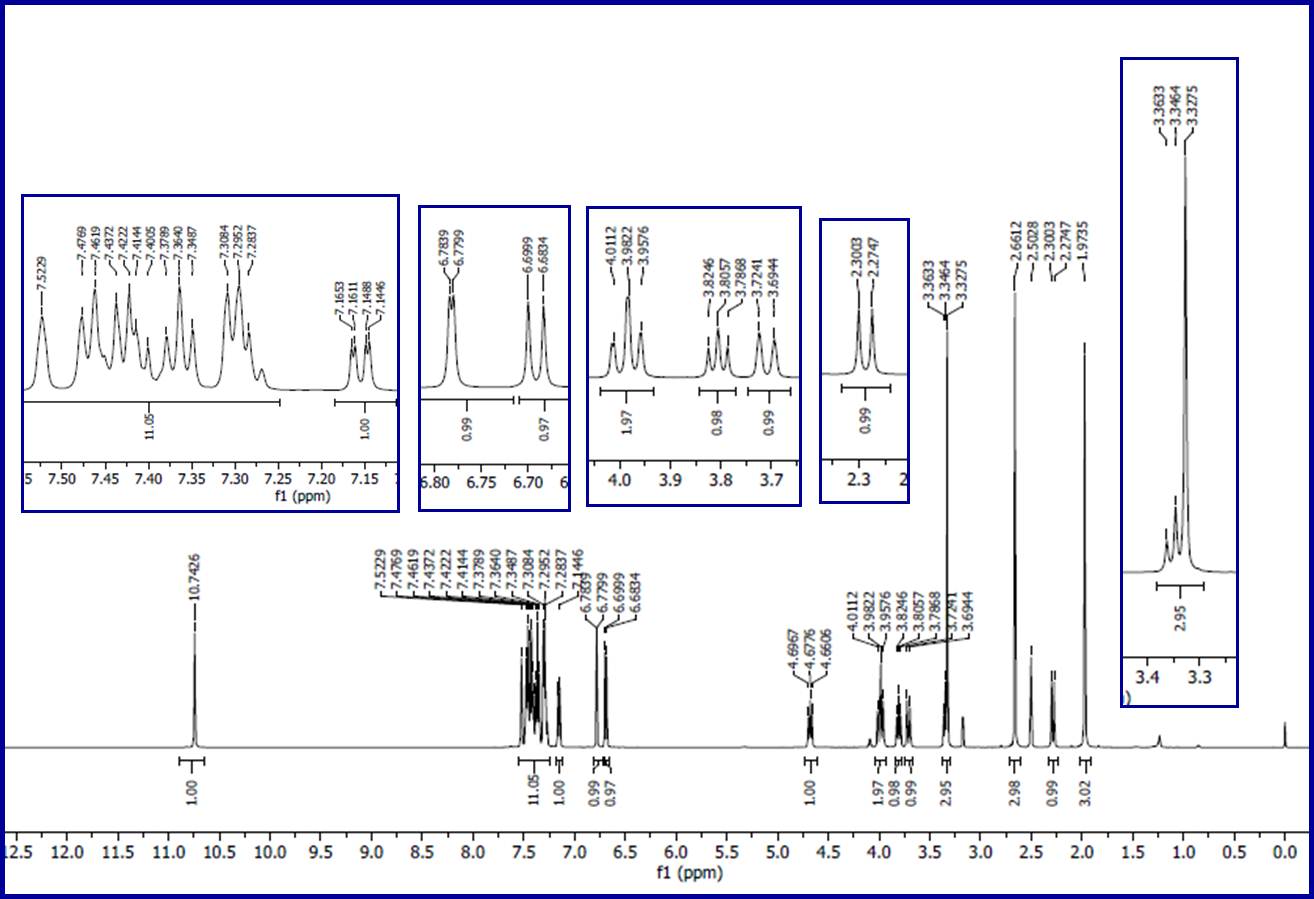


**Fig. S5.** ^1^H-NMR spectrum of compound **6b** in DMSO-*d_6_*.


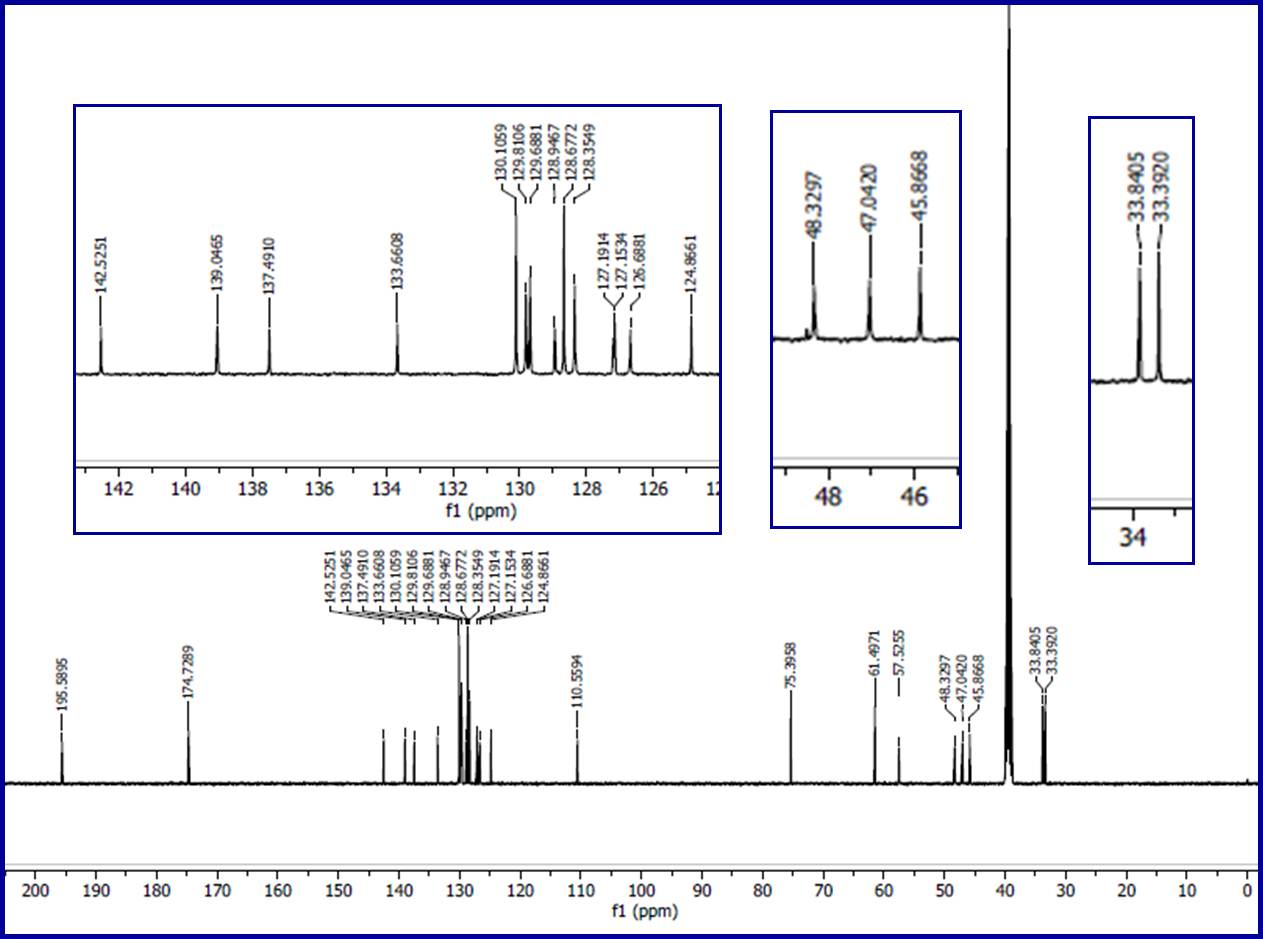


**Fig. S6.** ^13^C-NMR spectrum of compound **6b** in DMSO-*d_6_*.


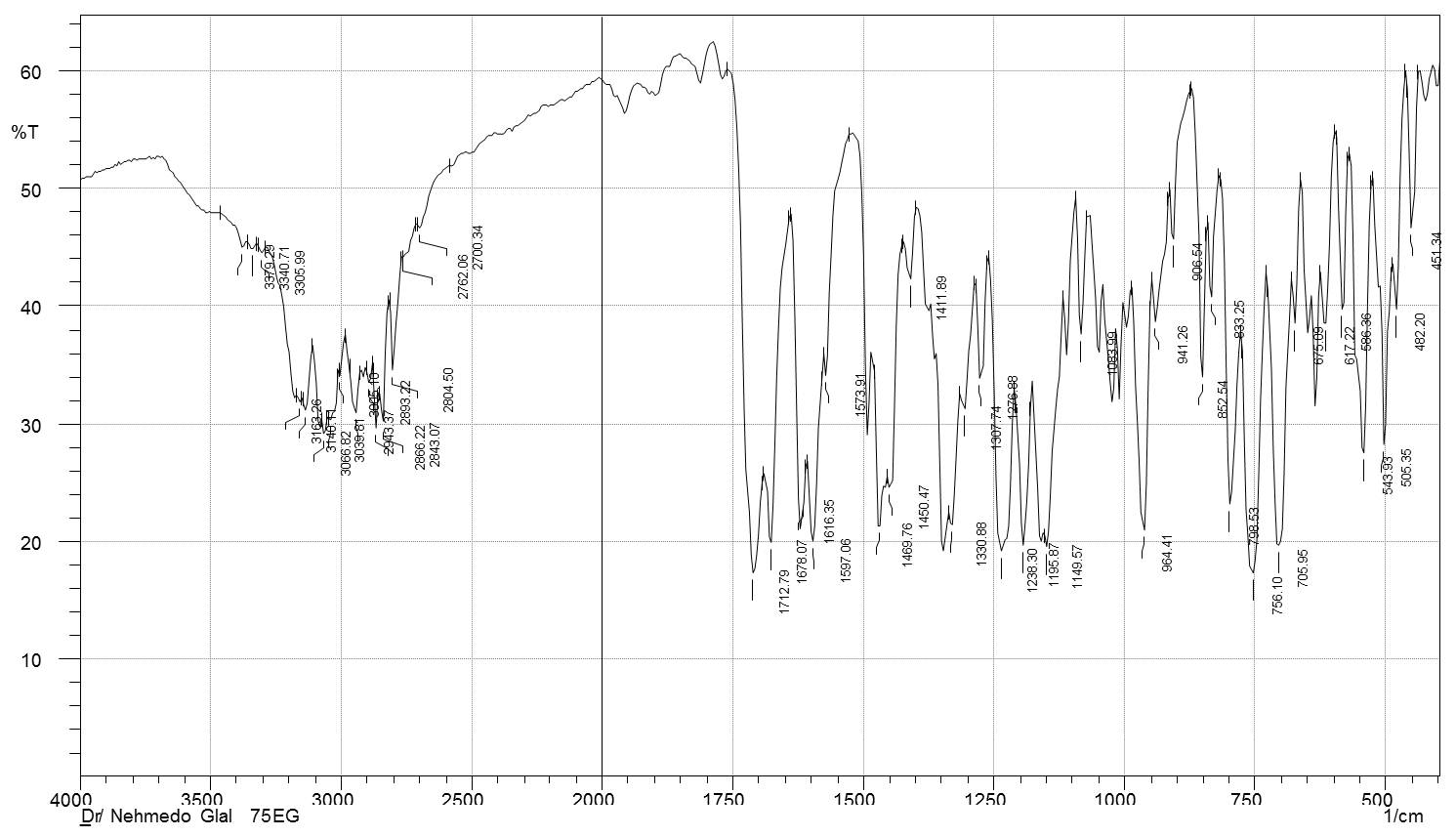


**Fig. S7.** IR spectrum of compound **6c** (KBr pellet).


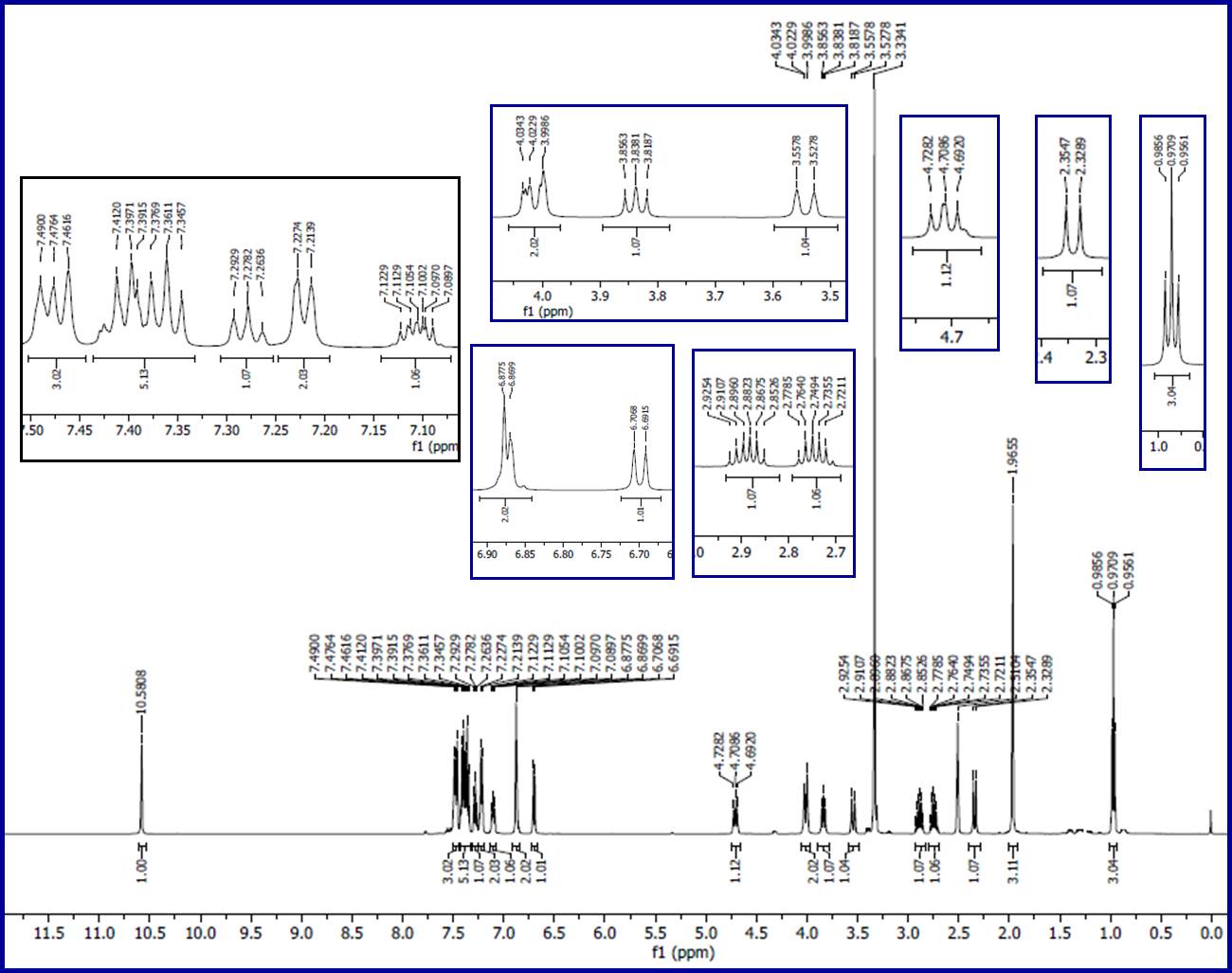


**Fig. S8.** ^1^H-NMR spectrum of compound **6c** in DMSO-*d_6_*.


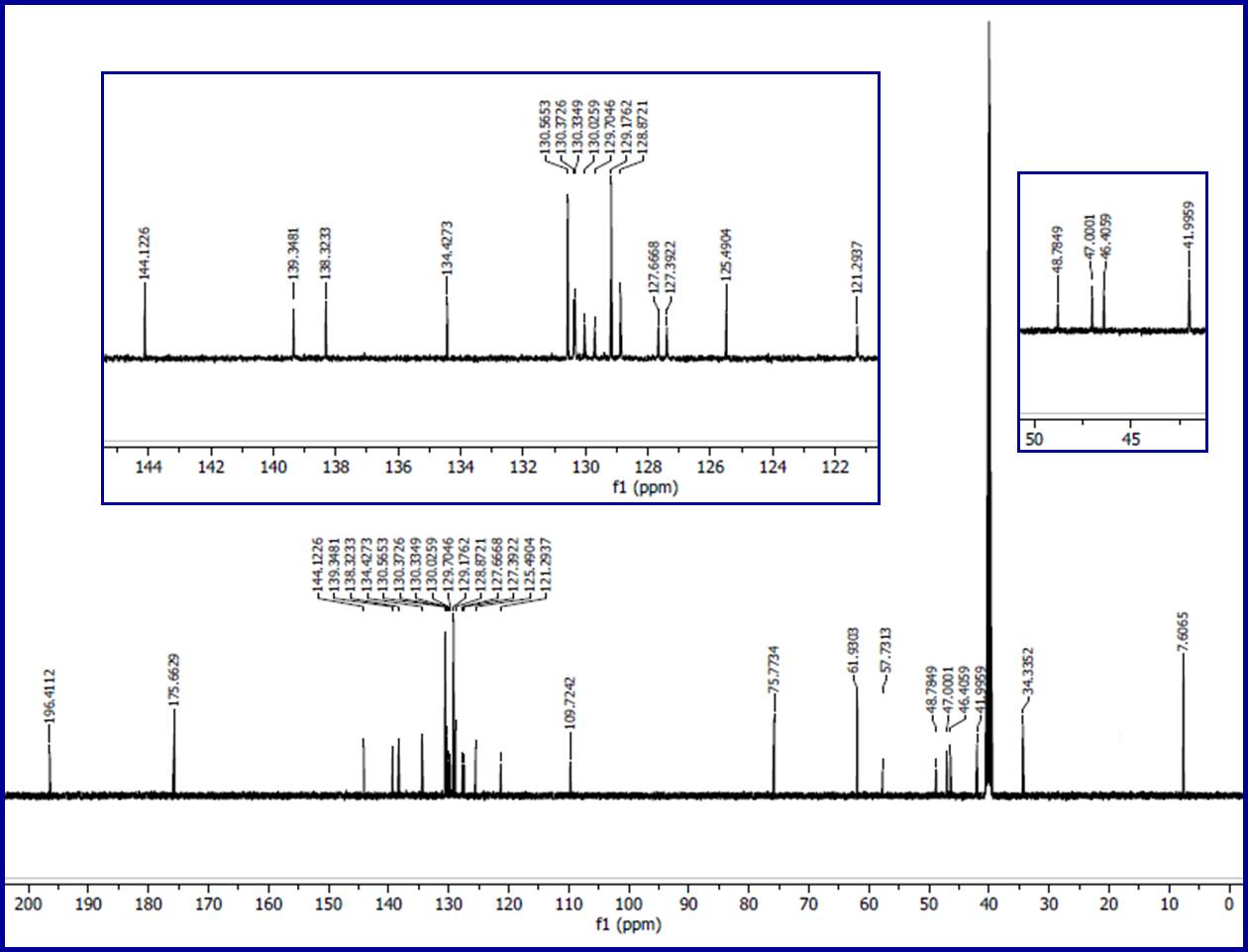


**Fig. S9.** ^13^C-NMR spectrum of compound **6c** in DMSO-*d_6_*.


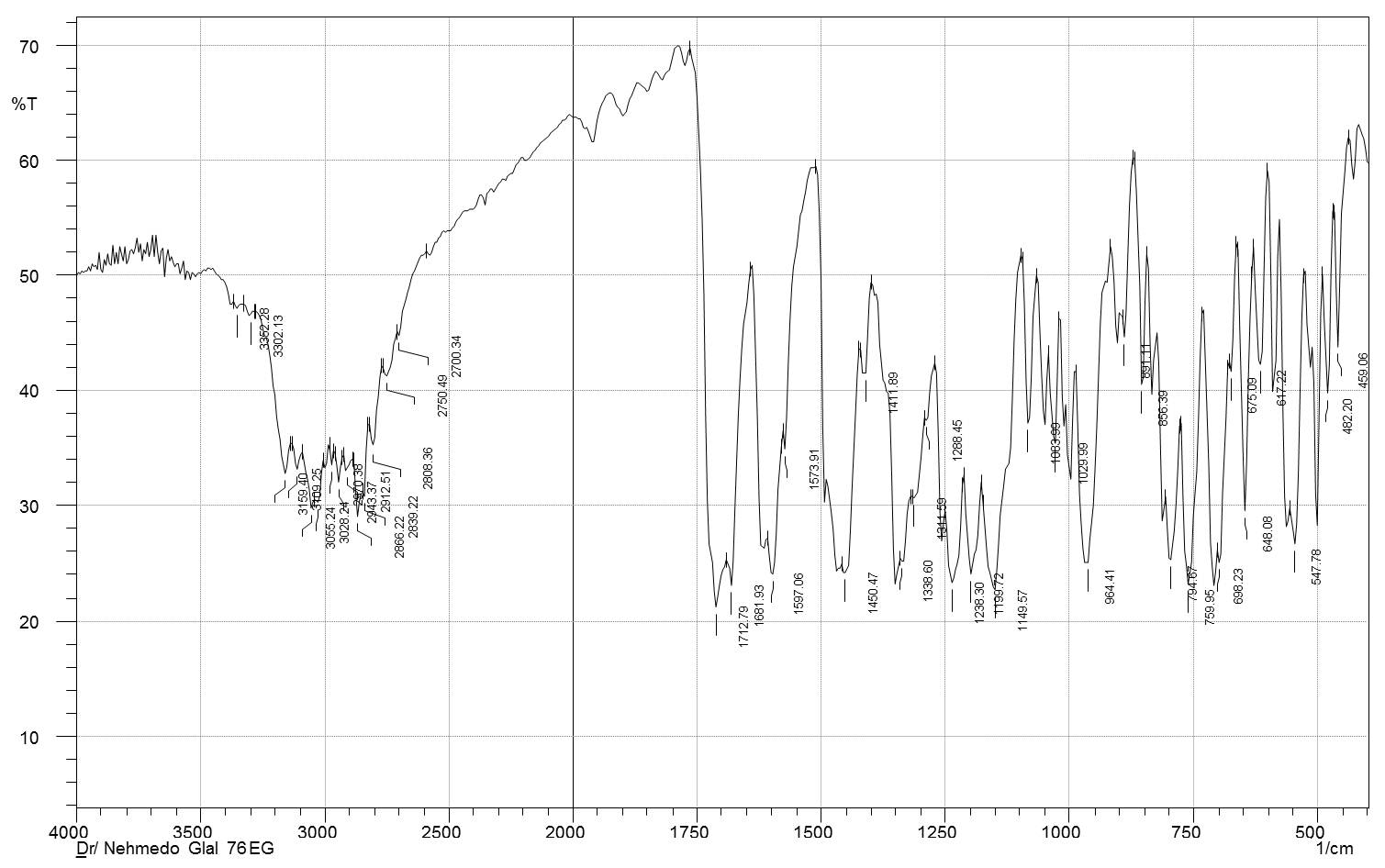


**Fig. S10.** IR spectrum of compound **6d** (KBr pellet).


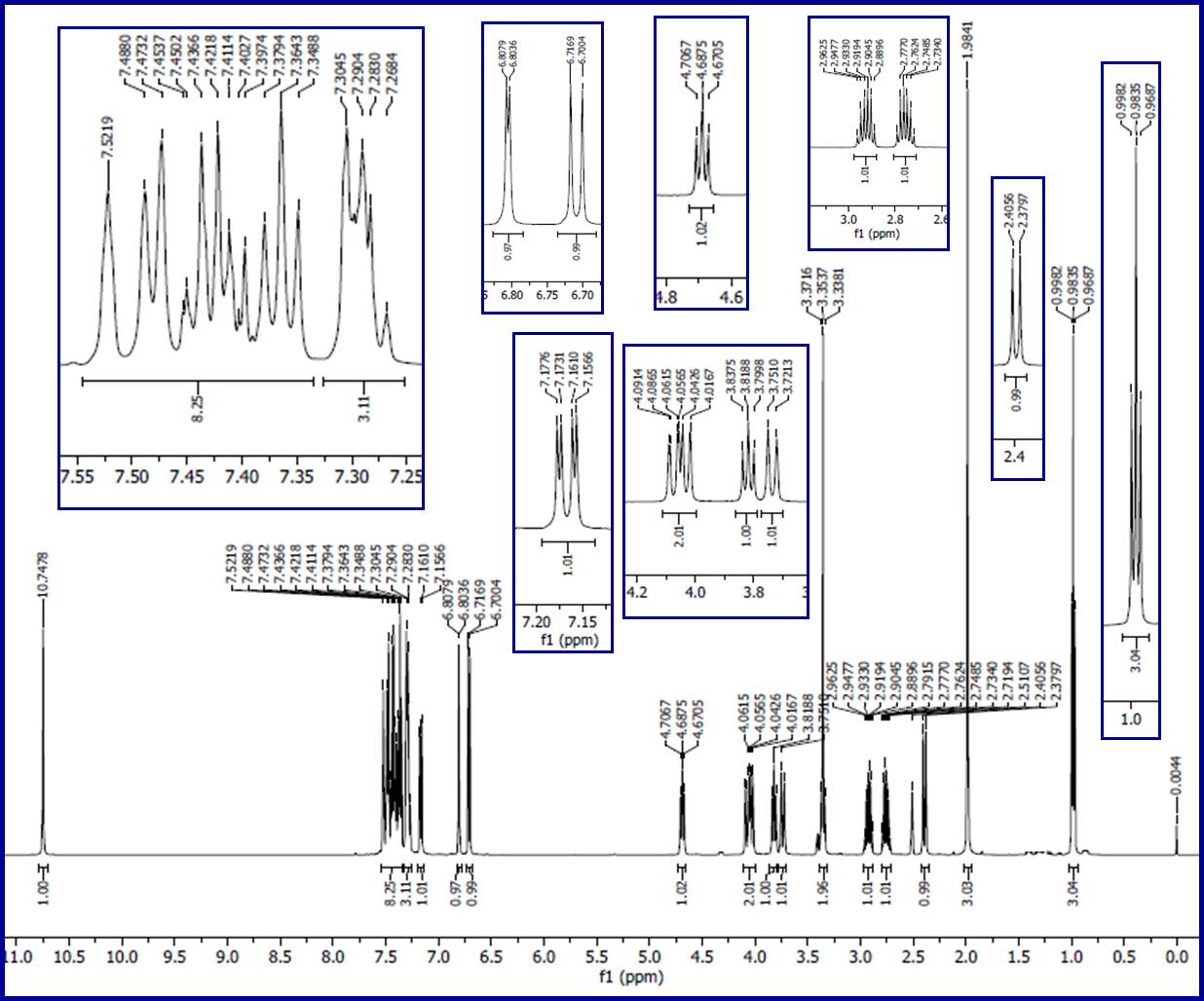


**Fig. S11.** ^1^H-NMR spectrum of compound **6d** in DMSO-*d_6_*.


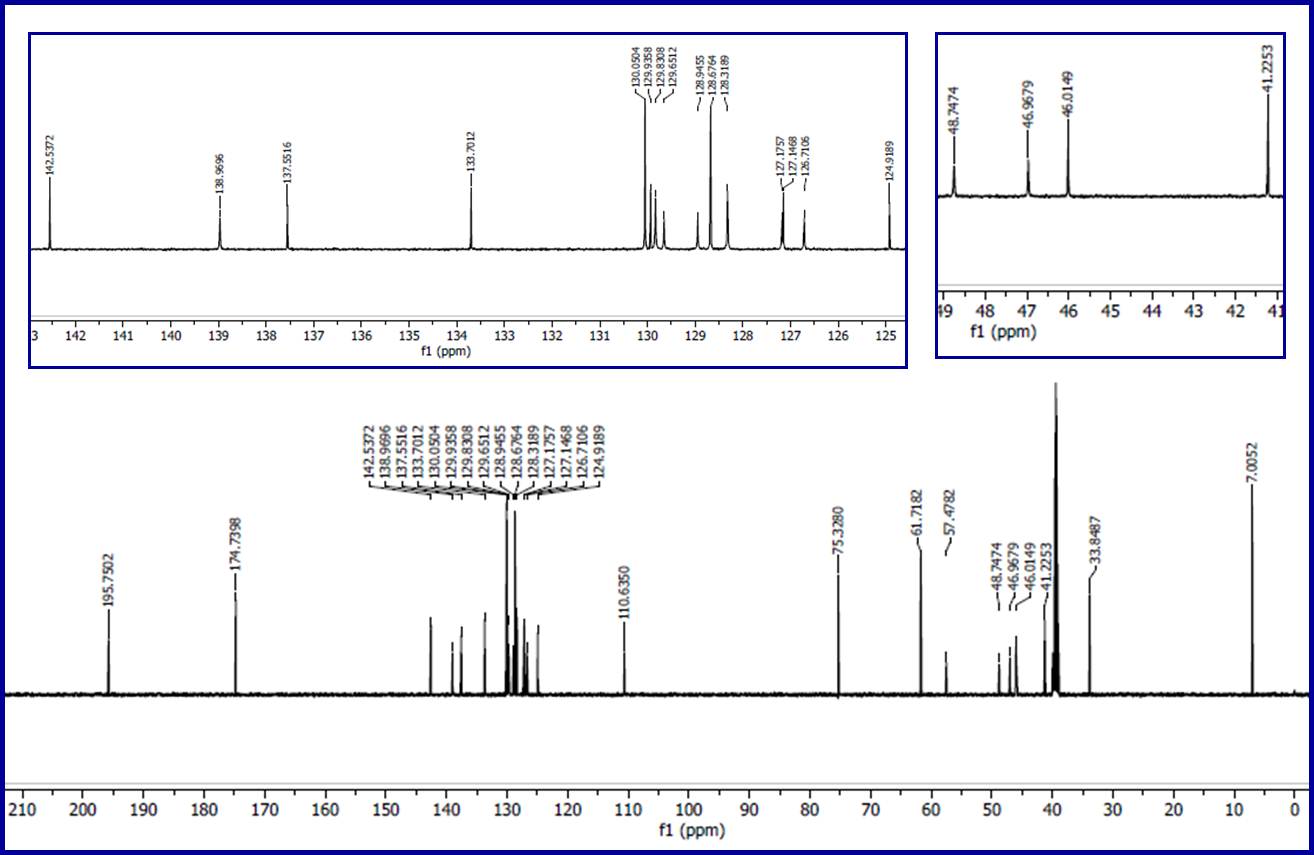


**Fig. S12.** ^13^C-NMR spectrum of compound **6d** in DMSO-*d_6_*.


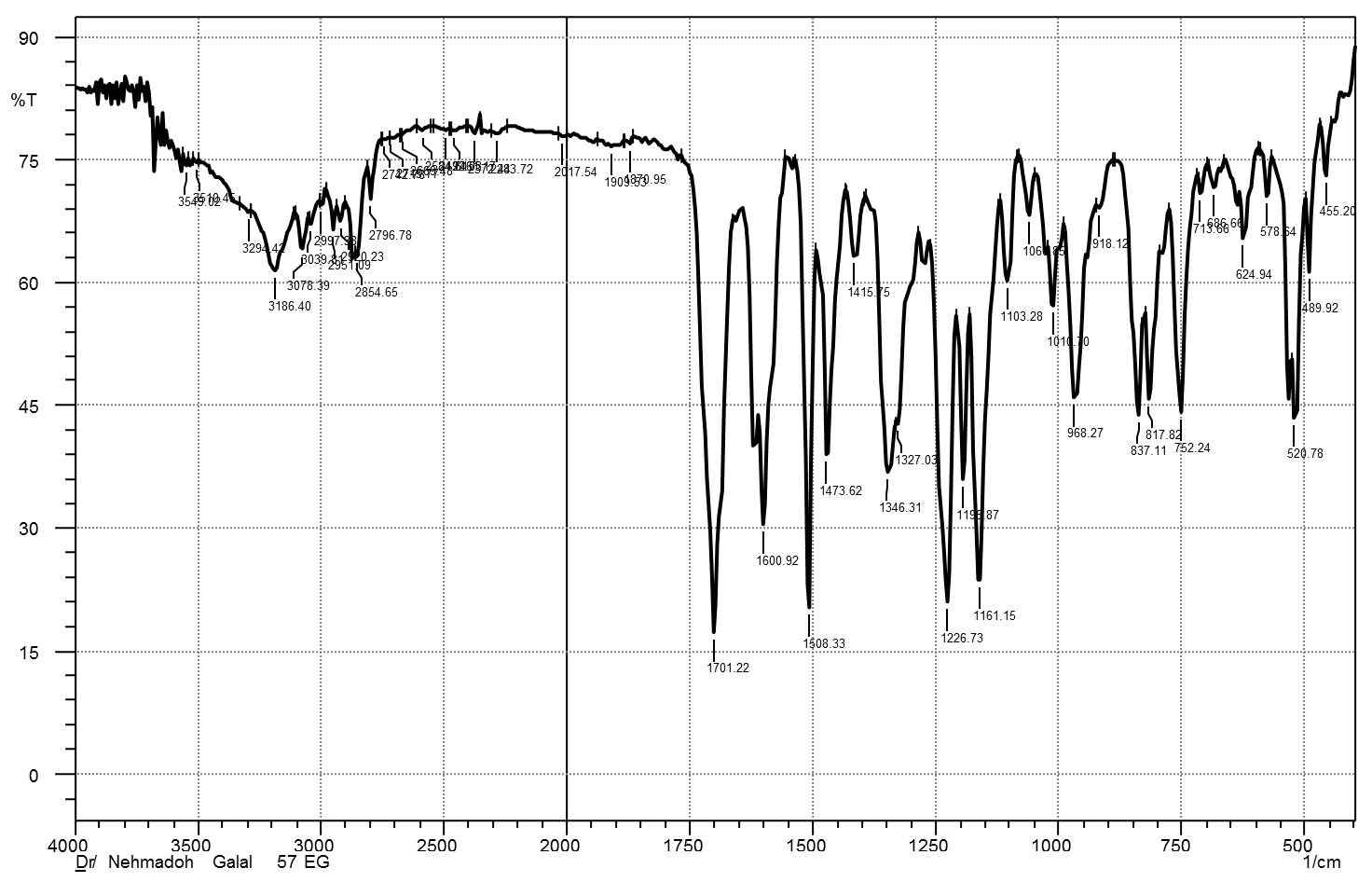


**Fig. S13.** IR spectrum of compound **6e** (KBr pellet).


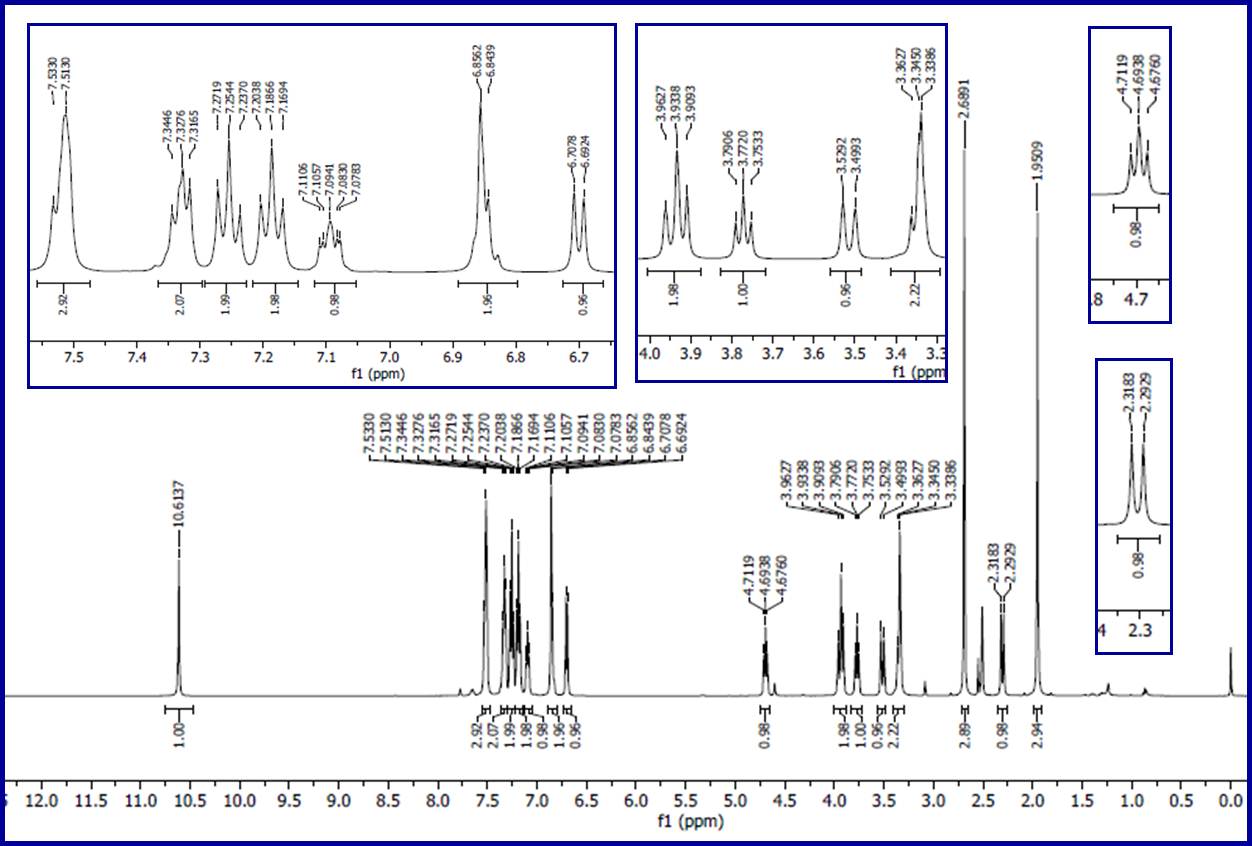


**Fig. S14.** ^1^H-NMR spectrum of compound **6e** in DMSO-*d_6_*.


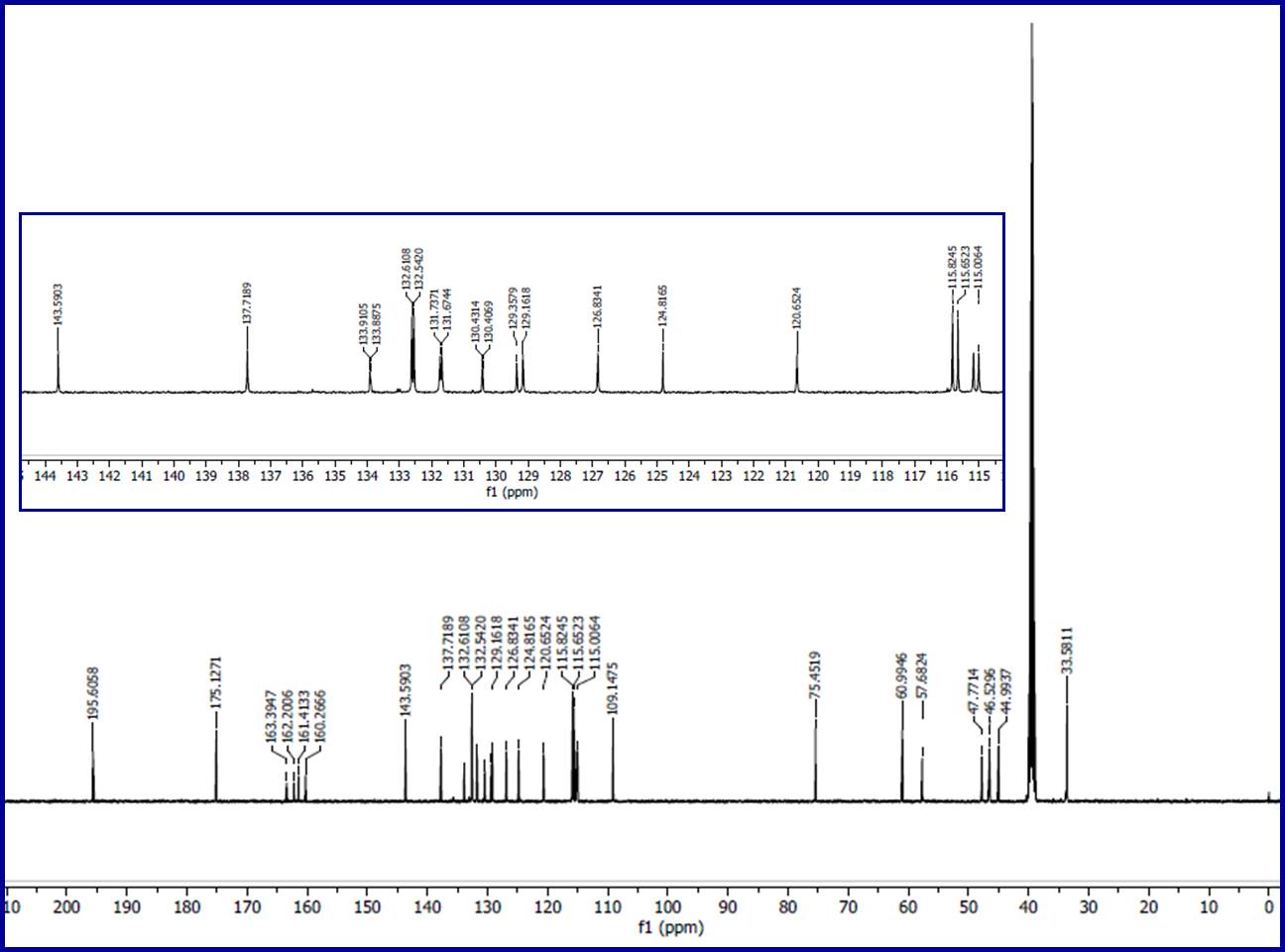


**Fig. S15.** ^13^C-NMR spectrum of compound **6e** in DMSO-*d_6_*.


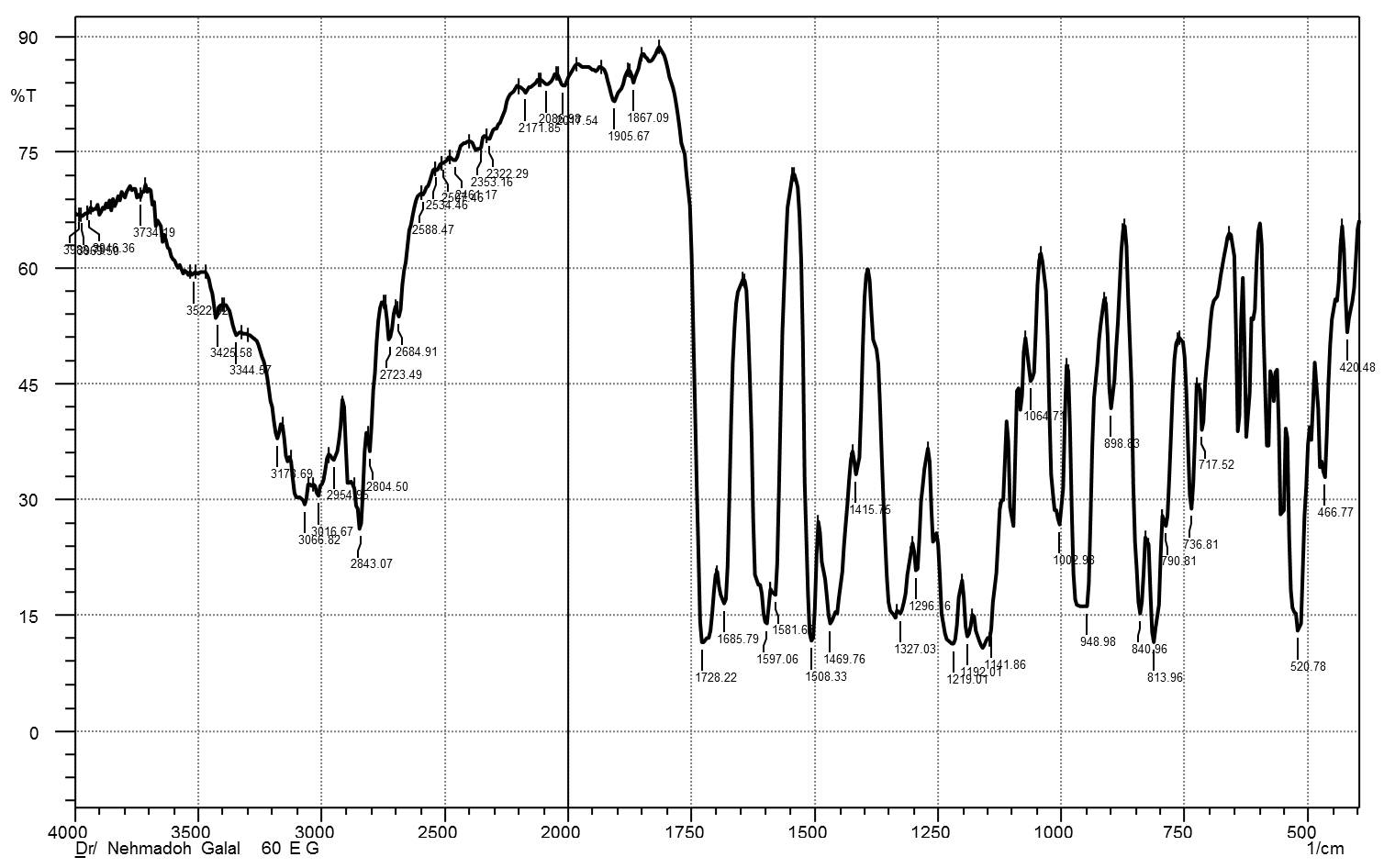


**Fig. S16.** IR spectrum of compound **6f** (KBr pellet).


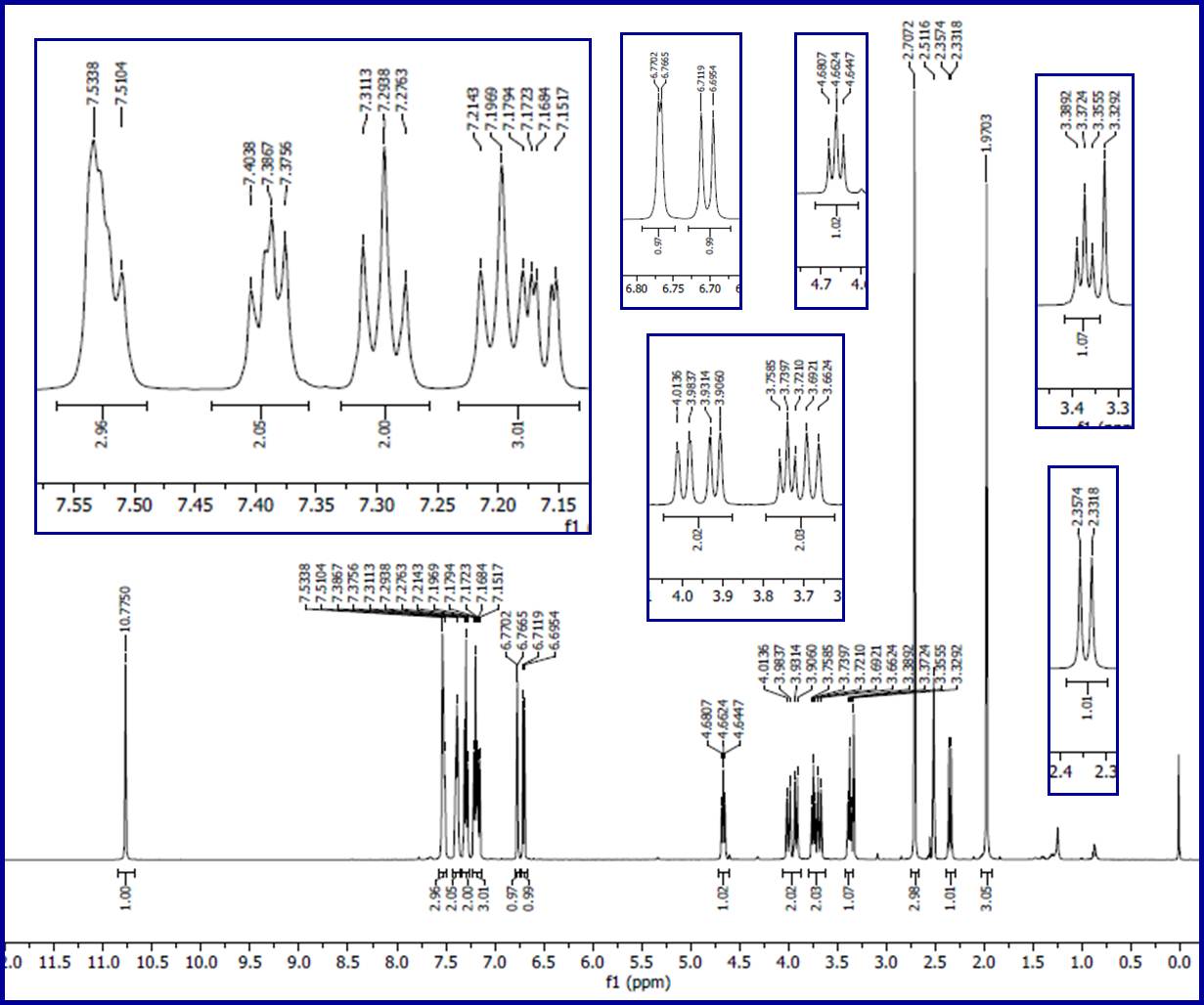


**Fig. S17.** ^1^H-NMR spectrum of compound **6f** in DMSO-*d_6_*.


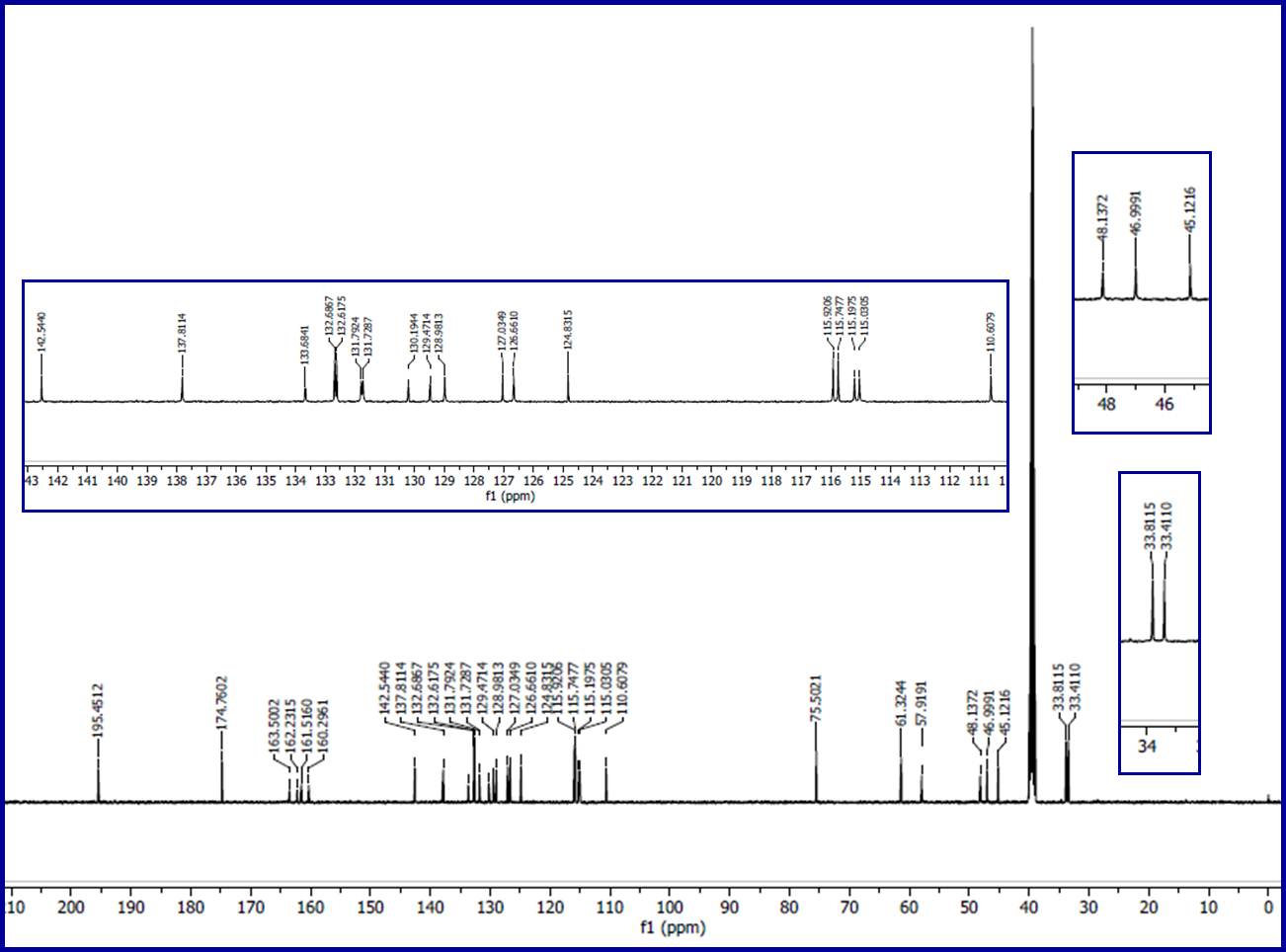


**Fig. S18.** ^13^C-NMR spectrum of compound **6f** in DMSO-*d_6_*.


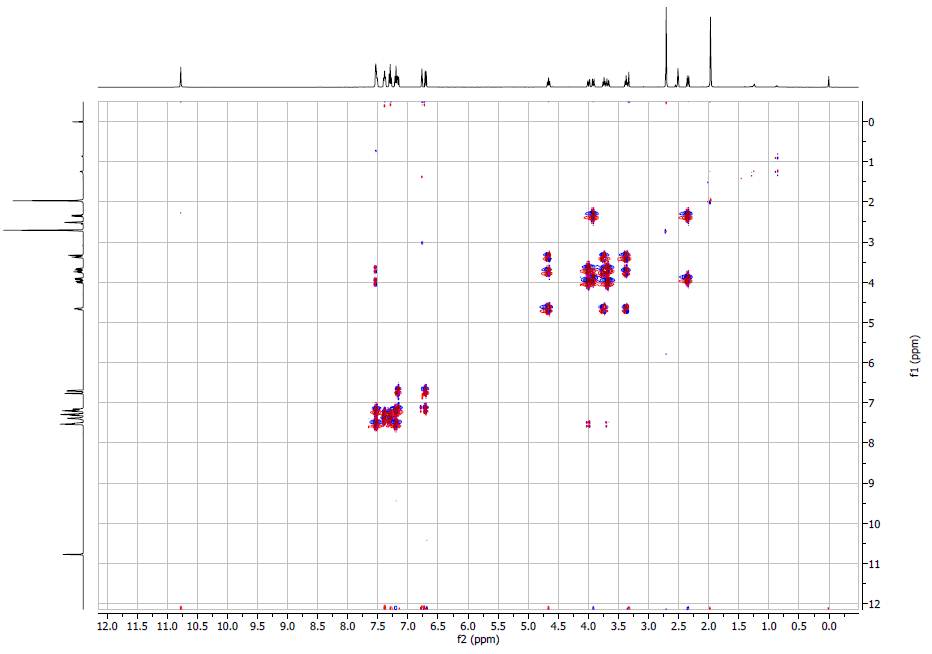


**Fig. S19A.** ^1^H, ^1^H- COSY spectrum of compound **6f** in DMSO-*d_6_*.


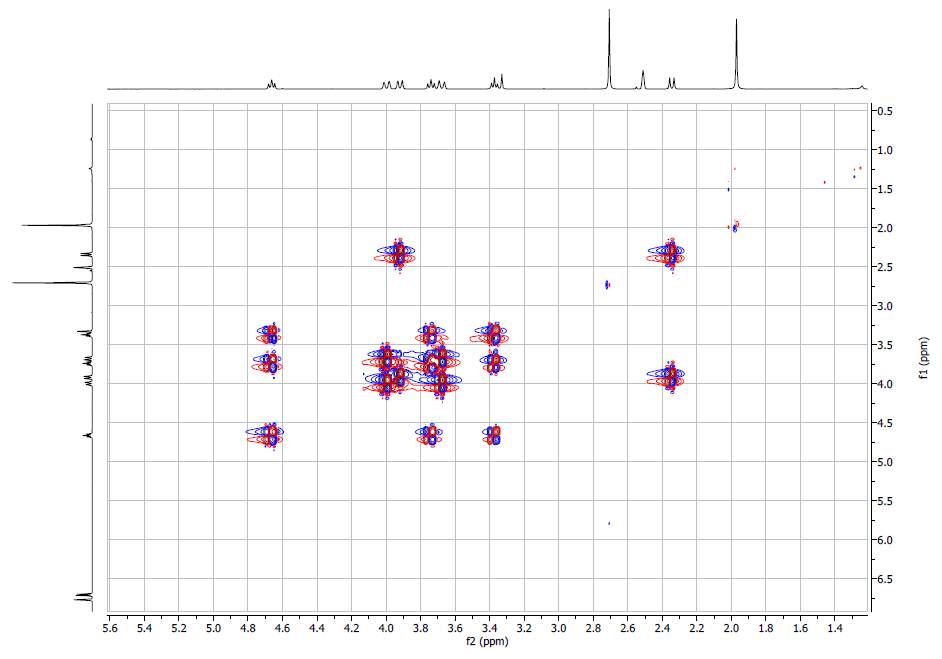


**Fig. S19B.** ^1^H, ^1^H- COSY spectrum of compound **6f** in DMSO-*d_6_* (expansion of δ = 1.4-5.6).


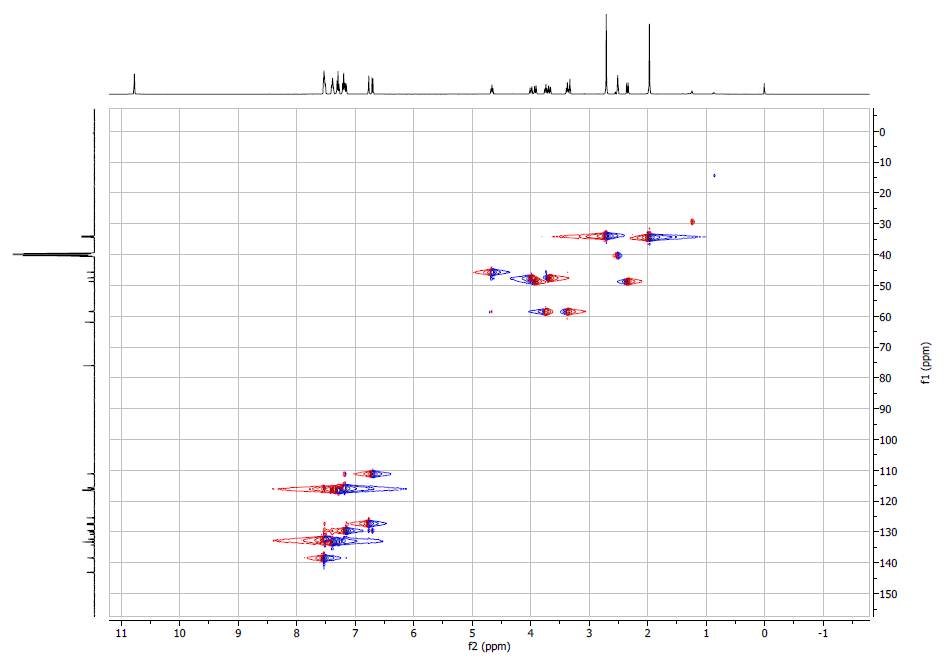


**Fig. S20A.** HSQC spectrum of compound **6f** in DMSO-*d_6_*.


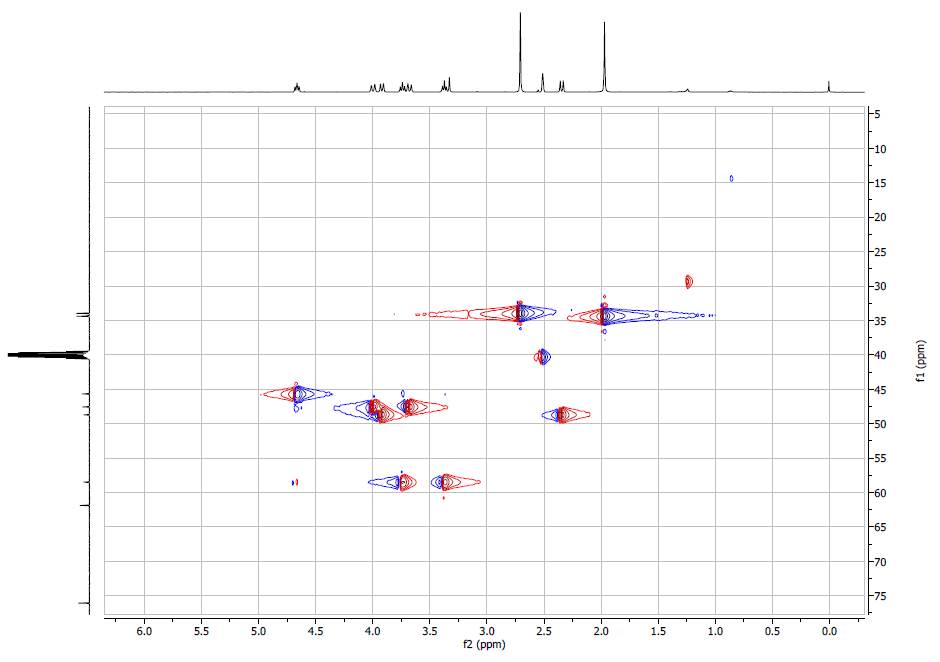


**Fig. S20B.** HSQC spectrum of compound **6f** in DMSO-*d_6_*.(expansion of δ_H_ = 0-6.0; δ_C_ = 5-75).


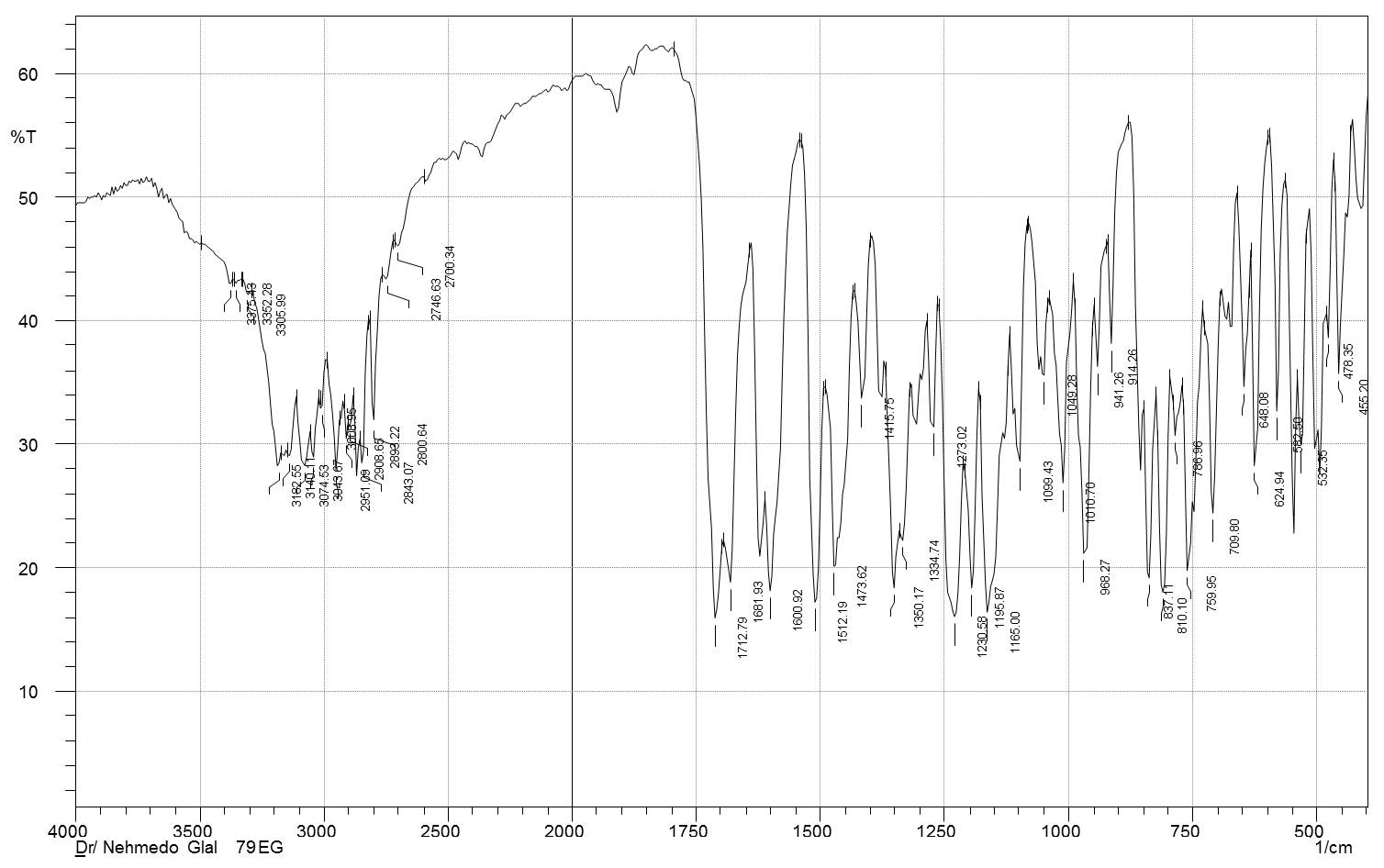


**Fig. S21.** IR spectrum of compound **6g** (KBr pellet).


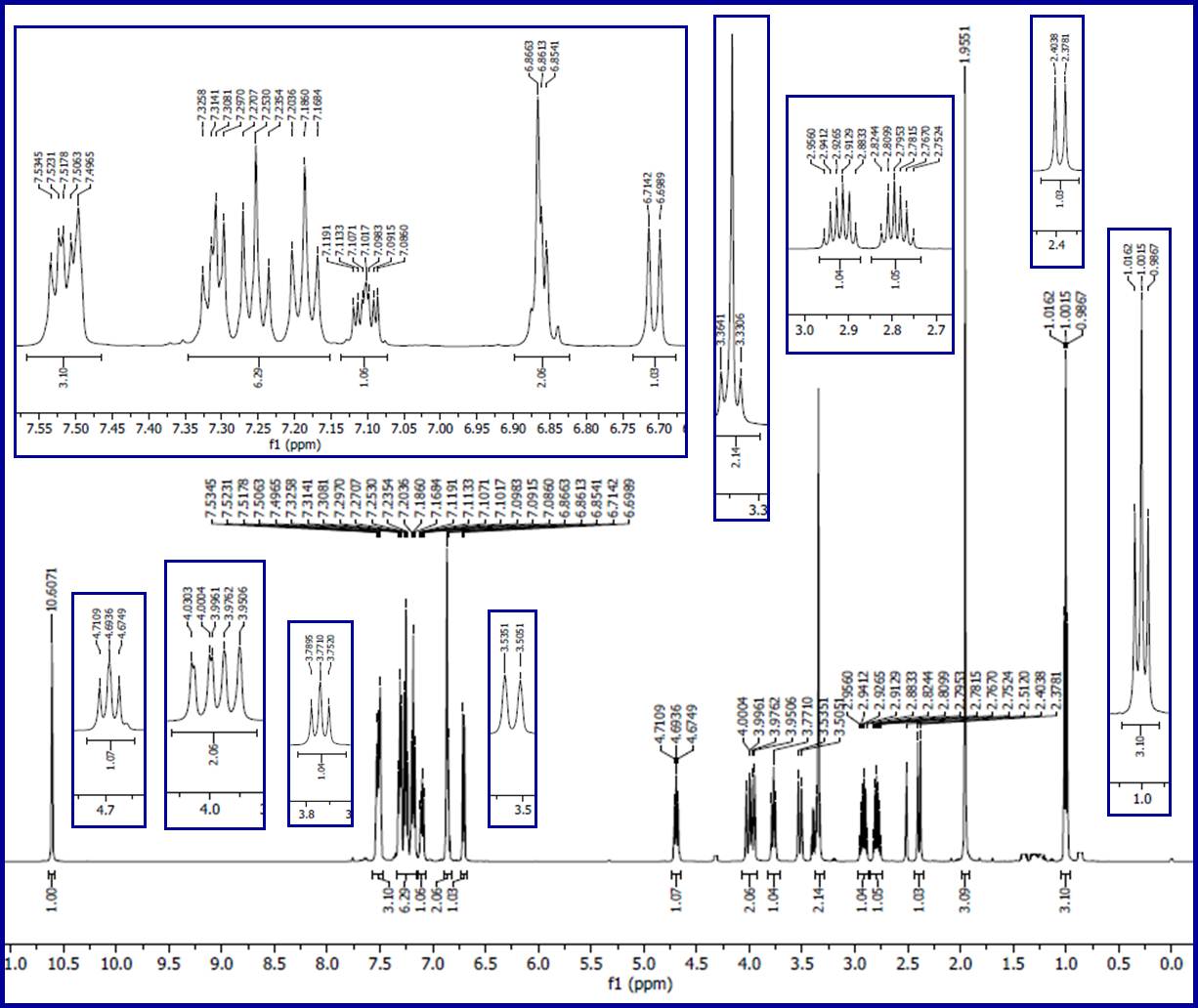


**Fig. S22.** ^1^H-NMR spectrum of compound **6g** in DMSO-*d_6_*.


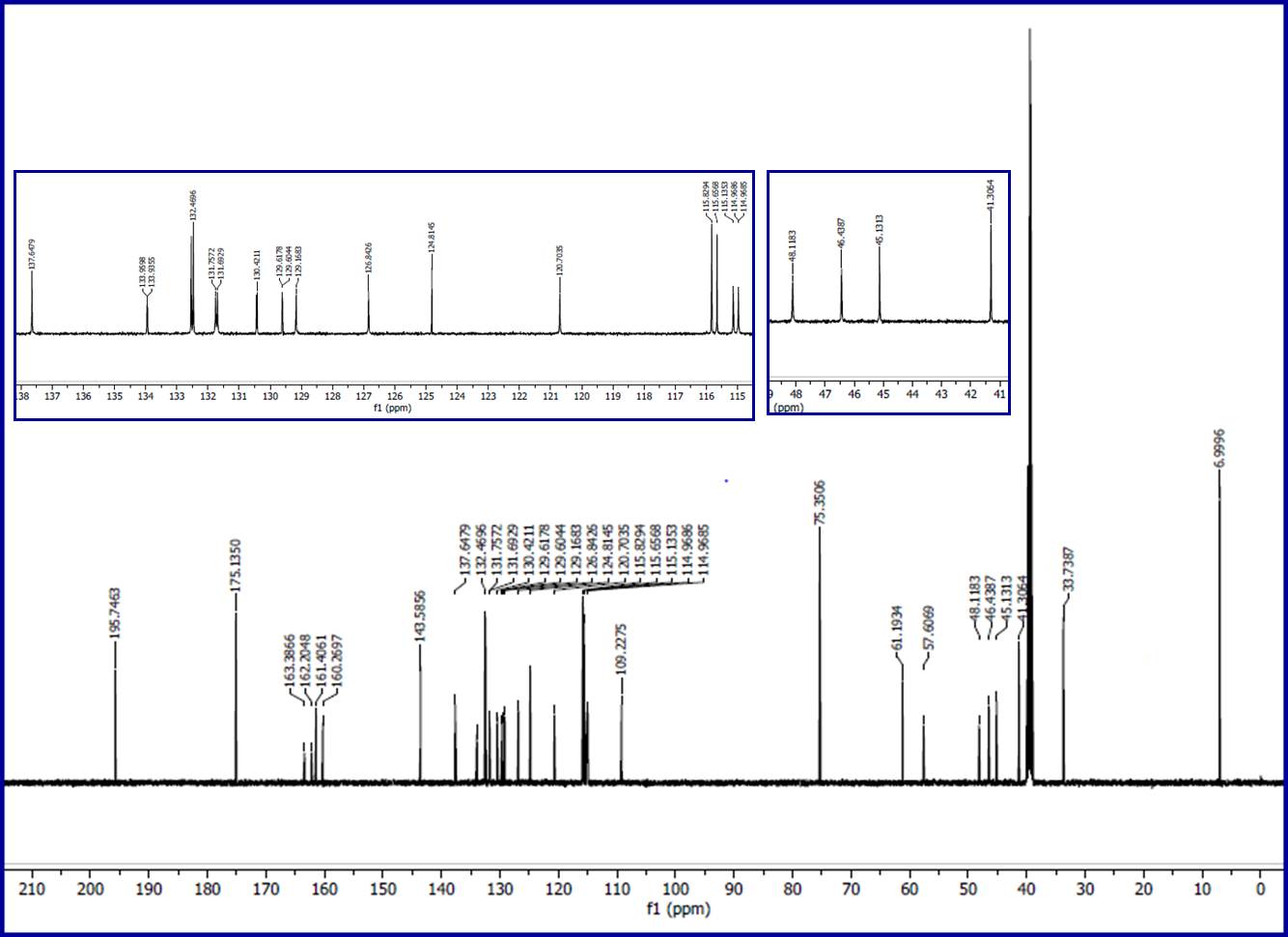


**Fig. S23.** ^13^C-NMR spectrum of compound **6g** in DMSO-*d_6_*.


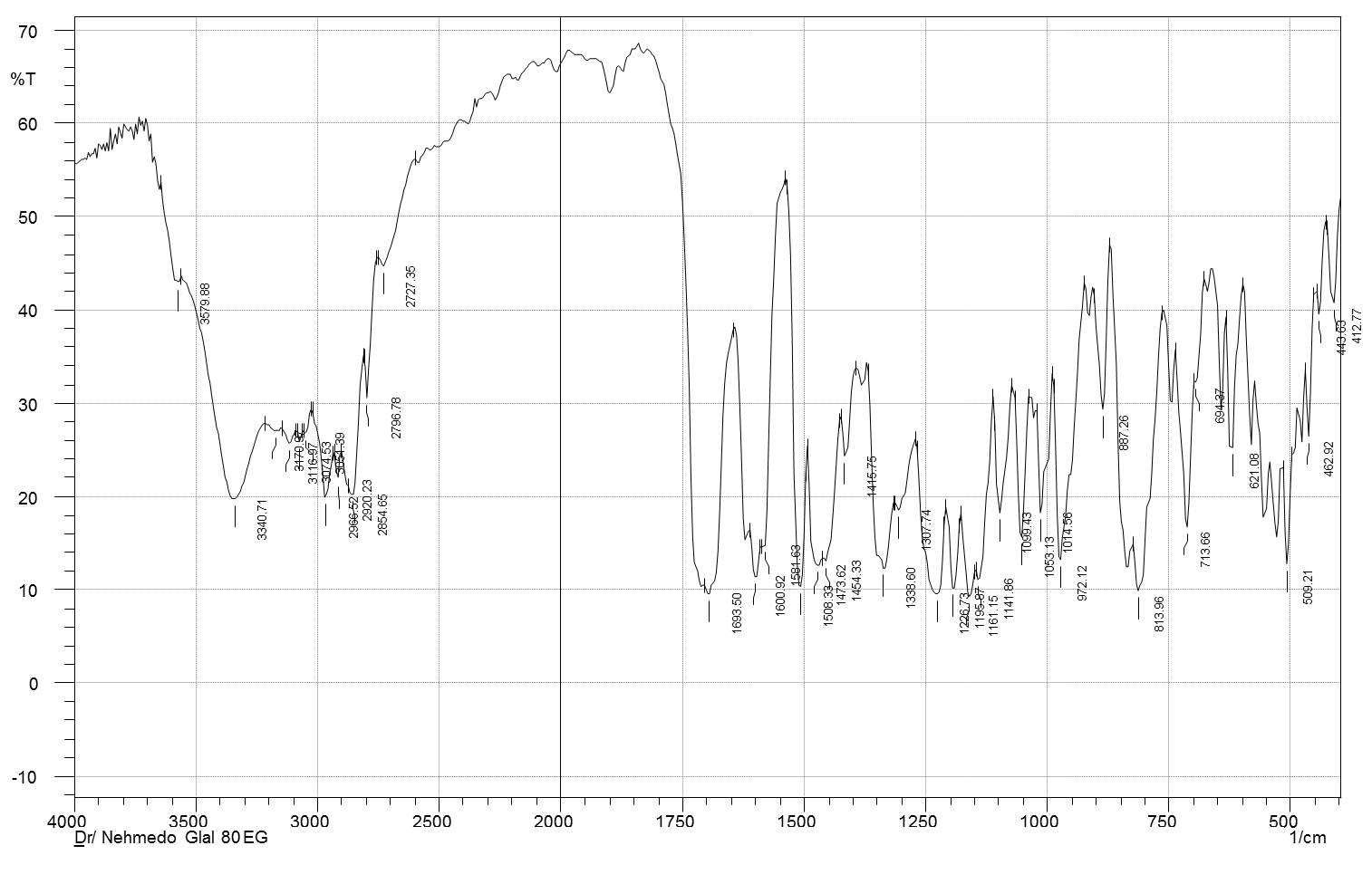
**Fig. S24.** IR spectrum of compound **6h** (KBr pellet).


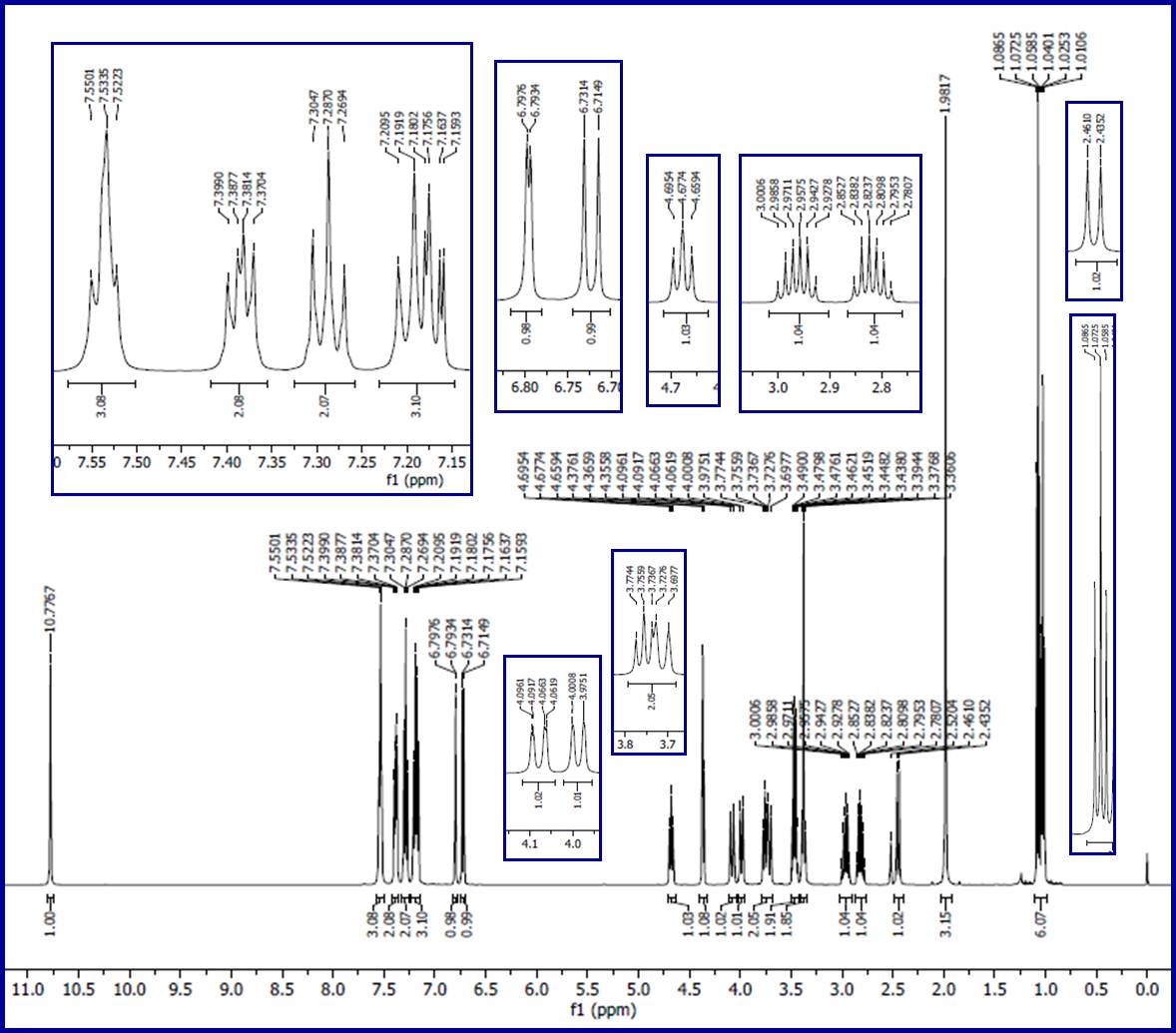


**Fig. S25.** ^1^H-NMR spectrum of compound **6h** in DMSO-*d_6_* (the signals at δ = 1.02 and 3.46, 4.37 are due to the solvent of crystallization, ethanol, Org. Process Res. Dev. 2016, 20, 661−667).


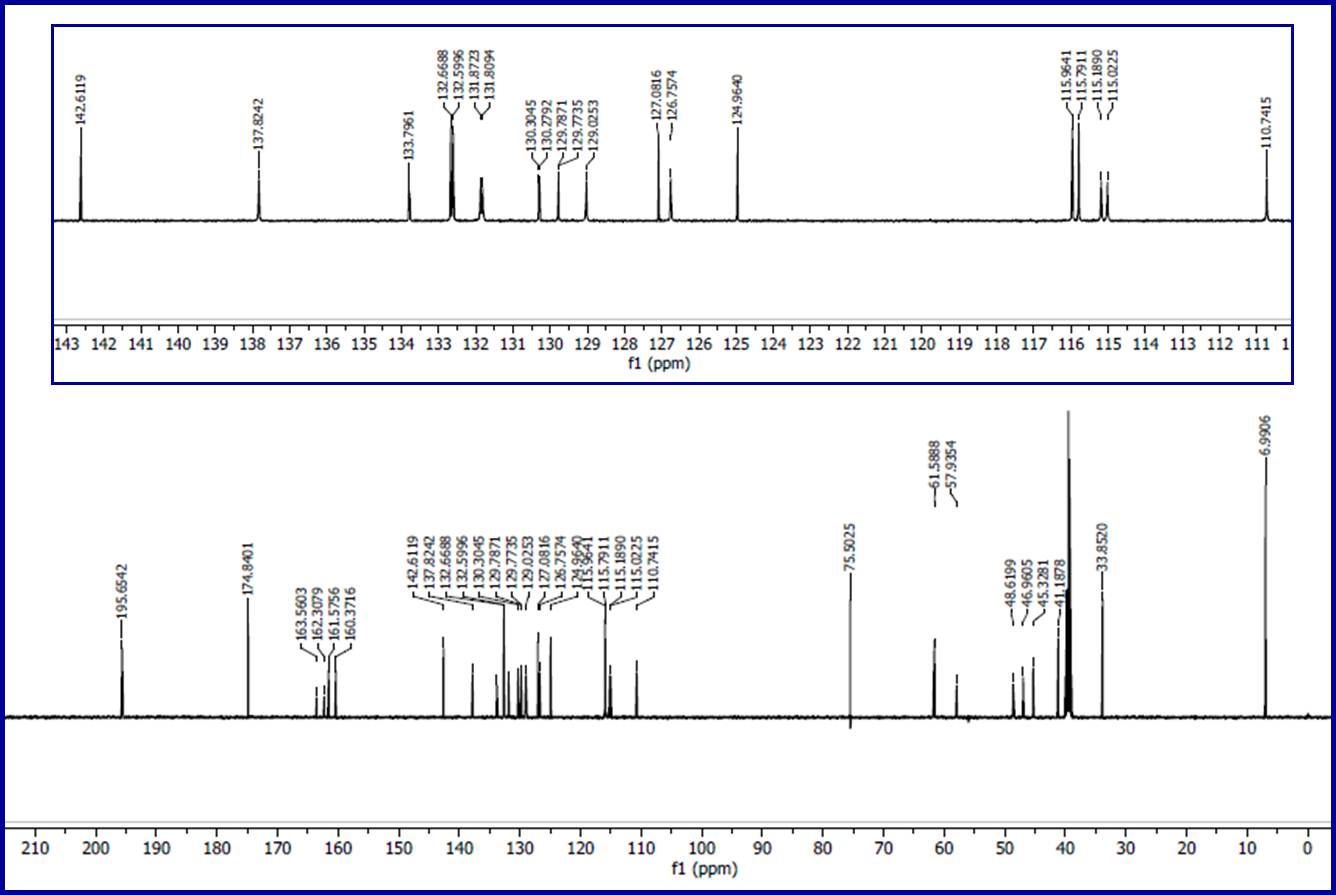


**Fig. S26.** ^13^C-NMR spectrum of compound **6h** in DMSO-*d_6_*.

**
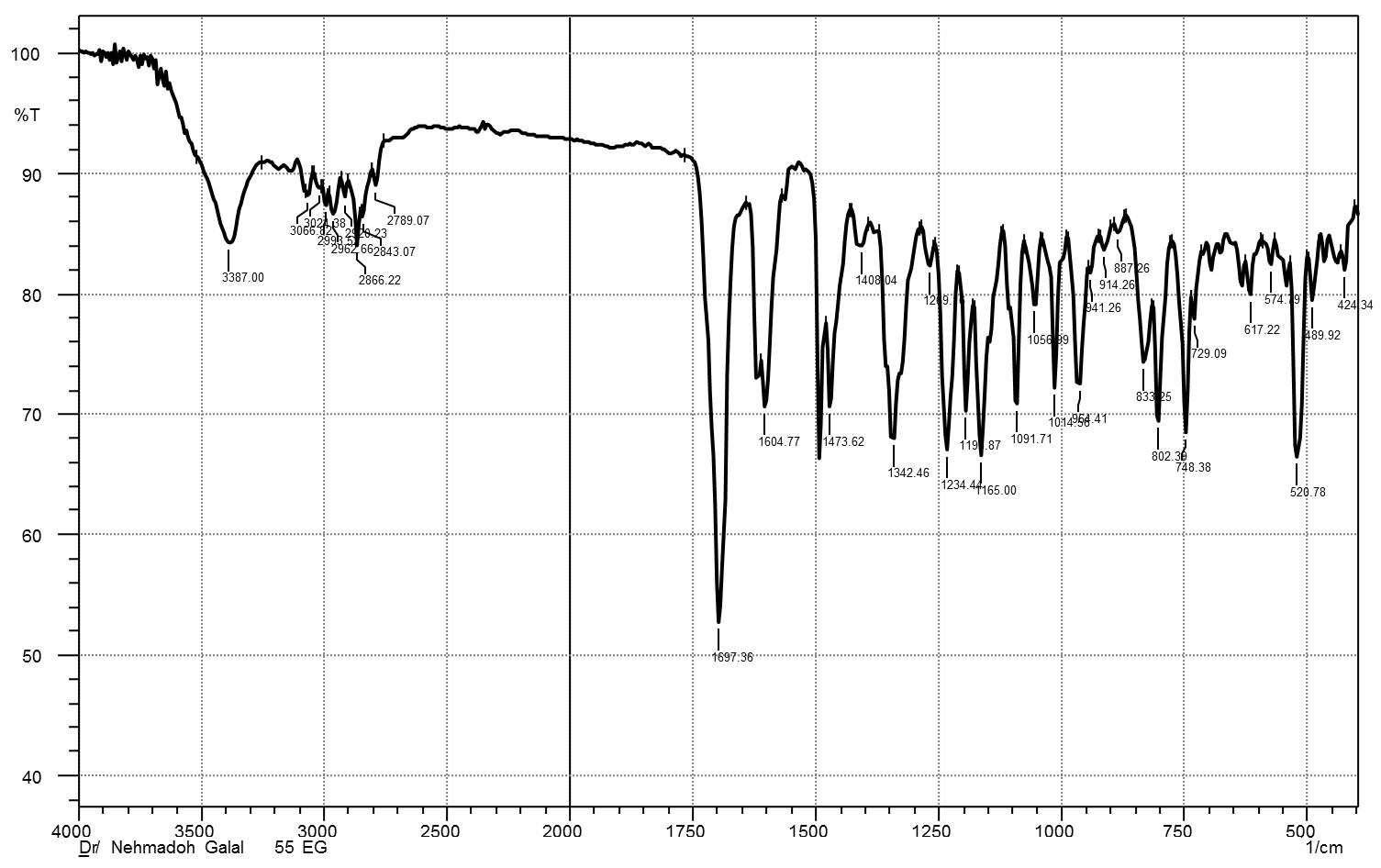
**

**Fig. S27.** IR spectrum of compound **6i** (KBr pellet).


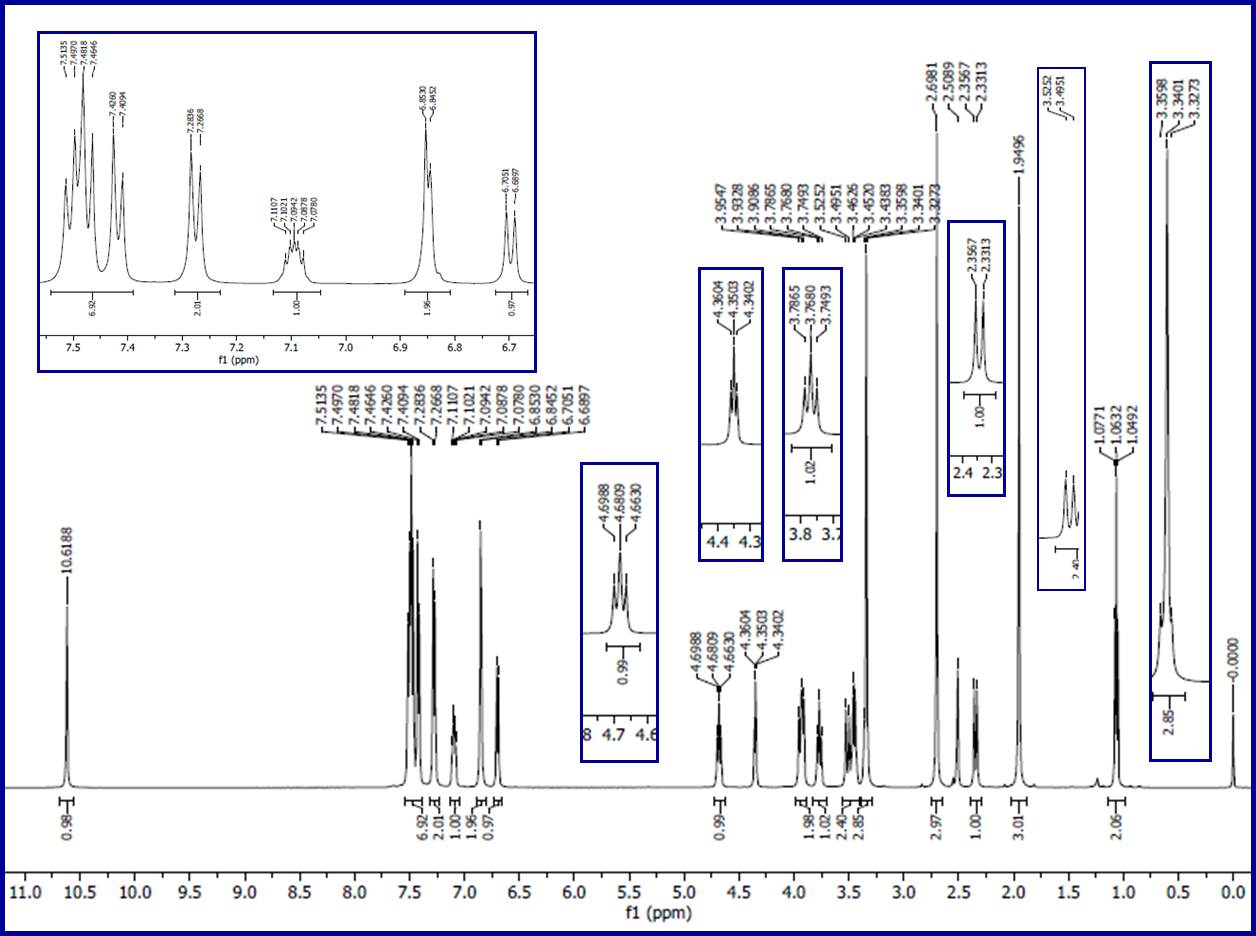


**Fig. S28.** ^1^H-NMR spectrum of compound **6i** in DMSO-*d_6_* (the signals at δ = 1.06 and 3.45, 4.35 are due to the solvent of crystallization, ethanol, Org. Process Res. Dev. 2016, 20, 661−667).


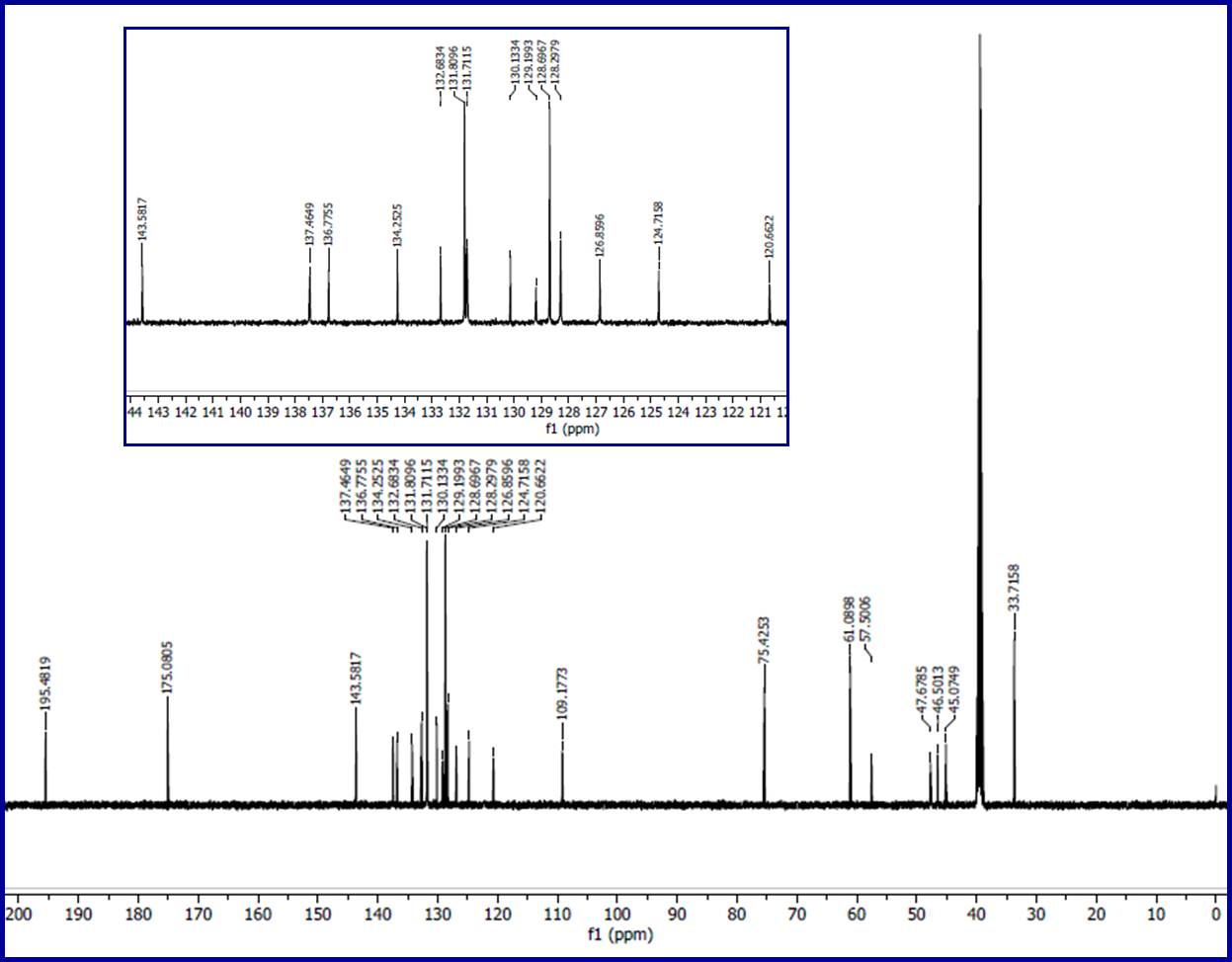


**Fig. S29.** ^13^C-NMR spectrum of compound **6i** in DMSO-*d_6_*.


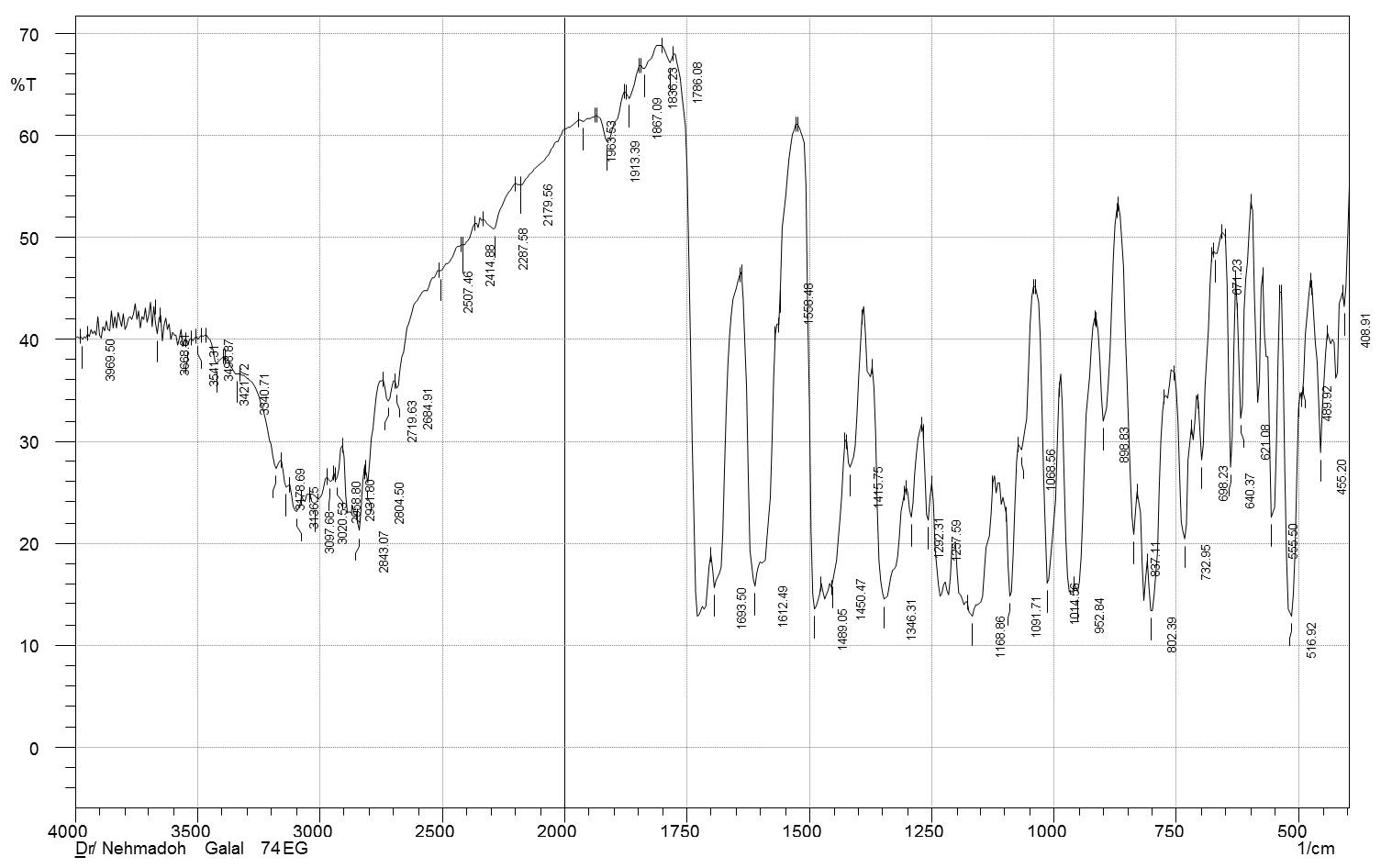


**Fig. S30.** IR spectrum of compound **6j** (KBr pellet).


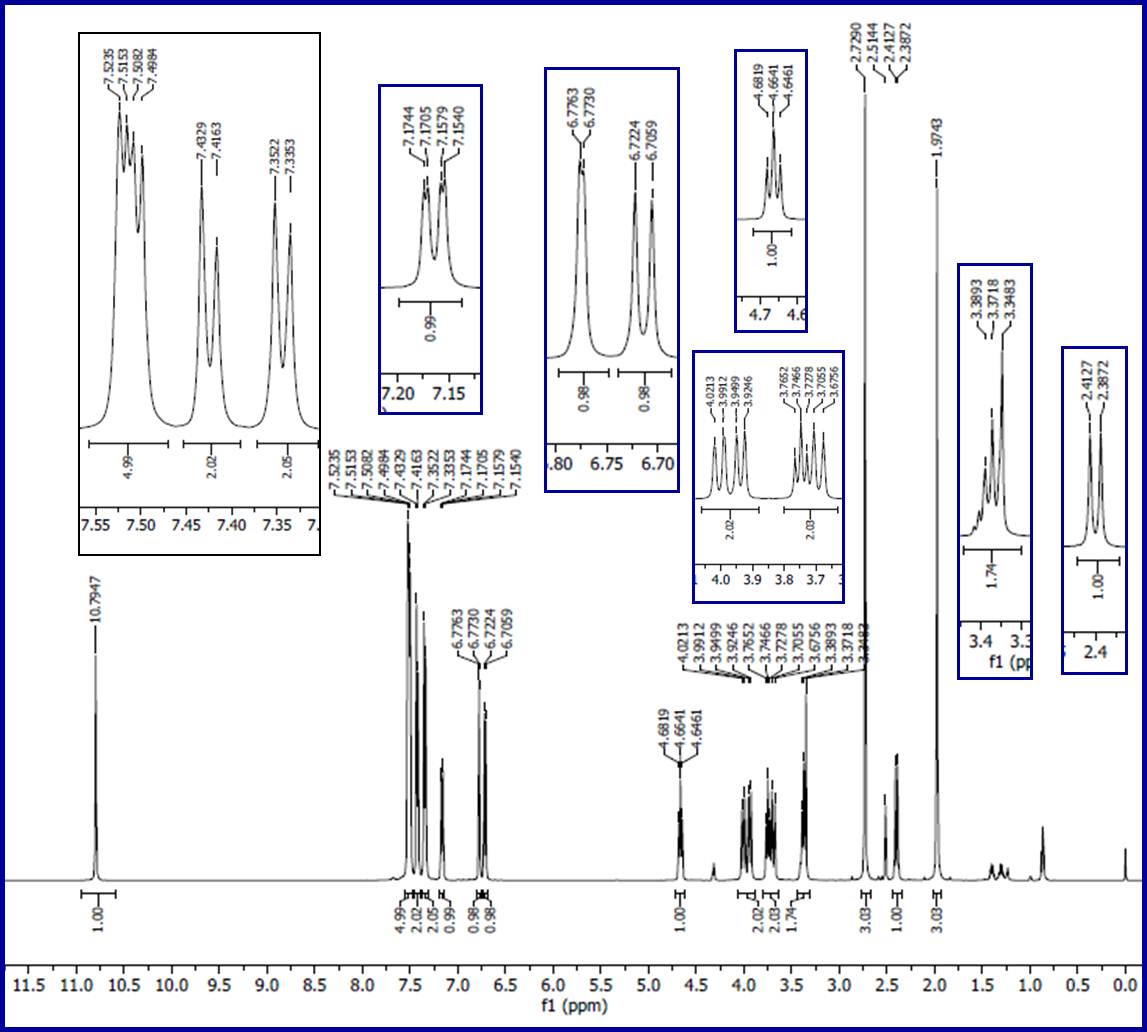


**Fig. S31.** ^1^H-NMR spectrum of compound **6j** in DMSO-*d_6_*.


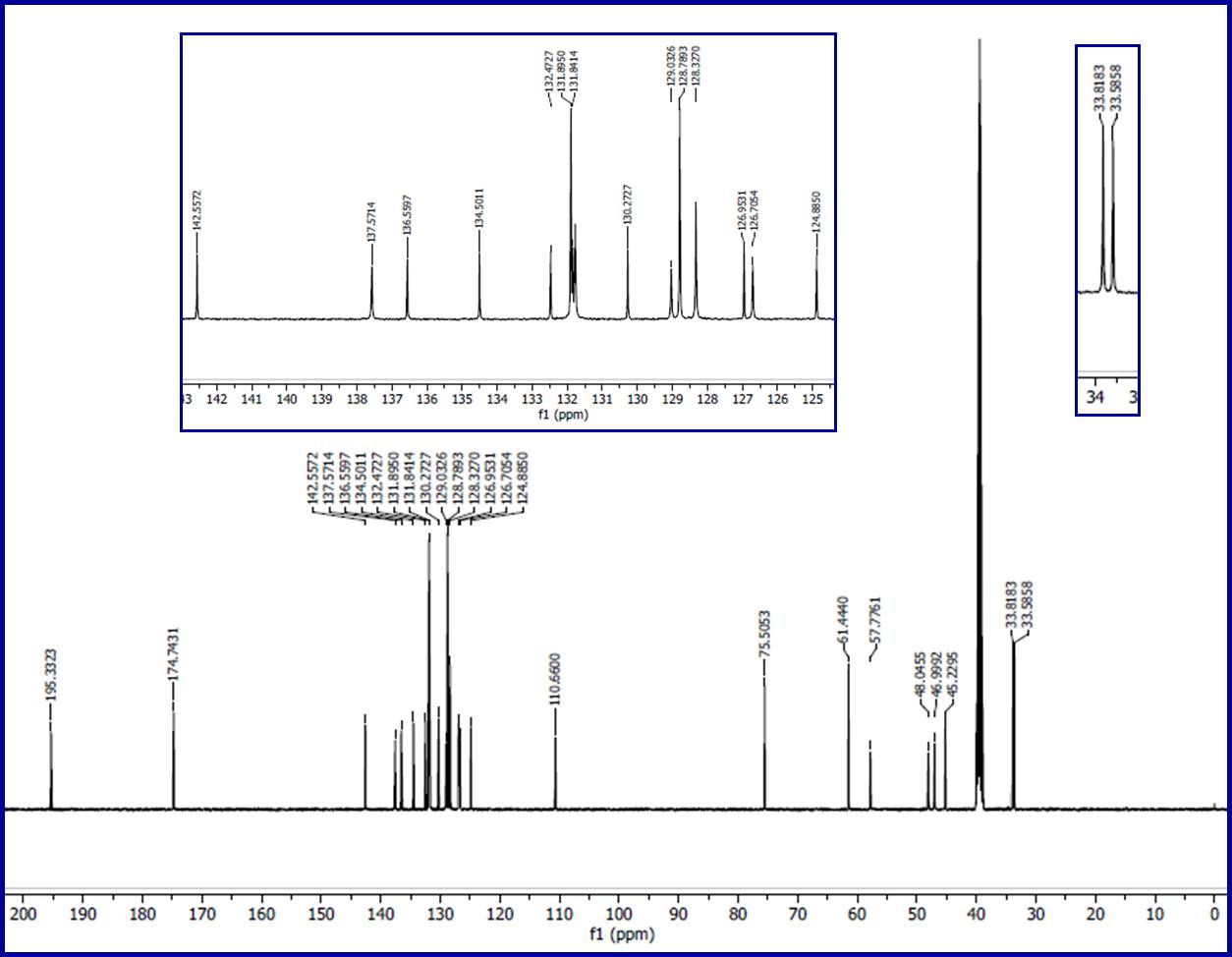


**Fig. S32.** ^13^C-NMR spectrum of compound **6j** in DMSO-*d_6_*.


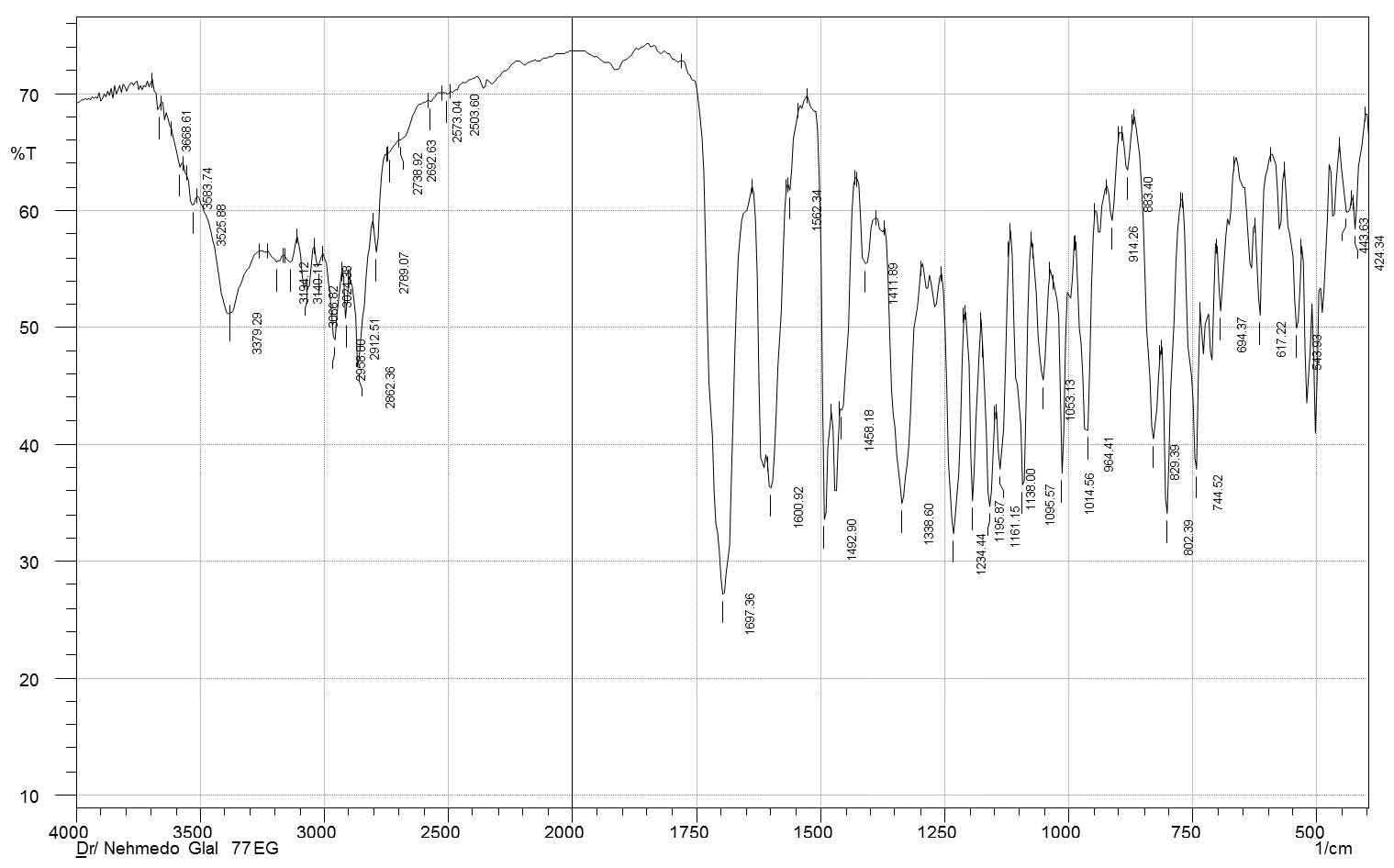


**Fig. S33.** IR spectrum of compound **6k** (KBr pellet).


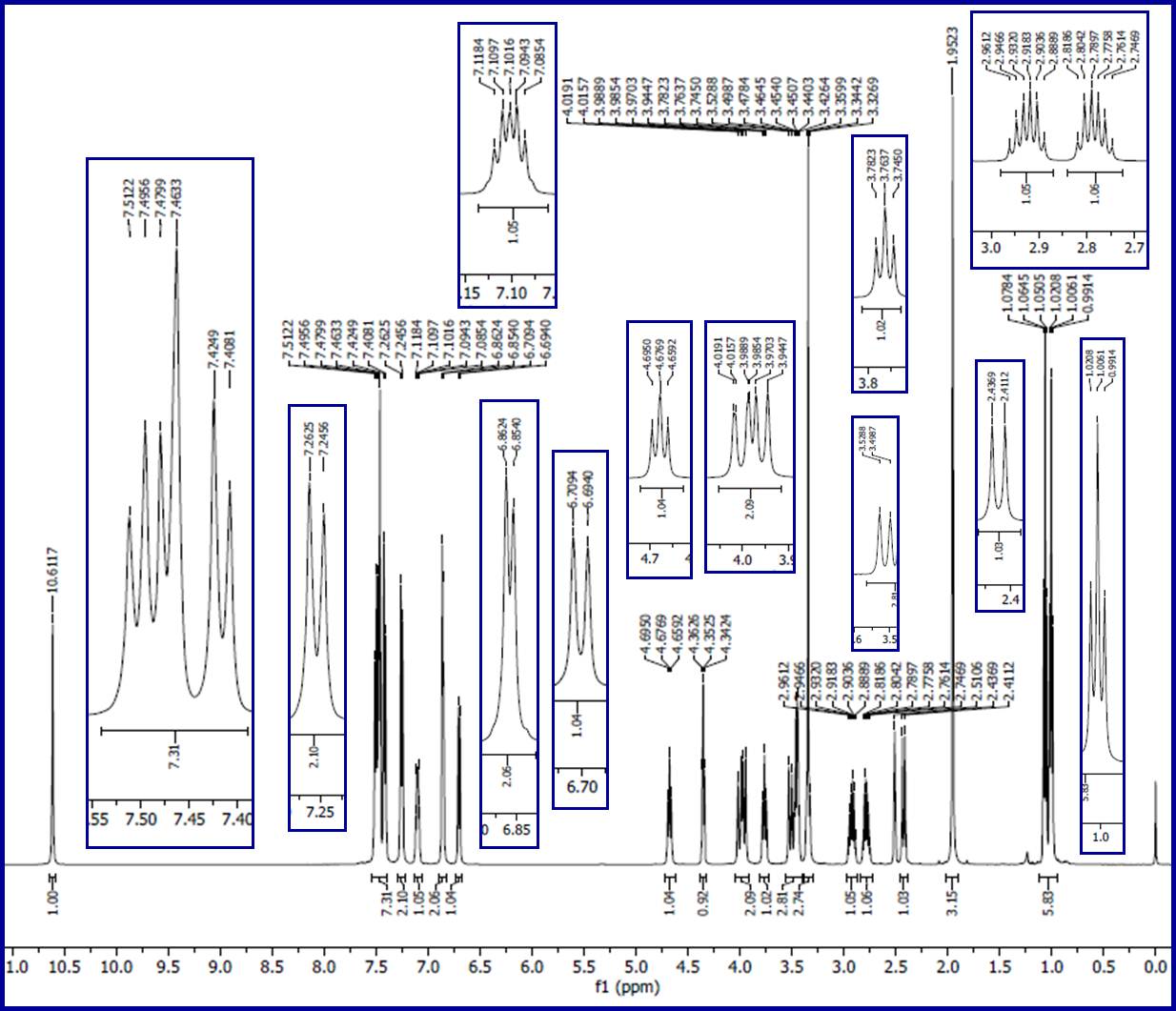


**Fig. S34.** ^1^H-NMR spectrum of compound **6k** in DMSO-*d_6_* (the signals at δ = 1.06 and 3.45, 4.35 are due to the solvent of crystallization, ethanol, Org. Process Res. Dev. 2016, 20, 661−667).


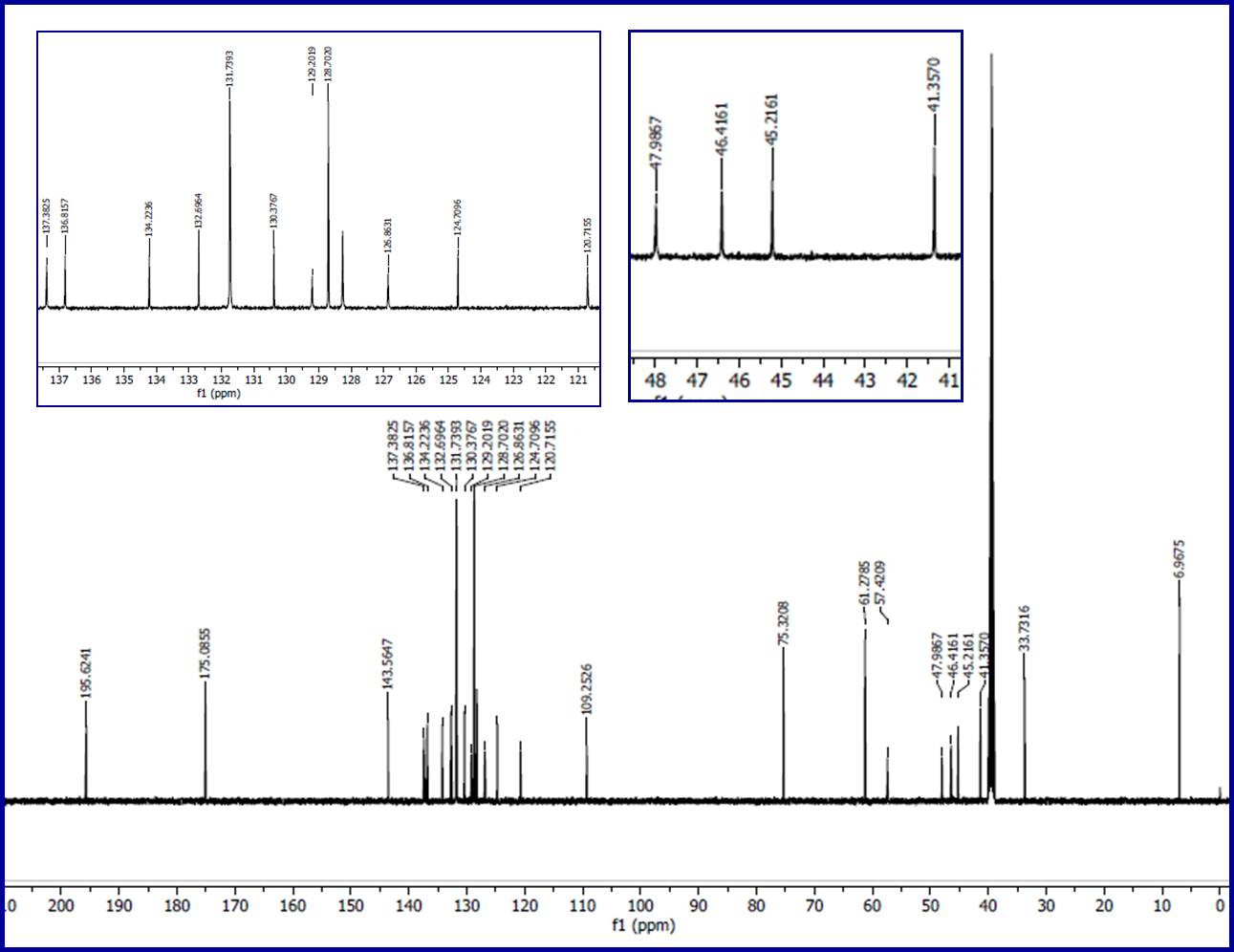


**Fig. S35.** ^13^C-NMR spectrum of compound **6k** in DMSO-*d_6_*.


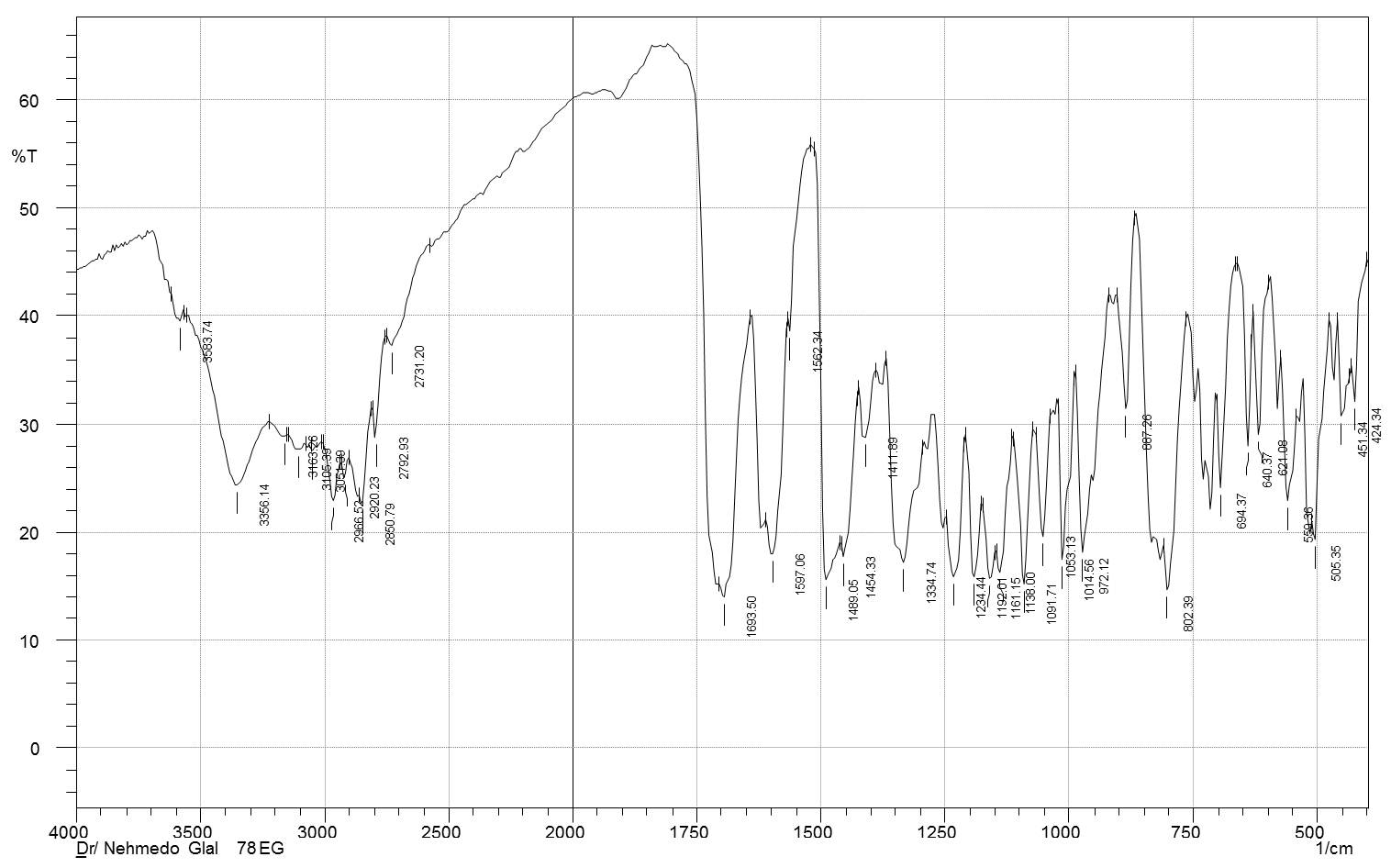


**Fig. S36.** IR spectrum of compound **6l** (KBr pellet).


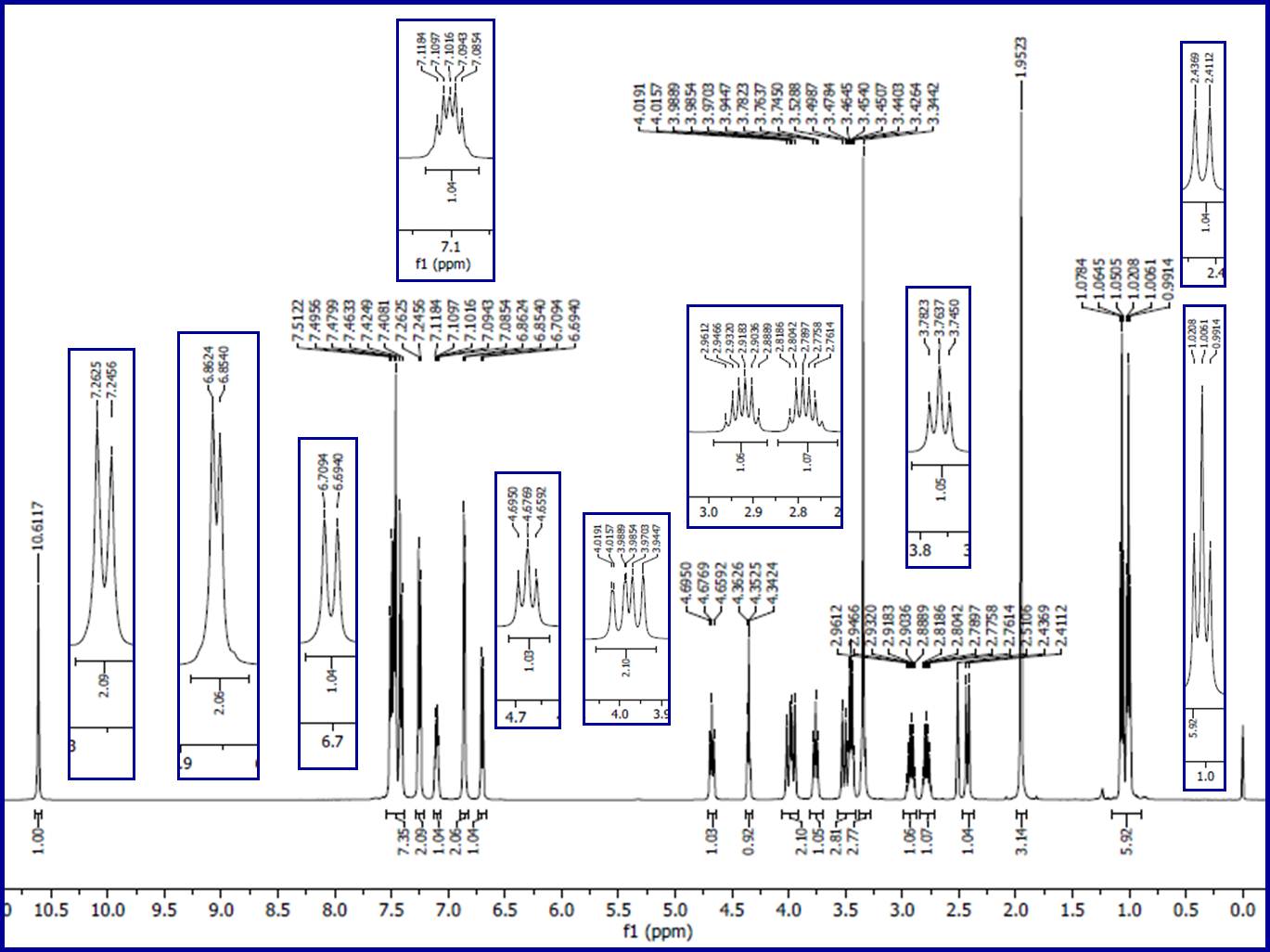


**Fig. S37.** ^1^H-NMR spectrum of compound **6l** in DMSO-*d_6_* (the signals at δ = 1.06 and 3.45, 4.35 are due to the solvent of crystallization, ethanol, Org. Process Res. Dev. 2016, 20, 661−667).


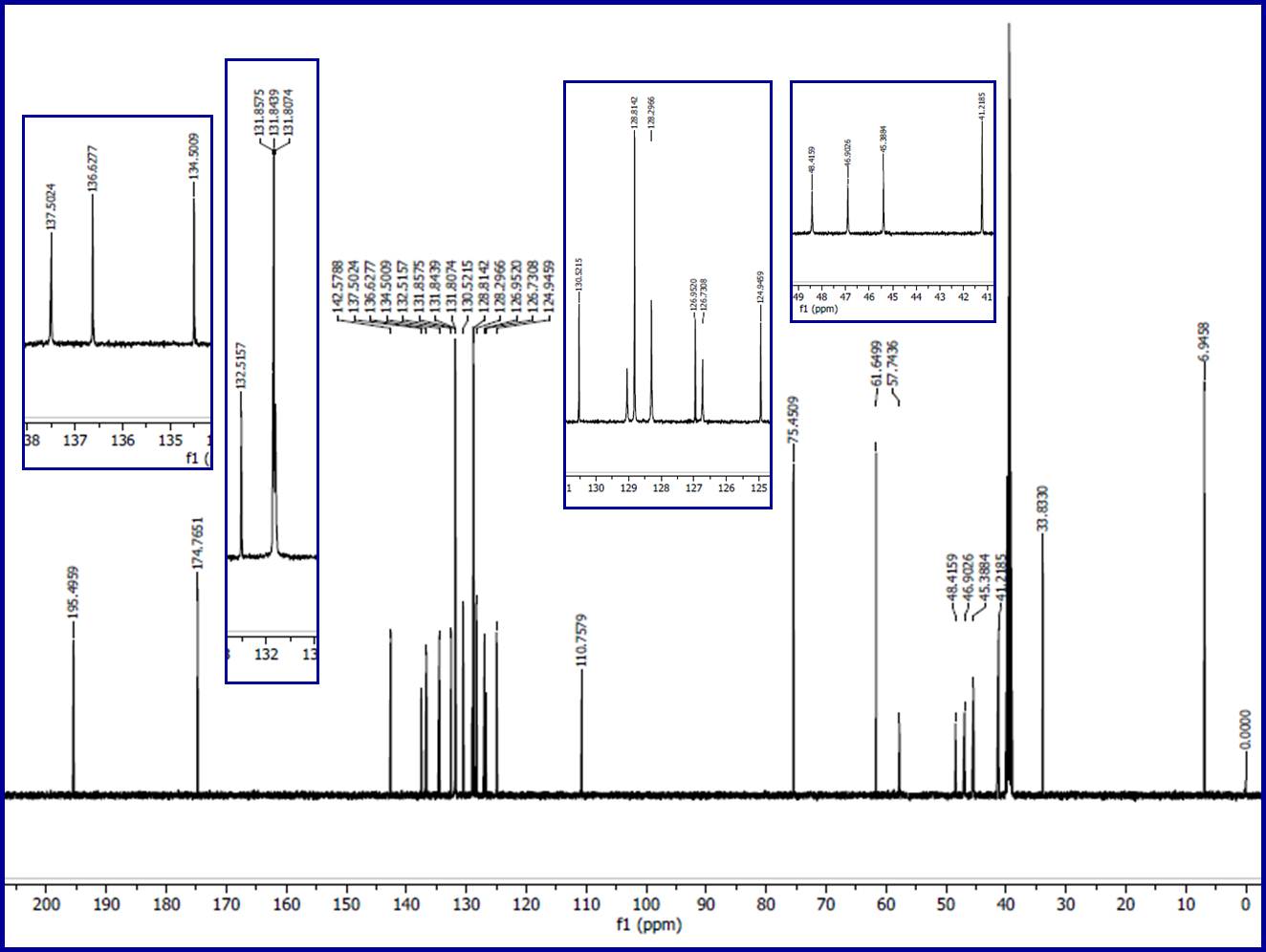


**Fig. S38.** ^13^C-NMR spectrum of compound **6l** in DMSO-*d_6_*.


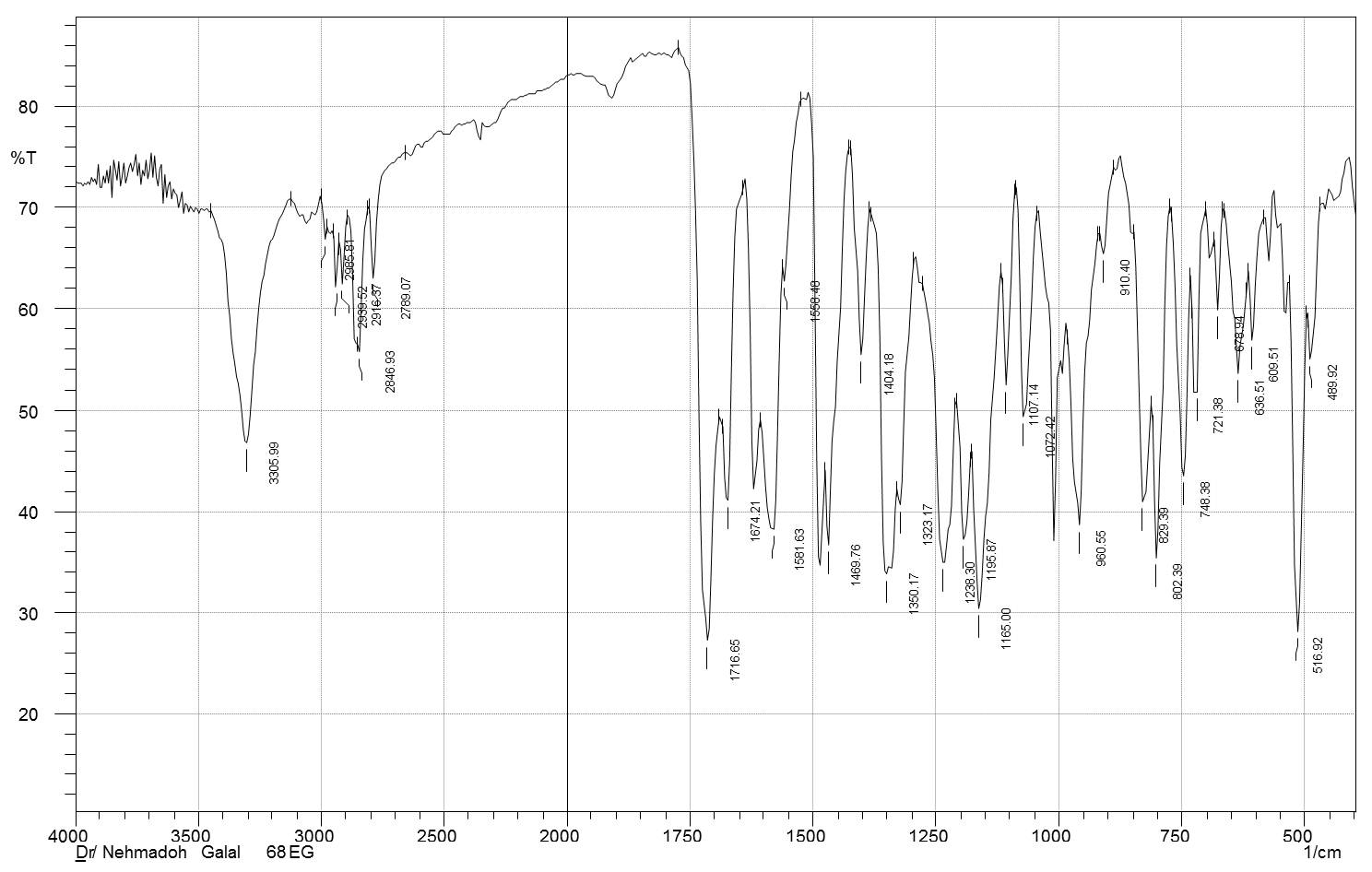


**Fig. S39.** IR spectrum of compound **6m** (KBr pellet).


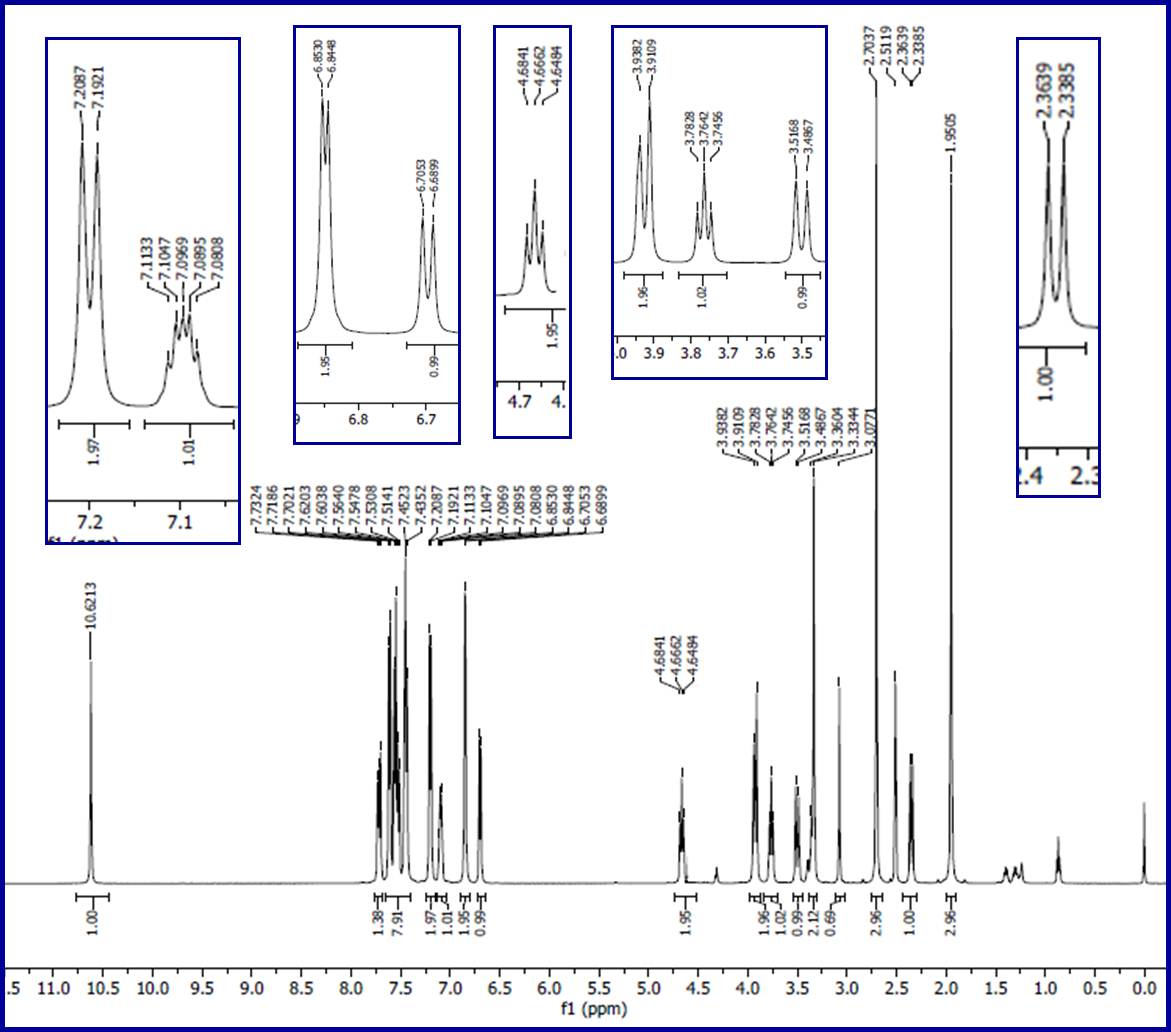


**Fig. S40.** ^1^H-NMR spectrum of compound **6m** in DMSO-*d_6_*.


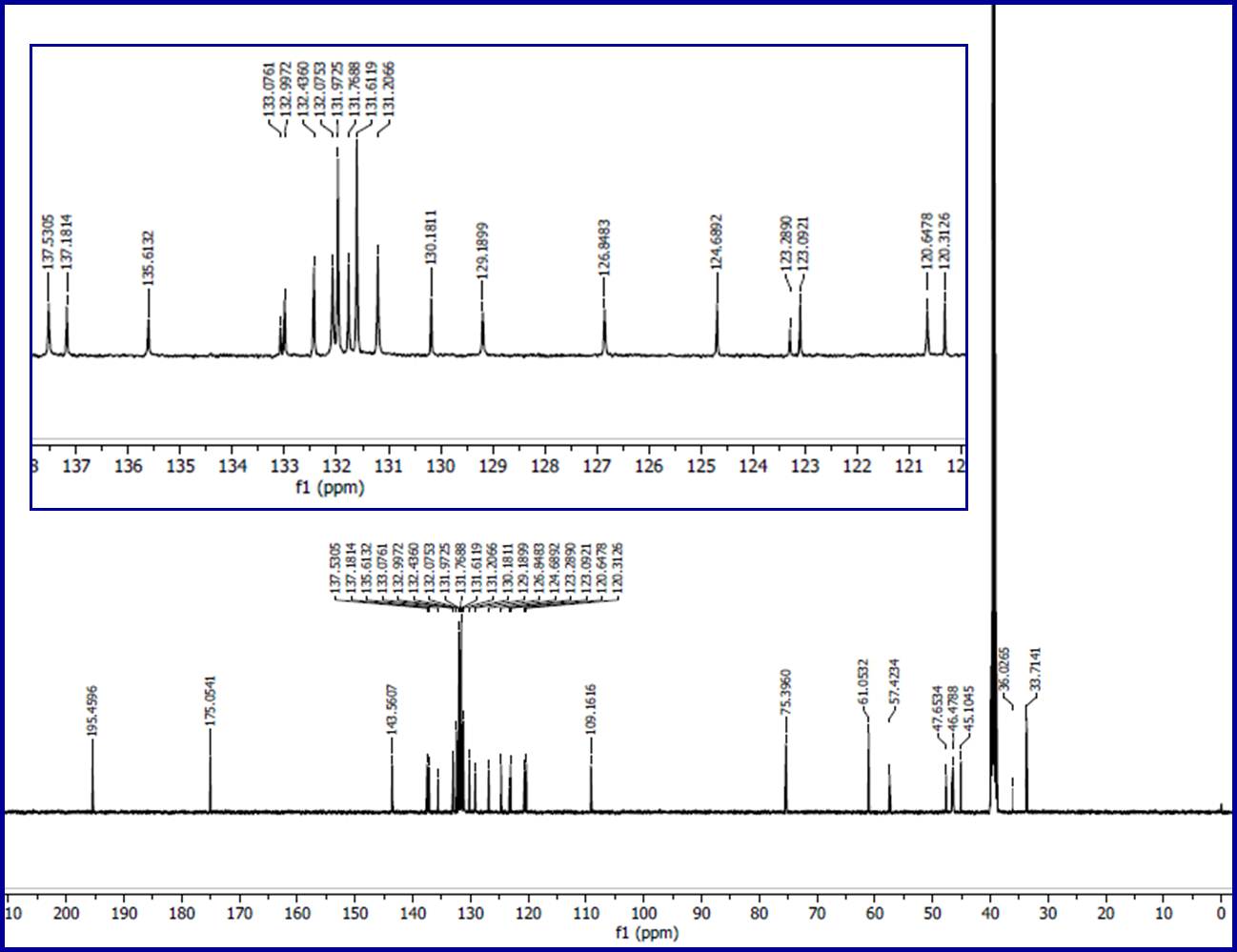


**Fig. S41.** ^13^C-NMR spectrum of compound **6m** in DMSO-*d_6_*.


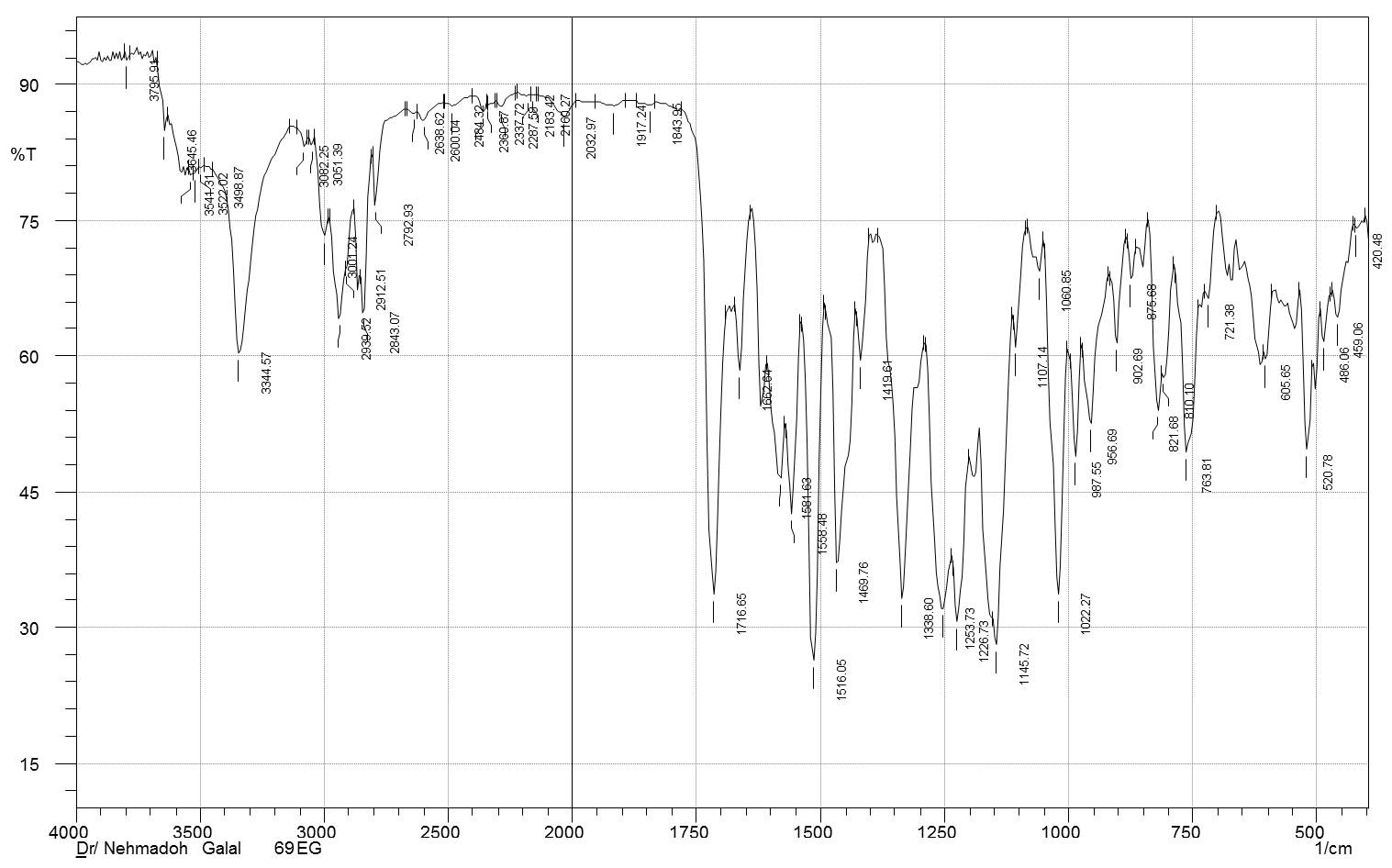


**Fig. S42.** IR spectrum of compound **6n** (KBr pellet).


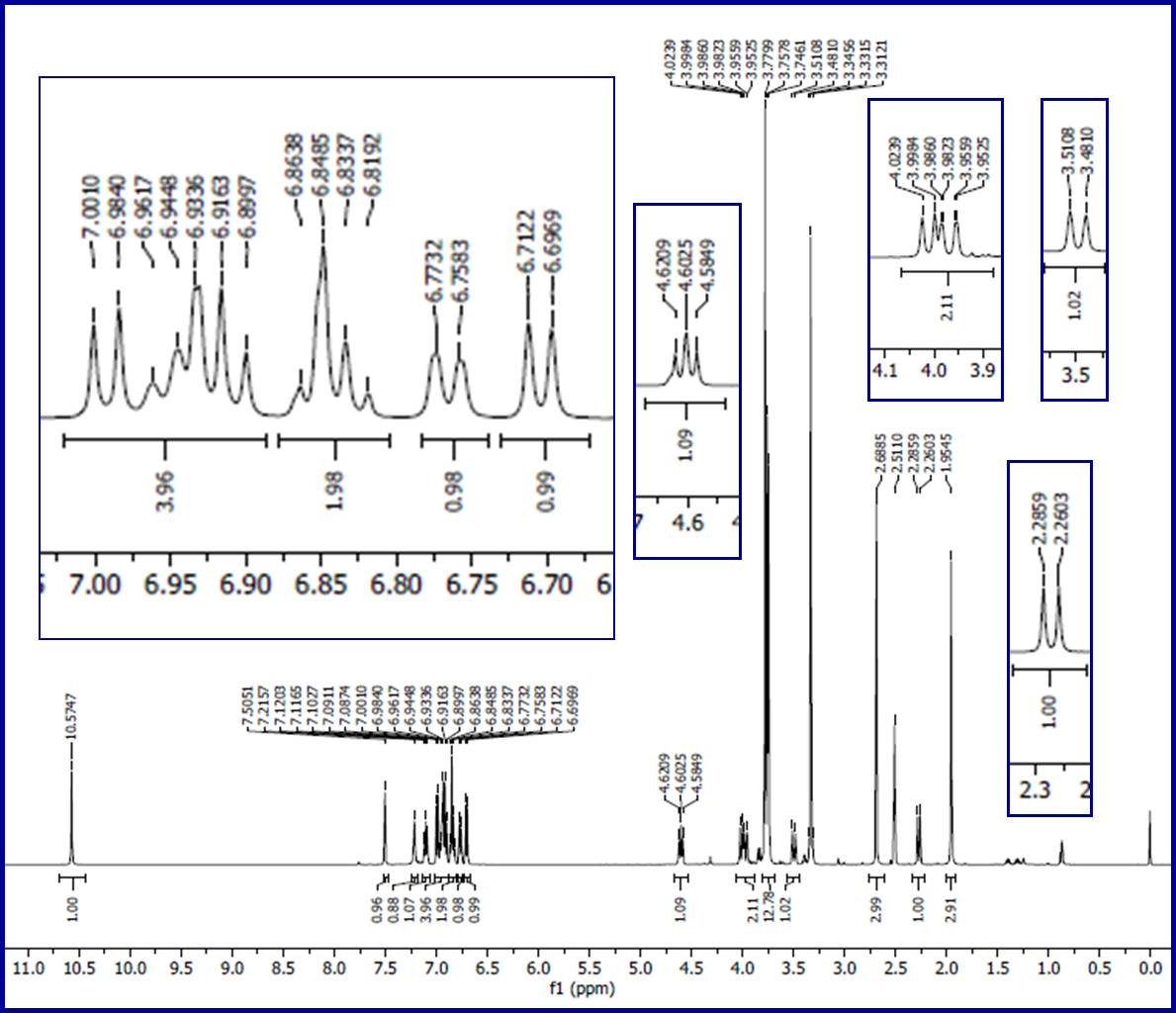


**Fig. S43.** ^1^H-NMR spectrum of compound **6n** in DMSO-*d_6_*.


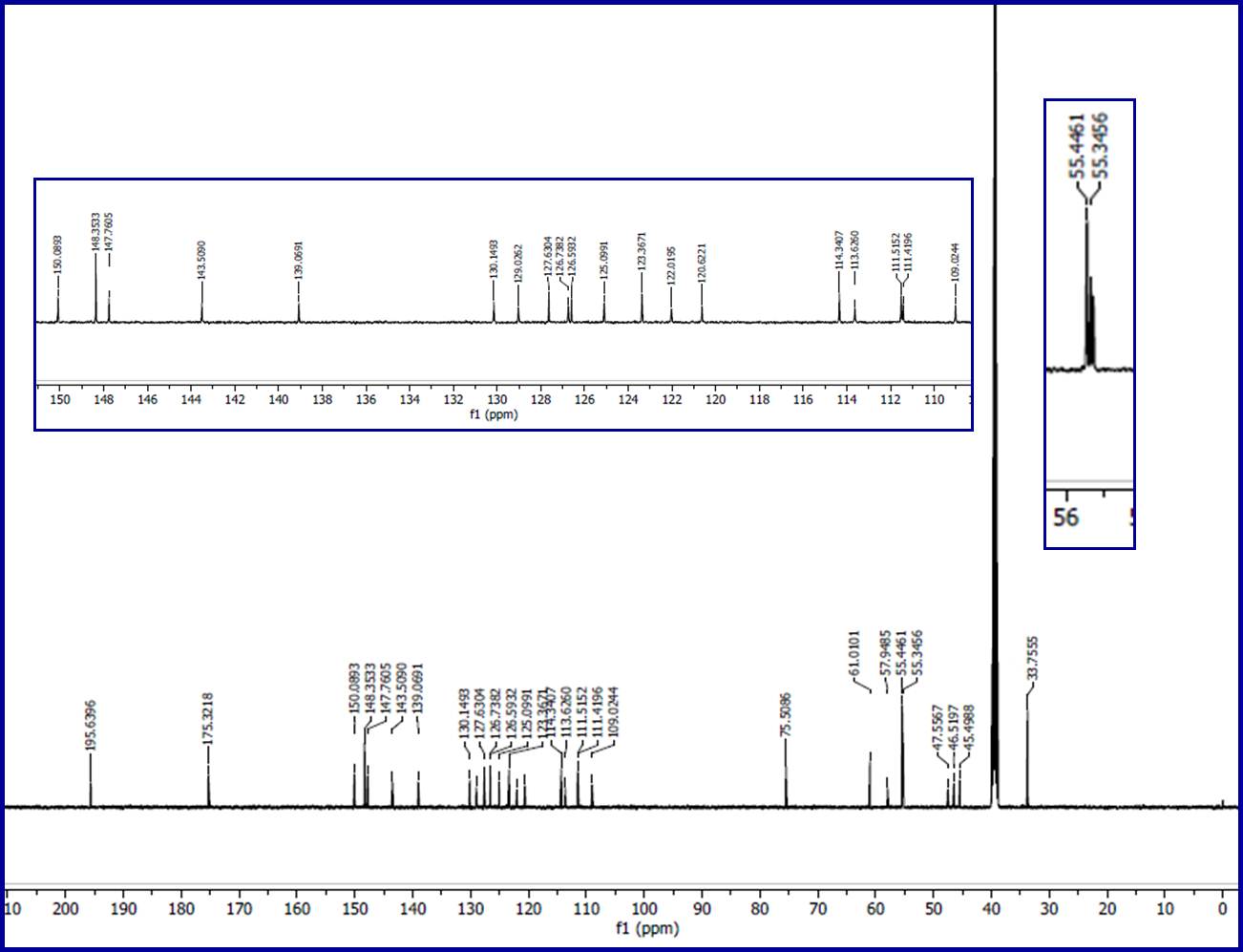


**Fig. S44.** ^13^C-NMR spectrum of compound **6n** in DMSO-*d_6_*.


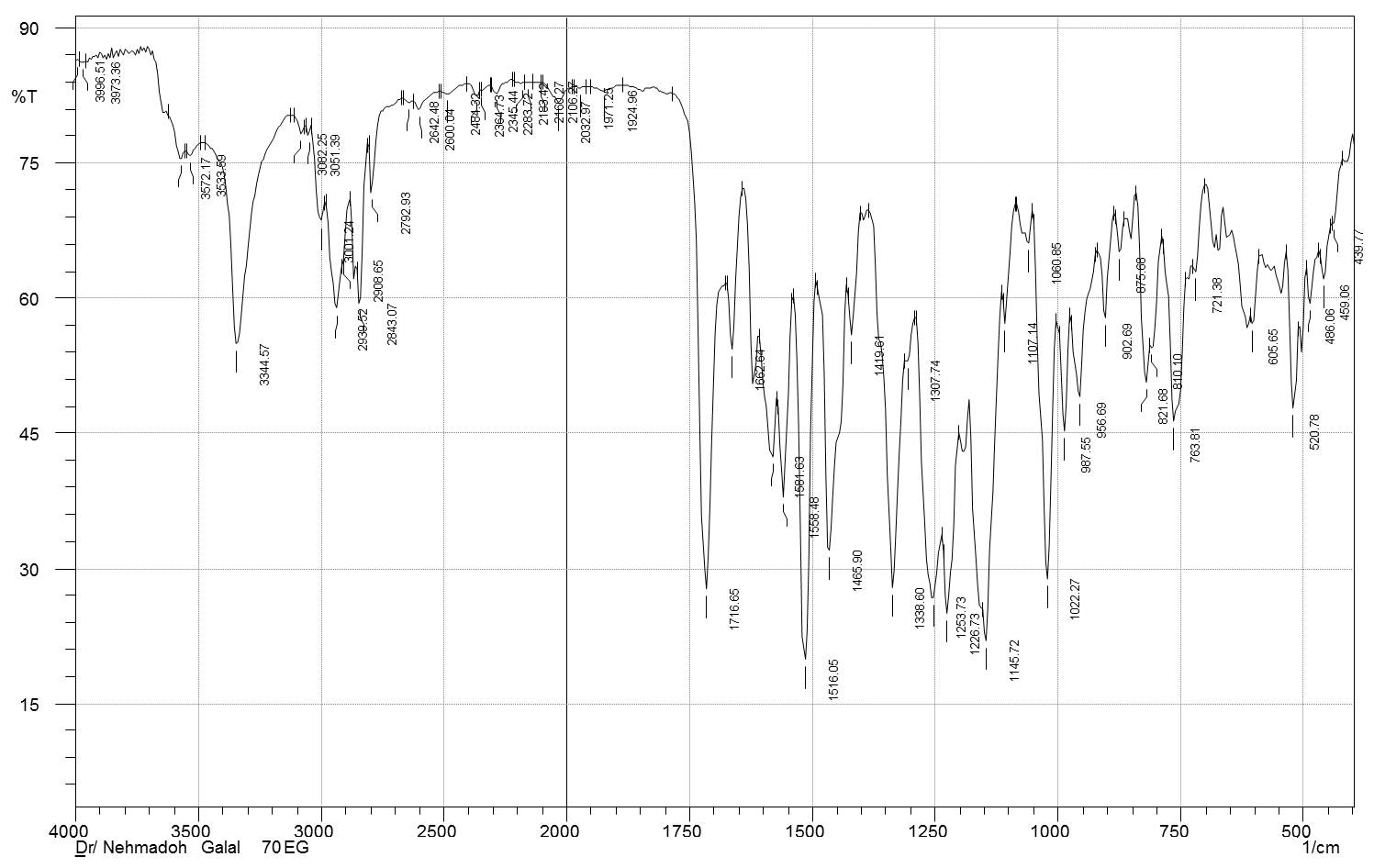


**Fig. S45.** IR spectrum of compound **6o** (KBr pellet).


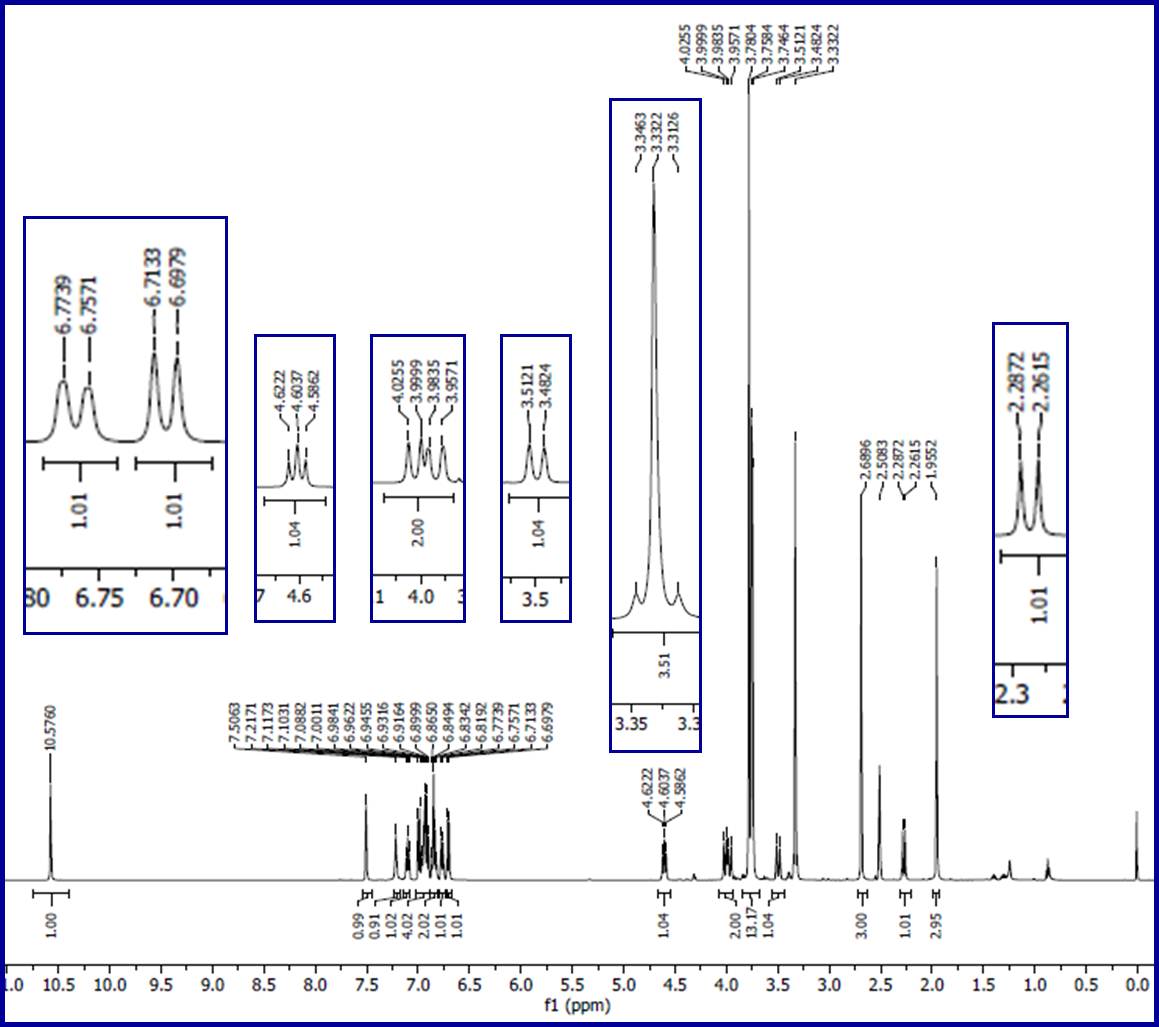


**Fig. S46.** ^1^H-NMR spectrum of compound **6o** in DMSO-*d_6_*.


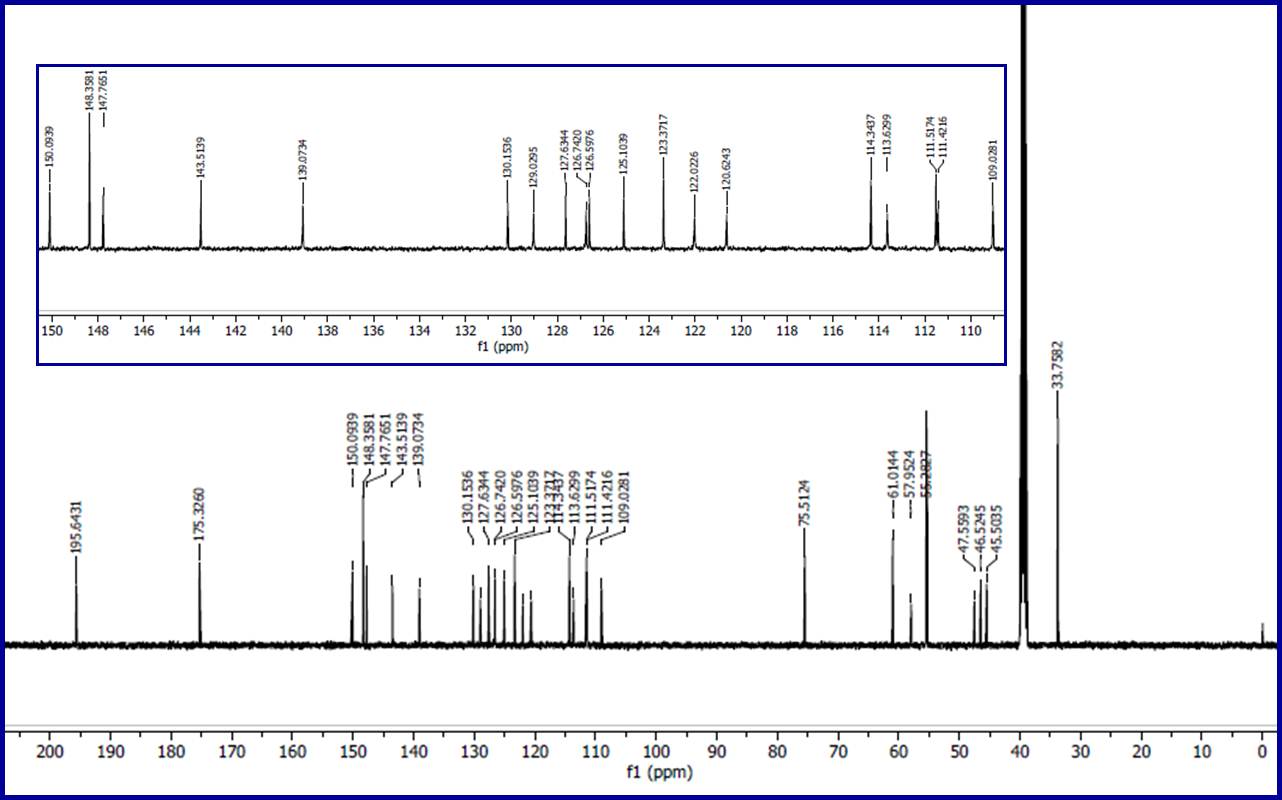


**Fig. S47.** ^13^C-NMR spectrum of compound **6o** in DMSO-*d_6_*.

**Fig. S48.** Dose-response curve for the tested compounds against MCF7 (breast cancer) cell line.

**Fig. S49.** Dose-response curve for the tested compounds against HCT116 (colon cancer) cell line.

**Fig. S50.** Dose-response curve for the tested compounds against A431 (skin squamous) cancer cell line.

**Fig. S51.** Dose-response curve for the tested compounds against PaCa (pancreatic cancer) cell line.

**Fig. S52.** Dose-response curve for the tested compounds against RPE1 (retinal pigment epithelium) cell line.

**
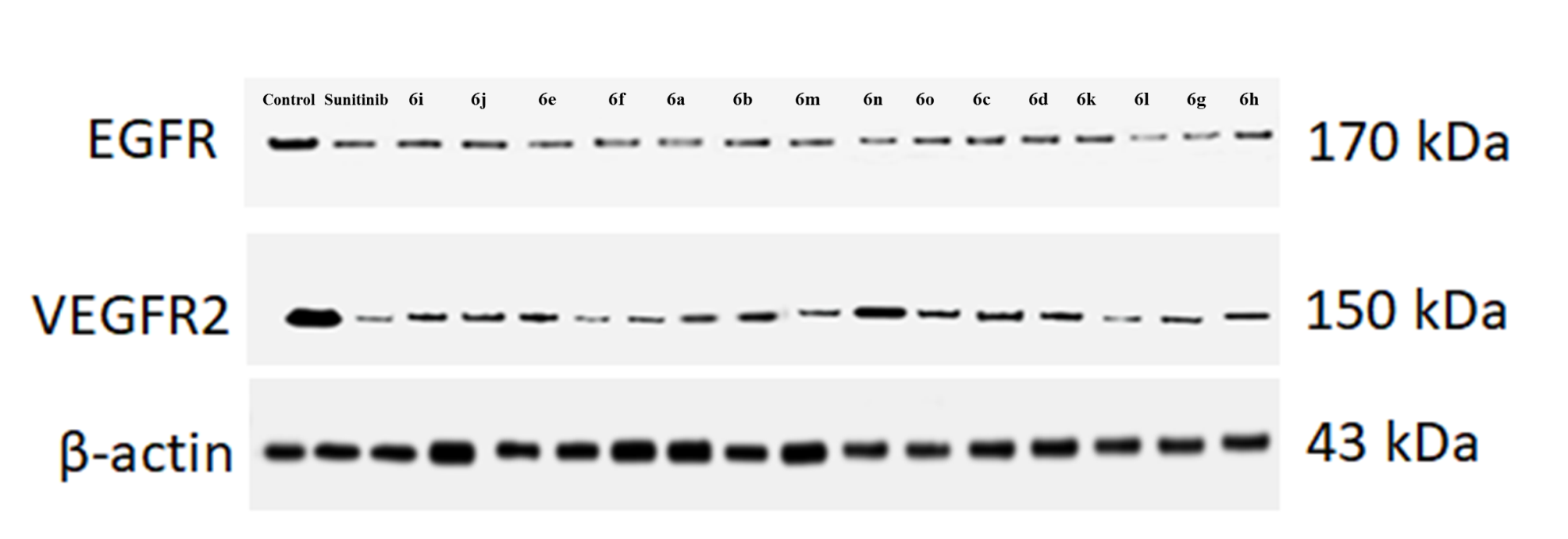
**

**Fig. S53.** Western blot of the tested compounds against EGFR and VEGFR-2 utilizing MTT-IC_50_ values against MCF7 (breast) cancer cell line.


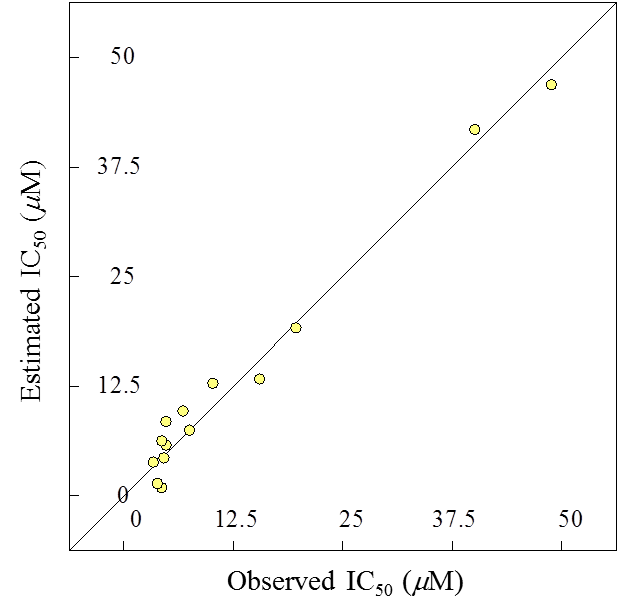


**Fig. S54.** QSAR plot representing the observed versus predicted IC_50_ (*μ*M) for the synthesized agents against MCF7 (breast) cancer cell line.

**
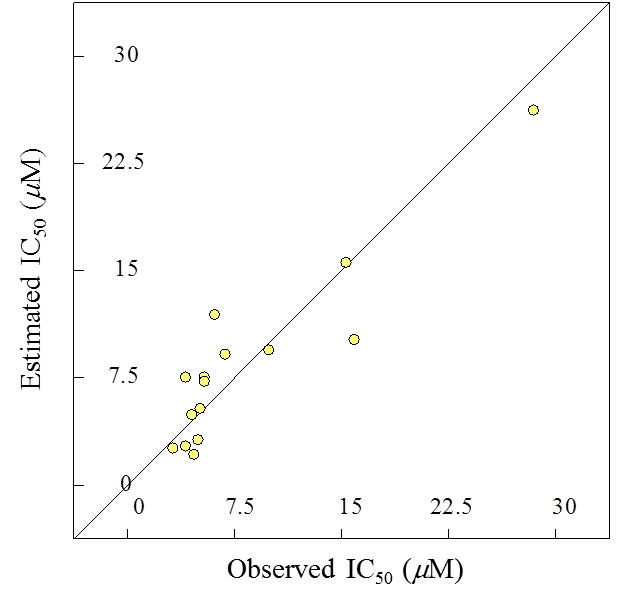
**

**Fig. S55.** QSAR plot representing the observed versus predicted IC_50_ (*μ*M) for the synthesized agents against HCT116 (colon) cancer cell line.


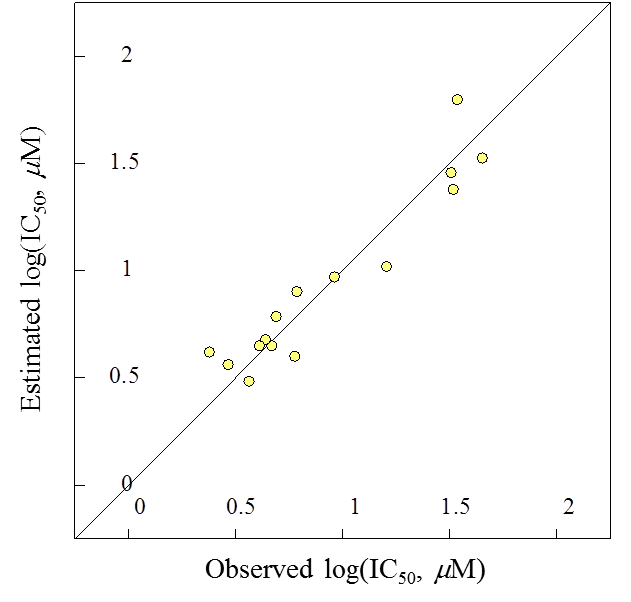


**Fig. S56.** QSAR plot representing the observed versus predicted log(IC_50_, *μ*M) for the synthesized agents against A431 (skin squamous) cancer cell line.


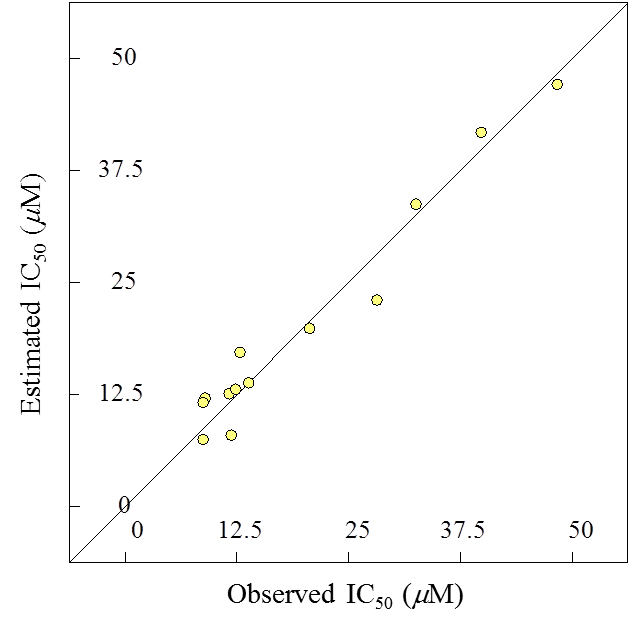


**Fig. S57.** QSAR plot representing the observed versus predicted IC_50_ (*μ*M) for the synthesized agents against PaCa2 (pancreatic) cancer cell line.


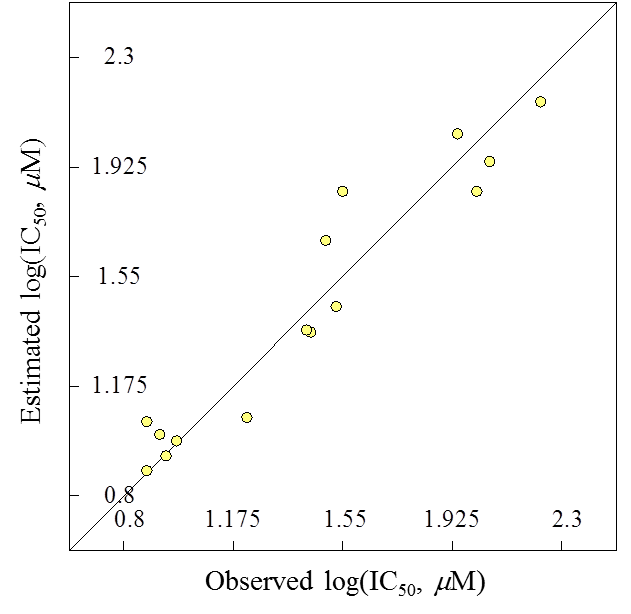


**Fig. S58.** QSAR plot representing the observed versus predicted log(IC_50_, *μ*M) for the synthesized agents against SARS-CoV-2.
